# Development and Evaluation of Exosporium-Anchored Bioluminescent and Fluorescent Reporters for Tracking *Clostridioides difficile* Spores Formed *In Vivo*

**DOI:** 10.1021/acssynbio.5c00961

**Published:** 2026-05-15

**Authors:** Osiris K. Lopez-Garcia, Trey Hejtmancik, Marjorie Pizarro-Guajardo, Lindsey Brehm, Christian Brito-Silva, Daniel Paredes-Sabja

**Affiliations:** † Department of Biology, 14736 Texas A&M University, College Station, Texas 77843, United States; ‡ Interdisciplinary Graduate Program in Genetics & Genomics, Texas A&M University, College Station, Texas 77843, United States; § ANID − Millennium Science Initiative Program − Millennium Nucleus in the Biology of the Intestinal Microbiota, Santiago 8370035, Chile

**Keywords:** *C. difficile* spores, bioluminescence, NanoLuc, fluorescent tags, tagging system, detection

## Abstract

*Clostridioides difficile* is an anaerobic spore-forming pathogen and a major cause of hospital-acquired and antibiotic-associated colitis. *C. difficile* spores are key for persistence and transmission of CDI, but methods to trace spores, including those produced during infection along the GI tract, are lacking. Here, we developed an exosporium-anchored reporter system that labels *C. difficile* spores during their formation by fusing NanoLuc or fluorescent proteins to the N-terminal domain of BclA1. The repaired BclA N-terminal domain (193 aa) supported stronger surface display and higher NanoLuc signal than the truncated 48 aa variant, without detectable changes in spore ultrastructure. NanoLuc-tagged spores were detectable *in vitro* down to ∼10^3^ spores in buffer and ∼10^4^ spores in fecal material, the latter reflecting matrix inhibition. In a murine model of CDI, the NanoLuc strain colonized and caused disease similar to wild-type, and fecal bioluminescence provided a qualitative readout of spore shedding once burdens exceeded ∼10^4^ spores per gram; however, luminescence did not quantitatively correlate with spore-based CFU in stools. By contrast, fluorescent reporters produced limited and unstable spore labeling, where *mScarlet-i3* accumulated in a subset of sporulating cells and was largely lost in mature spores, while *mNeonGreen* was obscured by intrinsic autofluorescence. However, in a murine model of CDI, a small proportion of spores were fluorescently tagged. These findings establish an exosporium-anchored NanoLuc reporter as a tool for detecting *C. difficile* spores *in vivo* and highlight the technical barriers that currently limit fluorescent tagging of mature spores.

## Graphical abstract

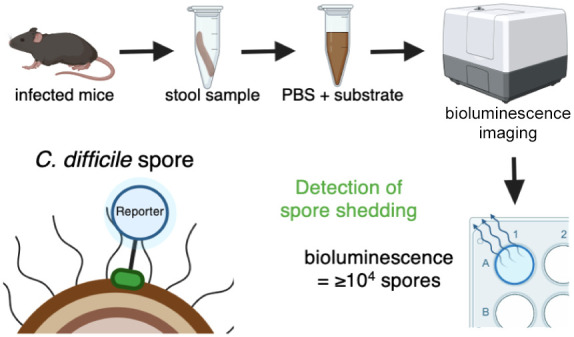

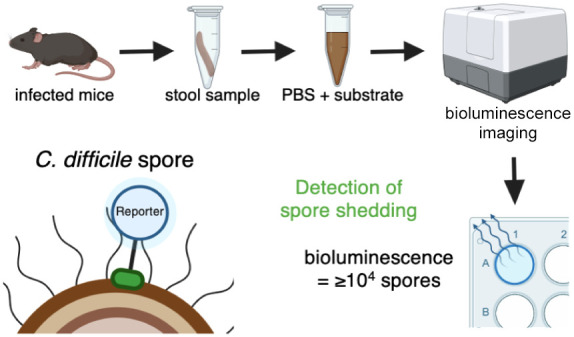

## Introduction

*Clostridioides difficile* infections (CDI) can range from mild diarrhea to severe conditions like fulminant colitis and toxic megacolon, often leading to bowel perforation, sepsis, and even death.^1–4^ The Center for Disease Control and Prevention (CDC) has classified *C. difficile* as an “urgent threat” due to the high number of cases (approximately 223,900 per year) and mortality rates of around 5–8% in the U.S.^1,5^ Recurrence of CDI (R-CDI) remains a major clinical challenge, where 15–30% of patients relapsing after initial treatment and a substantial fraction (∼60%) progressiing to multiple recurrent episodes.^6,7^ R-CDI contributes to an increased healthcare burden and elevated mortality risk (∼16–39%).^1,5–8^

As an obligate anaerobe, *C. difficile* relies on dormant spores for transmission and persistence within the host.^9^ *C. difficile* spores can adhere to intestinal epithelial cells, access basolateral receptors exposed during toxin-mediated barrier disruption, and gain entry into epithelial cells through interactions with fibronectin, vitronectin, and E-cadherin.^10,11^ *C. difficile* spores have been detected within intestinal tissues, where they may evade clearance and act as a spore reservoir for disease recurrence.^11^ Although these phenotypes support the critical role of *C. difficile* spores in disease recurrence,^10–13^ the field lacks tools to trace the location of spores during the early stages of infection and to allow their detection longitudinally along the GI tract within the infected host.

Several existing methods offer only partial solutions. Culture-based enumeration reports viable vegetative cells and spores^14^ but provides no spatial information or separation of inoculum spores from those produced during infection. Quantitative PCR (qPCR) has been used to quantify total *C. difficile* DNA from cells in stools, but this method is unable to distinguish live from dead cells and misses spores without specialized lysis procedures.^15–17^ Confocal microscopy can visualize adherence or intracellular spores in tissue and identify colonization sites but requires animal sacrifice and cannot determine the origin of the spores^11,18^ unless extremely low infectious doses are used. As a result, none of these approaches enable tracking *C. difficile* spores produced within the gastrointestinal tract.

Bioluminescence imaging (BLI) has emerged as a powerful, noninvasive method for monitoring bacterial populations *in vivo*.^19–23^ Luciferases such as Firefly, Click Beetle, Renilla, or NanoLuc have been used in pathogens including *S. aureus*,^21,23–27^ *E. coli*,^28,29^ *S. enterica*,^30–33^ and *L. monocytogenes* ^34–36^ to track dissemination, tissue localization, and infection dynamics.^19–23^ In these systems, luciferase is expressed intracellularly, and emitted light reports on the bulk global burden and spatial distribution of reporter-expressing cells. In *C. difficile*, codon-optimized NanoLuc and split-luciferase systems have been used for diagnostic assays that detect proteases and toxins in stool and, therefore, reflect vegetative cell activity.^37–42^ Fluorescent reporters, including the codon-optimized *mNeonGreen* and *mScarlet-i3*, have been used to examine single-cell transcriptional heterogeneity in *C. difficile* vegetative and early sporulating cells, including toxin- and sporulation-associated subpopulations.^43^ However, neither BLI nor fluorescent reporters have been used to directly tag *C. difficile* spores formed *in vivo*, leaving the physiology, localization, and persistence of newly produced spores unexplored.

In this study, we developed a spore surface tagging approach to label *C. difficile* spores produced *in vivo* by fusing NanoLuc or fluorescent proteins to the N-terminal domain of *bclA1* to anchor reporters to the exosporium during late sporulation. Using the sporulation-specific *cdeC* promoter, the repaired BclA1 N-terminal domain (193 aa), rather than the pseudogenized variant (48 aa), produced higher spore-associated luminescence *in vitro*. This NanoLuc system showed an approximately linear relationship between bioluminescence output and spore number within the range of 10^3^–10^7^ spores in buffer, whereas in fecal material, matrix inhibition shifted the detection limit upward to ∼10^4^ spores/g feces. In a murine model of CDI, the NanoLuc tag did not affect colonization or disease severity and allowed qualitative detection of spore shedding once the burden exceeded ∼10^4^ spores/g in feces. Fluorescent tagging with *mNeonGreen* was masked by autofluorescence, while *mScarlet-i3* showed reporter expression in a subset of sporulating cells with poor signal retention in mature spores and limited detection in infected tissues. These results establish a spore-surface tagging system that specifically labels *C. difficile* spores produced *in vivo* and demonstrate that NanoLuc fusions enable qualitative monitoring of spore shedding during infection, whereas current fluorescent reporters require further optimization for stable detection of mature spores.

## Materials and Methods

### Bacterial Strains and Growth Conditions

*Escherichia coli* strains (Table S1) were grown aerobically at 37 °C in Luria–Bertani (LB) medium with appropriate antibiotics. *C. difficile* strains (Table S1) were grown anaerobically at 37 °C in BHIS broth or on BHIS agar within a Coy anaerobic chamber. CDMM was prepared from sterile-filtered concentrated stocks of amino acids, salts, glucose, trace metals, iron, and vitamins, mixed to the final working concentrations previously described, with agar added for plates and uracil/5-FOA included as indicated for selections^44–46^ (extended methods in Supplemental Information).

### Construction of R20291_CM196_ Δ*pyrE* *C. difficile* strain

To explore the initial constructs, we utilized a hypersporulating strain derivative of R20291. This strain has a point mutation in the RsbV anti-sigma factor that triggers the early onset of sporulation, leading to high yields of spores.^47^ The Δ*pyrE* allele-exchange cassette was assembled into pMTL-YN4 by Gibson assembly using Δ*pyrE* PCR products from R20291 Δ*pyrE*, followed by Sanger sequence confirmation.^45^ The plasmid was conjugated from *E. coli* CA434 into R20291_CM196_ (Table S1, Table S2), with selection for single-crossover integrants on BHIS with thiamphenicol, cefoxitin, and cycloserine, and counter-selection on CDMM with 5-FOA and uracil to isolate Δ*pyrE* mutants.^44–46^ Thiamphenicol-sensitive colonies were screened by PCR for *pyrE* deletion and *lacZ-α* insertion, assessed by growth curves, and validated by whole-genome Illumina sequencing; *pyrE* restoration used pMTL-YN2 and the same conjugation, selection, PCR, growth, and sequencing workflow (Figure S1) (extended methods in Supplemental Information).

### Plasmid Construction

Reporter plasmids were constructed using restriction-ligation and/or Gibson assembly into pBlue-Heron, pMTL-YN2C, or pMTL-YN2C-TT backbones (Table S2) using primers listed in Table S3.^48^ Constructs encoded 48-aa- or 193-aa-ntd_*bclA1*_ fusions to *nLuc*, *mScarlet-i3*, or *mNeonGreen* under *bclA1* or *cdeC* promoters, with or without *tetO* operators and *tetR* under the rubrerythrin promoter where indicated. Native *bclA1* coding sequence SNPs in R20291 were repaired by multifragment Gibson assembly. EcoRI-NcoI fragments from pBlue-Heron plasmids were subcloned into pMTL-YN2C to create *C. difficile* shuttle constructs, and variants with *pyrE* terminators or fluorescent reporters were built analogously. All plasmids were sequence-verified by Sanger or nanopore sequencing (extended methods in Supporting Information).

### Construction of *C. difficile* Reporter Strains

Sequence-verified pMTL-YN2C-based shuttle plasmids encoding inducible or native promoter-driven spore surface reporters were transformed into *E. coli* CA434 and conjugated into R20291_CM196_ Δ*pyrE* or R20291_CM210_ Δ*pyrE* (Tables S1 and S2). Transconjugants were selected on BHIS with thiamphenicol, cefoxitin, and cycloserine, then cycled through single-crossover isolation, *pyrE* restoration on CDMM, thiamphenicol-sensitivity screening, and PCR confirmation of *pyrE* and reporter insertion. Clones without growth defects by growth-curve analysis were subjected to whole-genome sequencing to confirm on-target allelic exchange and the absence of off-target mutations. Shuttle plasmids carrying inducible spore surface reporter systems were used to conjugate sequence-confirmed constructs into R20291_CM196_ *C. difficile* strain. Plasmids carrying spore surface reporter constructs (*48aa-ntd*_*bclA1*_ *-nLuc* or *193aa-ntd*_*bclA1*_ *-nLuc* under the control of the native *cdeC* promoter) and plasmids carrying *mNeonGreen* fluorescent reporter constructs were conjugated into the R20291_CM196_ Δ*pyrE* strain. For strains used for *in vivo* infection (*193aa-ntd*_*bclA1*_ *-nLuc*) and fluorescent reporter strains with *mScarlet-i3*, conjugation was performed in the R20291_CM210_ Δ*pyrE* strain (extended methods in Supplemental Information).

### *C. difficile* Growth Curves

Frozen stocks were streaked on BHIS, single colonies were inoculated into BHIS, and overnight cultures were diluted 1:100 into BHIS with glucose and taurocholate for an 8-h preculture. These cultures were then diluted 1:100 into fresh BHIS, dispensed into 96-well plates, and OD_600_ was recorded over 20 h at 37 °C under anaerobic conditions using a microplate reader (extended methods in Supplemental Information).

### Sporulation Efficiency Using Phase-Contrast Microscopy

Strains (Table S1) were grown overnight in BHIS, subcultured, and spread onto 70:30 sporulation agar for a 16-h anaerobic incubation. Harvested cultures were resuspended in PBS, mounted on agarose pads, and imaged by phase-contrast microscopy; at least 300 cells per strain were manually classified as vegetative, sporulating, or mature spores based on phase brightness and morphology. Sporulation efficiency was calculated as the percentage of each category relative to the total cells counted (extended methods in Supporting Information).

### Purification of *C. difficile* Spores

Exponentially growing *C. difficile* cultures were spread onto 70:30 sporulation plates and incubated anaerobically for 5 days.^18^ Spores were harvested into ice-cold water, incubated at 4 °C overnight, then repeatedly washed and pelleted until >99% phase-bright spore purity was achieved by microscopy. Purified spores were counted using the hemocytometer and stored at −80 °C in aliquots (extended methods in Supplemental Information).

### Bioluminescence Assays with Purified Spores

Purified spores were diluted in PBS, dispensed into white 96-well plates, and mixed with Nano-Glo Luciferase Assay Reagent reconstituted at a 1:50 substrate-to-buffer ratio unless otherwise specified.^49^ Bioluminescence was recorded for 30 min at 460 nm emission in a microplate reader; the dynamic range was assessed by serial dilution of spores and titration of the Nano-Glo reagent over a wide range of substrate-to-buffer ratios (extended methods in Supporting Information).

### Dynamic Range of Bioluminescence of Spores in Fecal Samples

Fresh feces were suspended in PBS, mixed with defined spore dilutions to achieve a fixed fecal concentration, and tested either undiluted or after serial dilution. Samples were dispensed into white plates, Nano-Glo reagent (1:100) was added, and luminescence was measured for 30 min at 460 nm. Undiluted and diluted fecal-spore mixtures were compared to evaluate signal linearity and sensitivity in fecal matrices (extended methods in Supporting Information).

### Transmission Electron Microscopy

Purified spores were fixed in glutaraldehyde, post-fixed in osmium tetroxide, dehydrated in graded acetone, and embedded in modified Spurr’s resin using a microwave-assisted vacuum protocol.^50,51^ Ultrathin sections were mounted on carbon-coated grids, stained with uranyl acetate and lead citrate, and imaged at 100 kV on a JEOL 1200 EX TEM equipped with a CCD camera at the Texas A&M Microscopy and Imaging Center (extended methods in Supplemental Information).

### Western Blotting

Purified spores were extracted in USD buffer, boiled in SDS sample buffer, and separated by SDS-PAGE before being transferred to nitrocellulose membranes. Blots were blocked in BSA/T-TBS and probed with anti-NanoLuc or anti-SleC primary antibodies, followed by HRP-conjugated secondary antibodies, with chemiluminescent detection on a digital blot scanner (extended methods in Supporting Information).

### Murine Model of CDI

C57BL/6 mice received a defined oral antibiotic cocktail for 3 days, followed by a clindamycin injection, and were then gavaged with 1 × 10^5^ *C. difficile* spores (wild type, NanoLuc reporter strain, or PBS mock).^11,14,52^ Animals were singly housed and monitored daily for weight, clinical signs, diarrhea scores, and fecal *C. difficile* vegetative cells and spores, with humane end points enforced; selected cohorts were euthanized on Day 3 for cecal burden measurements^11,14,52^ (extended methods in Supplemental Information).

### Quantification of *C. difficile* Vegetative Cells and Spores from Feces and Cecum

Fecal and cecal samples were hydrated in prereduced PBS and serially diluted for vegetative CFU enumeration on CCFA plates.^52^ For spore counts, aliquots were ethanol-treated, plated on taurocholate-supplemented TCCFA plates, incubated anaerobically, and CFU were expressed as log_10_ CFU per gram of feces or cecal content (extended methods in Supporting Information).

### Bioluminescence from Feces and Cecum

Hydrated fecal and cecal suspensions were diluted, loaded into white plates, and mixed with Nano-Glo reagent at a 1:100 substrate-to-buffer ratio. Luminescence was recorded for 30 min at 460 nm, and daily positivity thresholds were defined as μ + 2.81 × σ of mock control readings to classify samples as bioluminescence-positive (extended methods in Supplemental Information).

### PCR Ribotyping and Confirmation of the *nLuc* Fragment in Stool-Derived Strains

Genomic DNA from stool-derived *C. difficile* isolates was extracted using phenol–chloroform, and PCR ribotyping was performed by amplifying the 16S-23S rRNA intergenic spacer region.^53^ The presence of *nLuc*-containing fragments was confirmed by PCR with *pyrE* and ntd_*bclA1*_/*nLuc*-specific primers and the visualization of products using agarose gel electrophoresis (extended methods in Supporting Information).

### Fluorescent Microscopy of Fluorescent Spore Surface Reporter Strains

Sporulating cultures grown on 70:30 plates were harvested, washed, stained with MTG (for membranes) and DAPI (for DNA), and fixed with paraformaldehyde.^43^ Fixed cells were mounted on agarose pads and imaged by phase-contrast and epifluorescence microscopy. Single cells at defined developmental stages were manually segmented in Fiji, and fluorescence intensity per cell was normalized to area for quantitative comparisons (extended methods in Supporting Information).

### Imaging of Fluorescent Spore Surface Reporter Strains during CDI

Antibiotic-pretreated mice were infected with wild-type, *mScarlet-i3*, or *mNeonGreen* spore surface reporter strains and monitored as in the NanoLuc infection experiment.^11,14,52^ On day 3 postinfection, mice were euthanized, and colon and cecum tissues were collected for immunostaining and confocal imaging of tissue-associated fluorescent spores (extended methods in Supporting Information).

### Tissue Fixation, Immunofluorescence Staining, and Confocal Imaging

Colonic and cecal tissues were fixed in paraformaldehyde-sucrose, permeabilized with Triton X-100, and blocked in BSA before incubation with antispore antibodies, phalloidin conjugates, and DAPI. After washing and mounting, tissues were imaged on a Stellaris 5 confocal microscope using standardized laser and gain settings, acquiring z-stacks through the mucosal surface. Spores were classified and counted in LAS X 3D Analysis software (Leica Microsystems, Germany) as total spores and reporter-positive to quantify detection efficiency (extended methods in Supporting Information).

### PCR Ribotyping and Confirmation of *mScarlet-I3* or *mNeonGreen* Fragment in Stool-Derived Strains

Stool-derived isolates from fluorescent reporter infections were subjected to phenol–chloroform DNA extraction and PCR ribotyping as described above.^53^ PCR with *pyrE* and reporter-specific primers (for *mScarlet-i3* or *mNeonGreen*) was used to confirm the maintenance of the fluorescent reporter cassette, with products analyzed by agarose gel electrophoresis (extended methods in Supporting Information).

### Statistical Analysis

Statistical tests were performed in GraphPad Prism 10 using ANOVA (ordinary, Welch/Brown-Forsythe, or repeated-measures with Geisser-Greenhouse correction), mixed-effects models, Šídák, Dunnett, Tukey, or Welch’s t-tests, as appropriate to the data structure. Normality was assessed where appropriate, and significance thresholds were set at *P* < 0.05, with additional thresholds indicated by standard asterisk notation and letter codes for non-significant groupings (extended methods in Supplemental Information).

## Results

### Rationale and Development of a *C. difficile* Spore-Tagging System

To tag *C. difficile* spores during their development, we focused on late stages of sporulation when the coat and exosporium are assembled (Figure 1A).^54,55^ The developmental stages of the forespore are regulated by RNA polymerase sigma factors σ^F^ and σ^G^, whereas σ^E^ and σ^K^ control mother cell development^55–58^ (Figure 1A). Given that σ^K^ controls late mother cell events, including the expression of coat and exosporium genes,^56,57,59,60^ we narrowed spore-tagging protein candidates to those regulated by σ^K^. Among the candidates are three BclA collagen-like paralogs (Figure 1B), where BclA2 and BclA3 form parts of the hairs in R20291 spores^11,61^ and the N-terminal domain of all three proteins anchors to the spore surface.^62^ In epidemically relevant strains like R20291 (ribotype 027), *bclA1* is pseudogenized due to a nonsense mutation, resulting in a truncated protein of 48 aa corresponding to the N-terminal domain that is still localized on the spore surface (Figure 1B).^51,62^ In contrast, reference strains like 630 encode a full-length protein of 694 aa, including an N-terminal domain of 193 aa (Figure 1B).^63^ BclA2 features a shorter NTD (∼5 aa) but an extended collagen-like region (CLR) of ∼121 GXX repeats (363 aa), while BclA3 has a longer NTD (∼46 aa) and a shorter CLR of ∼72 GXX repeats (216 aa) (Figure 1B).^64^ Both proteins share CTDs of similar size (∼14–15 kDa) and are immunogold-detected as part of the hair-like projections in R20291 spores.^11,64^ Additional spore surface proteins include CdeC, CdeM, CotE, and CotL, but these are implicated in coat/exosporium assembly and/or *in vivo* colonization,^18,65–67^ and translational fusions will likely yield nonfunctional proteins, impacting spore structure and function. Hence, we selected the N-terminal domain of BclA1, as it is likely a dispensable domain that would not interfere with spore assembly in the R20291 strain and could prove useful as a spore-labeling scaffold (Figure 1C).

**Figure 1:**
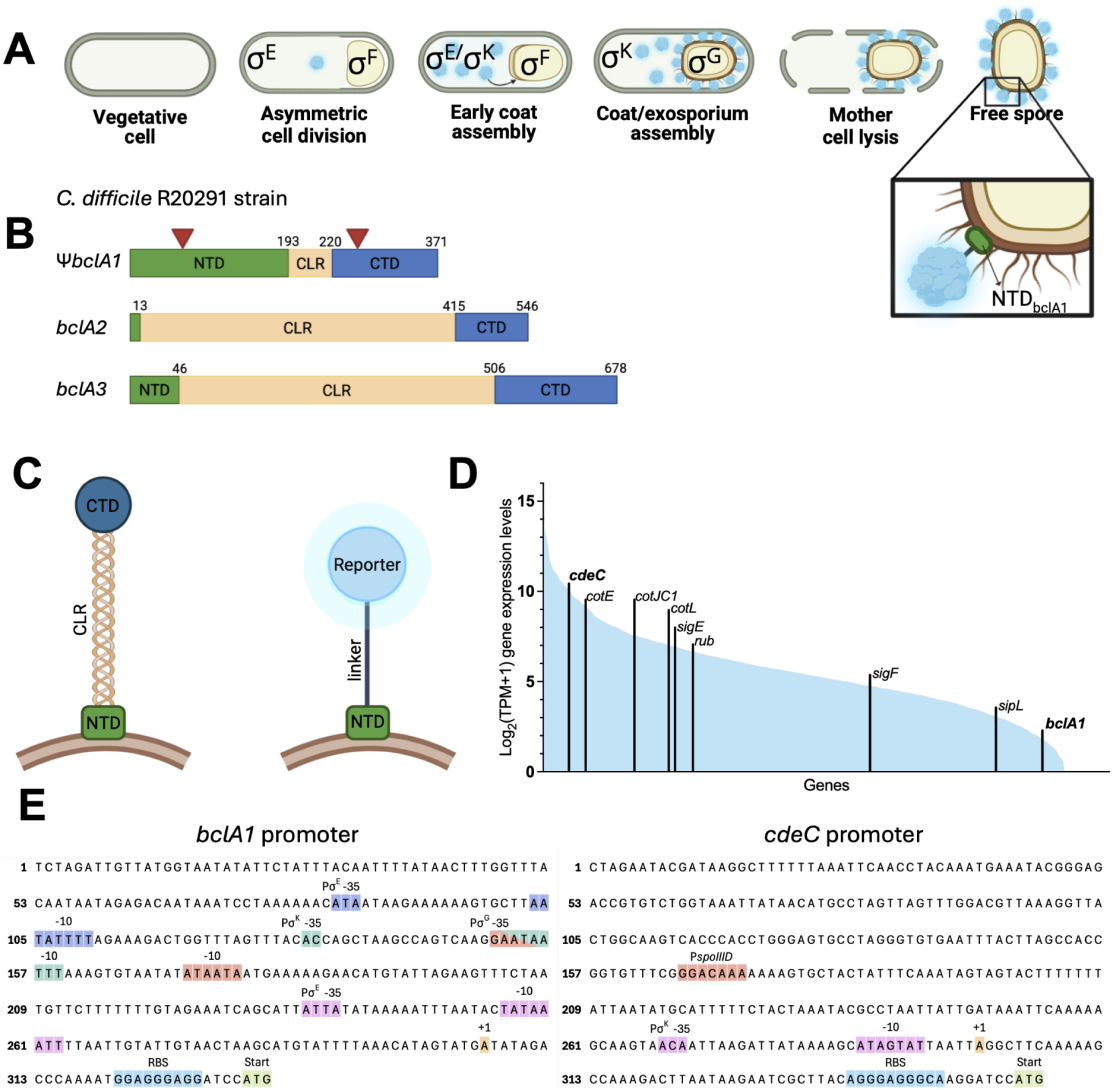
Design of surface tagging for *C. difficile* spores. (A) Schematics of the main developmental stages of *C. difficile* sporulation: vegetative cell growth, asymmetric cell division, early coat assembly, late coat/exosporium assembly, mother cell lysis, and release of mature spores. Relevant sigma factors activated at each stage and compartment are indicated. The zoomed-in view highlights the predicted anchoring of reporter proteins (nanoluciferase or fluorescent reporter). (B) Schematic illustration of the domains of BclA paralogs in *C. difficile* R20291 strain: N-terminal domain (NTD), collagen-like region (CLR), and C-terminal domain (CTD). Red triangles indicate pseudogenization sites in *bclA1*. (C) Schematic illustration of the utilization of the NTD of BclA for anchoring reporter proteins to the spore surface utilizing an anchoring protein. (D) RNA-seq analysis showing relative expression levels (Log_2_ TPM) of sporulation-related genes (*x*-axis) during the early stationary phase (12 h). The RNA-seq data for this figure were obtained from the GEO database (accession no: GSE107961: Gu et al., 2018 ^68^). (F) Promoter sequences of *cdeC* and *bclA1* show the predicted −35 and −10 regions for putative sporulation-specific sigma factors and SpoIIID-binding boxes. Ribosome binding sites (RBS) are marked in light blue and start codons are shown in green.

To select a promoter for reporter expression, we utilized publicly available R20291 RNA-seq datasets of transcription profiles during sporulation cultures to identify σ^E^/σ^K^-regulated coat and/or exosporium genes.^68^ Transcriptional profiling showed that the exosporium morphogenetic gene *cdeC* (CDIF27147_01092) was strongly expressed during sporulation (log_2_(TPM+1) = 10.1) (Figure 1D-E). Other σ^E^/σ^K^-regulated coat and exosporium genes, such as *cotE* (CDIF27147_01458), *cotJC1* (CDIF27147_00673), *cotL* (CDIF27147_02914), and *sipL* (CDIF27147_03750) also showed increased expression during sporulation, but at levels lower than *cdeC* (Figure 1D). Moreover, *bclA1* (CDIF27147_00471), the selected anchoring NTD, had 10-fold lower expression levels (log_2_(TPM+1) = 1.83) than the *cdeC* promoter (Figure 1D-E). To guide expression of *ntd*_*bclA1*_, we selected P_*cdeC*_ given its highest expression among all σ^E^/σ^K^-regulated coat and exosporium genes. *cdeC* encodes a cysteine-rich exosporium morphogenetic protein that is highly conserved in the *Peptostreptococcaceae* family and is critical for assembling the outermost spore layer and ensuring barrier integrity in R20291.^67,69^ Additionally, P_*bclA1*_ was selected as a control for native expression of the tagging *ntd*_*bclA1*_ domain, despite its low activity. To enable reporter attachment while preserving the function of *ntd*_*bclA1*_, a flexible peptide linker (Gly-Ala-Ala-Gly-Gly-Ala-Gly-Gly-Gly-His), encoding three tandem glycine- and alanine-rich motifs, was inserted C-terminally to the NTD to spatially separate the anchoring domain from the fused reporter and reduce potential steric hindrance or folding interference.^70^ In summary, our expression-tagging approach consisted of using *ntd*_*bclA1*_ to anchor reporter proteins to the spore surface and driving its expression with the strong *P*_*cdeC*_ or the weak, native *P*_*bclA1*_.

### Development of a *tetR-*Mediated Regulated Spore-Tag

Tagging *C. difficile* spores formed during CDI requires a regulated expression system. Hence, we explored whether TetR-based regulation could be implemented to control the expression of a *P*_*cdeC*_/*P*_*bclA1*_-ntd_*bclA1*_-reporter system. For this, we utilized the bioluminescent reporter NanoLuc because of its small size (19.1 kDa) and ∼150-fold higher bioluminescence than Firefly or Renilla luciferases.^49^ Bioluminescence also allows for the quantification of expression regulation over a wide dynamic range. Expression of *tetR* was placed under the control of σ^A^-dependent *rubrerythrin* promoter (P_*rbr*_)^68^ (Figure S3A), which exhibits intermediate expression levels (log_2_(TPM+1) = 6.7), in between *cdeC* and *bclA1* (Figure 1D). To repress *tetR* and/or *nLuc* expression, *tetO* operator sequences were introduced into the promoter regions of *P*_*rbr*_, *P_bclA1_,* and *P*_*cdeC*_ (Figure S3A). Eight constructs containing all possible permutations were introduced into the *pyrE* loci of the R20291_CM196_ hypersporulating strain^47^ and confirmed by PCR and whole-genome sequencing (Figure S4). Prior work shows that ectopic expression in the *pyrE* loci has no impact on virulence;^11^ accordingly, we observed no differences in growth kinetics compared with the parental strain (Figure S5) The only exceptions were a modest decrease and increase in mature spore frequency, respectively, for the *tetR-P*_*rbr*_ *-P*_*cdeC*_ *-48aa-ntd*_*bclA1*_ *-nLuc* and *tetR-P*_*rbr*_ *-P*_*cdeC*_ *-tetO-48aa-ntd*_*bclA1*_ *-nLuc* constructs (Figure S3B–C). The rationale for these designs was to test whether TetR could repress *tetO*-containing promoters^71,72^ and whether anhydrotetracycline (aTc) would allow induction of *nLuc-*fusions.

Next, we examined how well each construct tagged *C. difficile* spores with bioluminescence (BL). For this, purified spores of each strain were prepared and assayed for BL. Results demonstrated that spores of strains with P_*cdeC*_-driven constructs had significantly higher BL than spores with P_*bclA1*_-driven constructs, with a ∼20-fold increase in signal (Fig S3D), consistent with the higher *cdeC* transcriptional levels (Figure 1D). Introducing *tetO* sites into the P_*bclA1*_-expressed construct resulted in spores with a 10-fold lower BL and a 1,000-fold reduction when *tetO* was introduced into P_*cdeC*_-expressed construct (Figure S3D). However, the addition of the aTc inducer did not restore BL to native promoter levels, as the signal remained at or near repressed levels (Figure S3D). The addition of *tetO* into P_*rbr*_ to autoregulate *tetR* did not improve BL tagging of spores (Figure S3D). Strains with *tetO* in P_*rbr*_ and native P_*bclA1*_ or P_*cdeC*_ showed, at most, a small aTc-dependent increase in BL spore signal, while having *tetO* sites in both P_*rbr*_ and P_*bclA1*_ or P_*cdeC*_ kept BL spore levels low even after aTc induction (Figure S3D). Under the configurations tested, the TetR/*tetO* system yielded strongly repressed *nLuc* fusion expression, while native P_*cdeC*_ yielded higher BL spore signal.

### Repaired NTD_BclA1_ Improves Dynamic Range of Spore Bioluminescence

We recently demonstrated that the pseudogenized 48-aa NTD_BclA1_ is predicted to form a linear alpha helix with no secondary structure; by contrast, the repaired 193-aa NTD_BclA1_ is predicted to form a globular domain, suggesting more efficient tagging of the spore surface. Therefore, having selected the promoter to drive *nLuc* expression, we constructed two NanoLuc fusions where the reporter was linked to either the truncated 48-aa N-terminal region of *bclA1* or a repaired 193-aa N-terminal domain, and both constructs were inserted at the *pyrE* locus of the R20291_CM210_ strain (Figures 2A and S6). Bioluminescence from purified spores showed that both the *48aa-ntd*_*bclA1*_ *-nLuc* and *193aa-ntd*_*bclA1*_ *-nLuc* strains produced BL signals that increased with spore concentrations across the tested range (10^1^–10^7^ per reaction) (Figure 2B). The *193aa-ntd*_*bclA1*_ *-nLuc* strain consistently yielded higher BL than the *48aa-ntd*_*bclA1*_ *-nLuc* reporter, with differences most pronounced at higher concentrations (≥10^5^ spores), where signals were up to ∼10-fold greater (Figure 2B). The repaired BclA1 N-terminal reporter improved the detection limit and sensitivity across the tested range.

**Figure 2:**
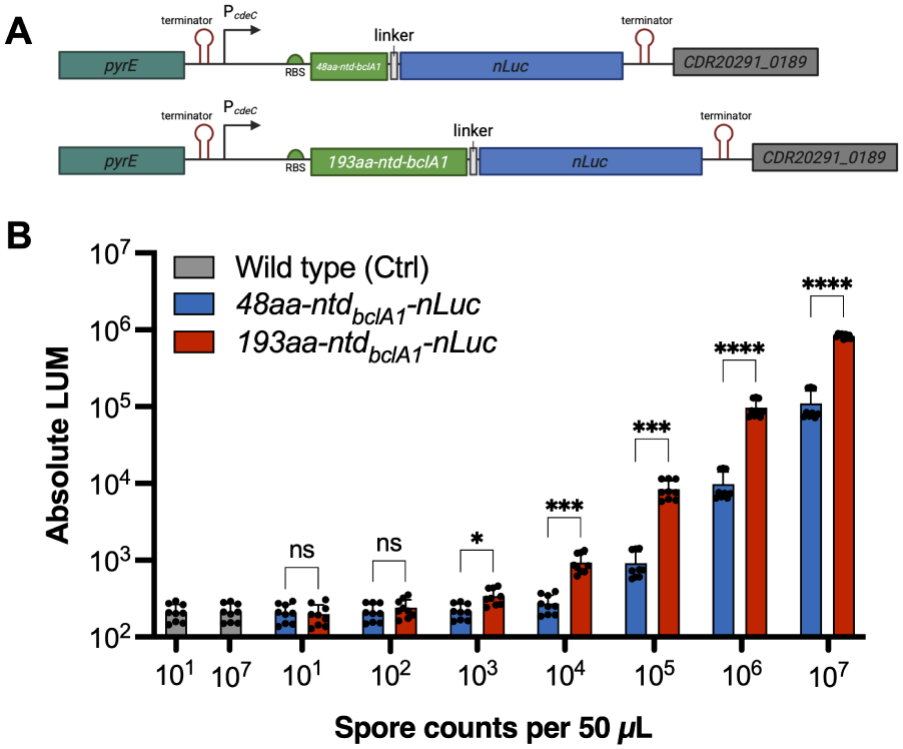
Dynamic range of bioluminescence expression in purified NanoLuc-tagged spores. (A) Design of spore-tagging constructs to anchor NanoLuc to the *C. difficile* spore surface. Constructs consist of a fusion of *48aa-ntd_bclA1_-nLuc* or a fusion of *193aa-ntd_bclA1_-nLuc* under the control of the *cdeC* promoter and integrated into the genome at the *pyrE* locus. (B) Bioluminescence measurements (absolute luminescence units, LUM) of purified spores expressing *nLuc* compared to wild-type (WT) spores as a control. Spores were serially diluted to a total amount of 10^7^ to 10^1^ and luminescence was quantified at 460 nm using a microplate reader. Data are presented as mean ± SD from three independent biological replicates, each with technical duplicates. Statistical significance was determined by Welch and Brown-Forsythe ANOVA, assuming Gaussian distribution with Dunnett’s multiple comparisons test. Significance levels are indicated as follows: *P* < 0.05 (*), *P* < 0.01 (**), *P* < 0.001 (***), and *P* < 0.0001 (****).

### NanoLuc Localizes to Spore/Exosporium Extracts and Spores Retain Wild-Type Ultrastructure

Localization of reporter proteins to spore surfaces can impact ultrastructure and, hence, spore functionality.^73,74^ Therefore, we evaluated whether the *193aa-ntd*_*bclA1*_ *-nLuc* fusion causes any major defects in spore assembly, rather than subtle ultrastructural variations, using transmission electron microscopy (TEM) of purified wild-type and *193aa-ntd*_*bclA1*_ *-nLuc* spores (n = 6 spores per strain). TEM images were examined for the qualitative appearance of the spore ultrastructure (Figure 3A and Fig. S7) revealing no major, obvious defects in spore assembly between wild-type and *193aa-ntd*_*bclA1*_ *-nLuc* strains (Figure 3A), suggesting that the reporter does not visibly disrupt spore ultrastructure. Immunoblots used spore coat/exosporium extracts prepared by the USD chemical extraction method, which specifically removes exosporium (e.g., CdeC, CdeM) and coat proteins while exposing cortex markers like SleC without causng inner spore damage, as validated by phase-contrast/TEM microscopy, western blotting, and lysozyme permeability assays.^75^ Anti-NanoLuc probing yielded an immunoreactive band at 25 kDa in the *193aa-ntd*_*bclA1*_ *-nLuc* strain but not in the wild type control (Figure 3, S8A-B). Parallel blots using an anti-SleC antibody, targeting a cortex-specific enzyme, and SDS-PAGE gels showed comparable loading and extraction between strains, with immunoreactive bands at ∼37 kDa (Figure 3, S8C-E), consistent with the expected SleC molecular weight.^76^ These results demonstrate that the *193aa-ntd*_*bclA1*_ *-nLuc* fusion is present in coat/exosporium extracts without qualitatively altering the spore ultrastructure.

**Figure 3:**
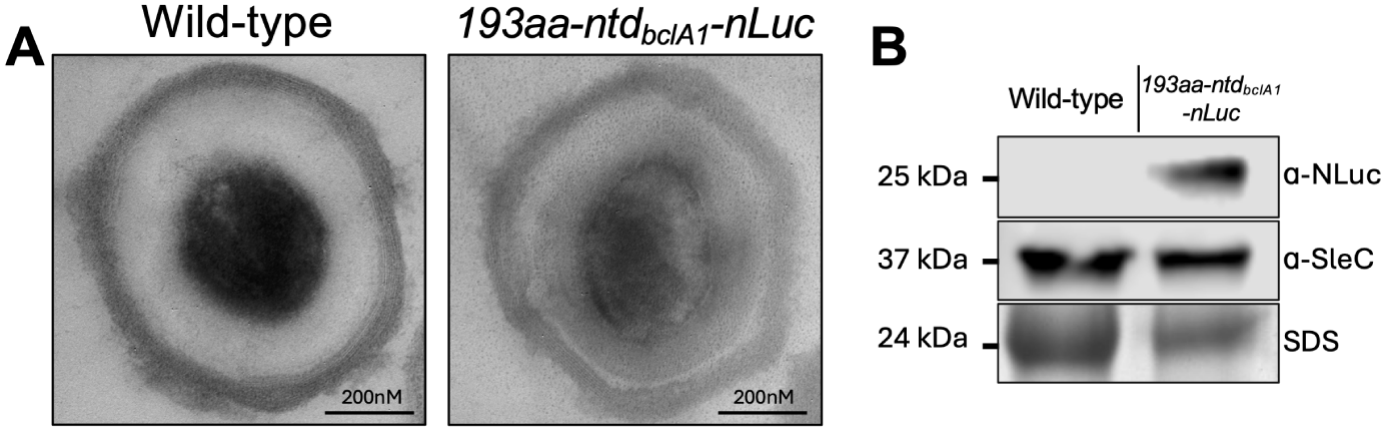
Transmission electron microscopy and immunoblotting of NanoLuc-containing*C. difficile* spores. (A) Representative TEM images of purified spores from WT and *193aa-ntd_bclA1_-nLuc* strains, captured using a JEOL 1200 EX microscope at 50,000× magnification. Scale bars represent 200 nm. These images are shown for qualitative assessment of spore and exosporium morphology. (B) Western blot analysis of spore coat and exosporium extracts prepared using USD chemical extraction from purified R20291_CM210_ WT and R20291_CM210_ Δ*pyrE/pyrE* + P*_cdeC_*-*193aa-ntd_bclA1_-nLuc* spores. Membranes were probed with antibodies against NanoLuc and SleC (loading control). A Coomassie-stained gel is shown as an additional loading control.

### Impact of Substrate Availability and Fecal Matrix on the Bioluminescence Signal of *C. difficile* NanoLuc-Tagged Spores

Because the NanoLuc reporter will be used to detect spores in stools and intestinal tissue, the effects of substrate concentration and fecal matrix on bioluminescence were evaluated. Nano-Glo substrate titration with *193aa-ntd*_*bclA1*_ *-nLuc* spores shows a strong substrate-dependent response, with the BL signal increasing from ∼4,000 a.u. up to ∼900,000 a.u. between the lowest (1:100,000) and highest (1:50) substrate dilutions (Figure 4A). This >200-fold broad dynamic range demonstrates that the *193aa-ntd*_*bclA1*_ *-nLuc* reporter remains detectable at low substrate concentrations, while higher levels substantially enhance BL output.

**Figure 4:**
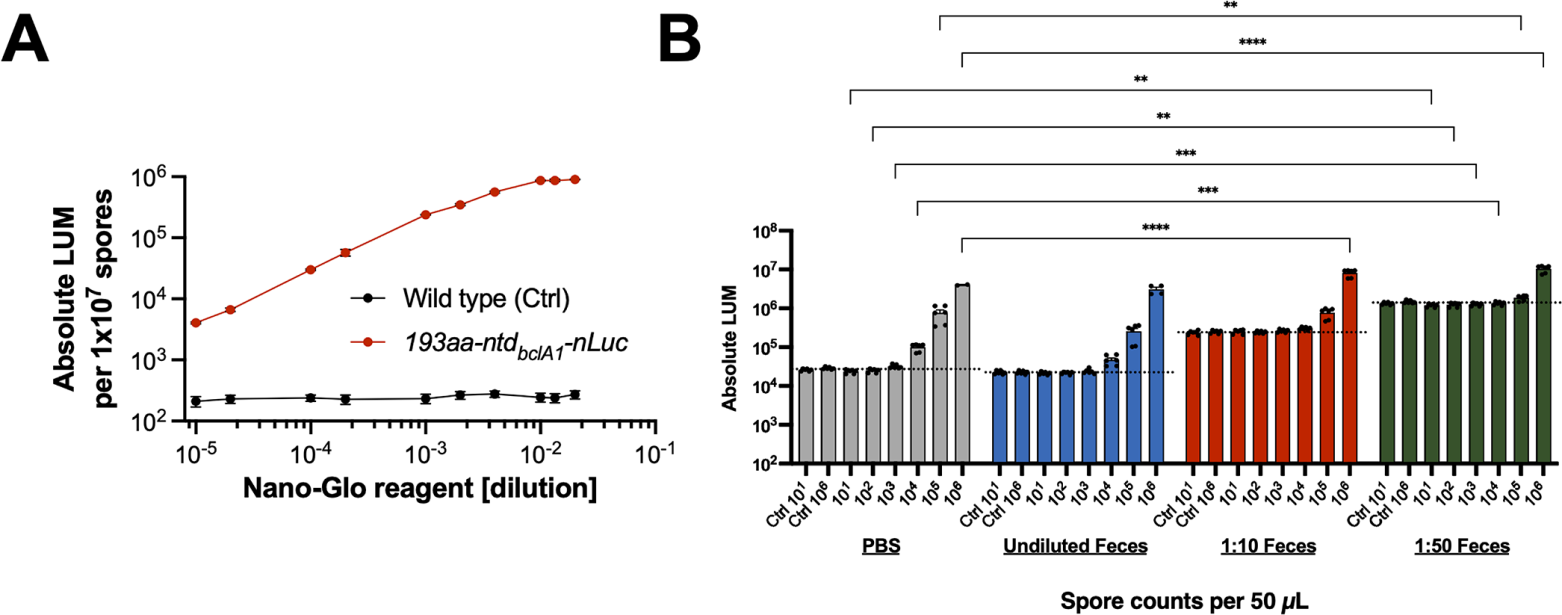
Optimization of Nano-Glo reagent and dynamic range analysis of NanoLuc-tagged *C. difficile* spores in fecal samples. (A) Nano-Glo reagent titration to optimize bioluminescence detection sensitivity for purified wild-type (WT) and *193aa-ntd_bclA1_-nLuc* spores. Purified spores were diluted to 1 × 10^7^ per reaction, incubated with serial dilutions of the Nano-Glo reagent prepared in a stepwise manner (see Methods), and luminescence values recorded across 460 nm using a microplate reader. Luminescence signals are expressed as absolute LUM per reaction. (B) Dynamic range of bioluminescence detection in fecal matter. 1*93aa-ntd_bclA1_-nLuc* spores were diluted in PBS or fecal suspensions to obtain a 3.2% w/v fecal concentration (“undiluted” sample). Further 1:10 and 1:50 dilutions of spore-fecal suspensions were made with PBS. Luminescence signals for 1:10 and 1:50 dilution samples were multiplied by the dilution factor to allow for direct comparison with PBS and undiluted conditions. Samples were aliquoted in technical duplicates, incubated with 1:100 Nano-Glo reagent, and bioluminescence recorded at 460 nm. Bars represent mean ± SD from three independent biological replicates; individual data points indicate technical replicates. Dotted lines represent noise as determined by the control. Statistical significance was determined by ordinary two-way ANOVA with Tukey’s multiple comparisons test. Significance levels are indicated as follows: *P* < 0.05 (*), *P* < 0.01 (**), *P* < 0.001 (***), and *P* < 0.0001 (****).

Fecal matter is a highly heterogeneous matrix that can reduce detectable BL by quenching emitted light and limiting substrate diffusion, thereby lowering assay sensitivity and dynamic range.^77,78^ To assess this effect, spores were diluted in PBS or in 3.2% (w/v) mouse fecal suspensions (Figures 4B and S9A-D). In PBS, the NanoLuc-tagged spores showed a log-linear increase in signal with rising spore numbers, with ∼40-fold higher BL at 10^6^ versus 10^4^ spores (Figure 4B). In 3.2% (w/v) fecal suspensions, BL at 10^1^–10^3^ spores was indistinguishable from noise, and at 10^4^–10^6^ spores signals were reduced by ∼1.3- to 3-fold relative to PBS, indicating matrix-dependent inhibition while still allowing detection at higher spore concentrations (Figure 4B). Diluting fecal suspensions with PBS modestly improved performance at high spore inputs (Figures 4B and S9A–D). To allow direct comparison of luminescence signals with PBS and undiluted conditions, resulting luminescence signal from 1:10 and 1:50 dilution samples was corrected multiplying by dilution factor. At 1:10 or 1:50 dilution, BL above noise was observed for ≥10^4^–10^6^ spores, but signals at these spore concentrations remained ∼2–3-fold lower than in PBS alone (Figure 4B). Additionally, diluting fecal samples reduces the effective spore concentration by the same factor (e.g., 10-fold for 1:10), requiring at least 10 times more spores (>10^5^) in the original undiluted sample to achieve the same minimum detectable threshold established at 10^4^ spores. These data show that the fecal matrix inhibits bioluminescence detection and that reliable detection in fecal samples requires a spore concentration of at least 10^4^.

### NanoLuc Reporter Strain Colonizes and Causes Disease Symptoms as Wild-Type Strain in a Murine Model of CDI

Critical in the development of a genetic tagging system for *in vivo* monitoring is the ability of the harboring strain to retain its infective capabilities. Hence, we tested whether a NanoLuc reporter strain could colonize mice while retaining virulence and remaining capable of producing bioluminescent spores during infection. Spores of wild-type and *193aa-ntd*_*bclA1*_ *-nLuc* strains were used to infect antibiotic-treated mice with a dose of 1 × 10^6^ spores per mouse (Figure 5A–B). As a quality control, we confirmed that infected mice were colonized by the infecting strain using ribotyping and PCR specific to the *nLuc* gene in isolates recovered from stools (Figure S10).

**Figure 5:**
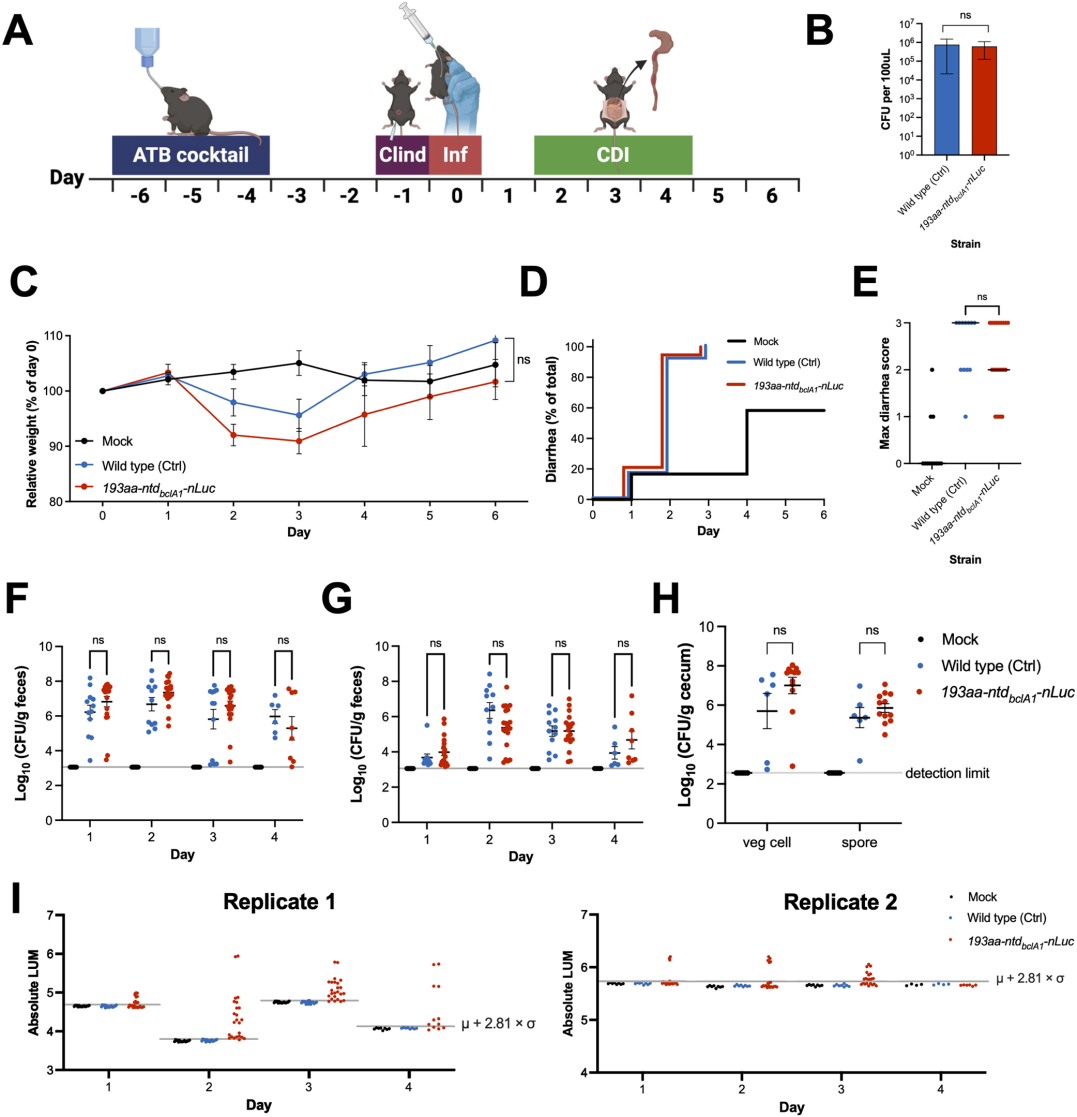
Colonization and disease dynamics in *C. difficile* *193aa-ntd_bclA1_-nLuc*-infected mice. (A) Schematic of experimental design for murine *C. difficile* infection (CDI). Six-week-old C57BL/6J mice were pretreated with an oral antibiotic (ATB) cocktail, followed by intraperitoneal clindamycin administration 1 day prior to infection. Mice were infected via oral gavage with 1 × 10^5^ spores of wild-type (WT; n = 12; 5 males, 7 females), *193aa-ntd_bclA1_-nLuc* *C. difficile* strain (n = 20; equal males and females), or PBS (Mock; n = 12; equal males and females). CDI progression was monitored over 6 days. (B) Quantification of the infectious dose by CFU per gram quantification of inoculum for each group. (C) Percent weight change of mice over the course of infection relative to Day 0. (D) Kaplan–Meier plot showing time to diarrhea onset post-infection. (E) Maximal diarrhea scores recorded during infection. (F) Fecal *C. difficile* vegetative cell load (CFU/g) on days 1–4 post-infection. (G) Fecal spore load (CFU/g) on days 1–4 post-infection. (H) Cecum *C. difficile* vegetative cell and spore load (CFU/g) on Day 3 post-infection. (I) Bioluminescence from undiluted and diluted (1:10) fecal samples collected on days 1–4 post-infection reported as absolute LUM measured at 460 nm in replicates 1 and 2, respectively. Gray lines represent the daily threshold determined as μ + 2.81 × σ. (B–H) Data represent combined results from two independent experiments with indicated sample sizes and sex distributions. Symbols represent individual mice; bars or curves indicate group means ± SEM or cumulative incidence. Statistical analyses were performed using one-way ANOVA with Tukey’s multiple comparisons test. Significance levels are indicated as follows: *P* < 0.05 (*), *P* < 0.01 (**), *P* < 0.001 (***), and *P* < 0.0001 (****).

Clinical disease was similar between groups. At Day 3, wild-type and *nLuc-*tagged strains produced comparable mean weight losses (∼6–7%), while mock controls showed negligible weight change (Figure 5C). Kaplan–Meier analysis showed that 100% of wild-type and *nLuc*-infected mice developed diarrhea by Day 3 post-infection, while only 50% of mock mice developed diarrhea (Figure 5D). Maximal diarrhea scores reached 3 in both infected groups and remained near 0 in mock controls (Figure 5E). Colonization and spore shedding were also indistinguishable between infected groups. Fecal vegetative cell load remained in the 10^6^–10^7^ CFU/g range during the first 2 days for both strains and declined similarly thereafter (Figure 5F). Fecal spore counts over Days 1–4 were similar between wild-type and *nLuc*-infected groups, with burdens generally in the 10^4^–10^6^ CFU/g range and no significant differences between groups (Figure 6G). Cecum vegetative and spore loads at Day 3 were also comparable and in the range of 10^5^–10^7^ CFU/g for both strains, again without significant differences (Figure 5H). Collectively, these results indicate that the presence of *193aa-ntd*_*bclA1*_ *-nLuc* in the *pyrE* loci had no effect on the strains’ ability to colonize and cause disease.

**Figure 6:**
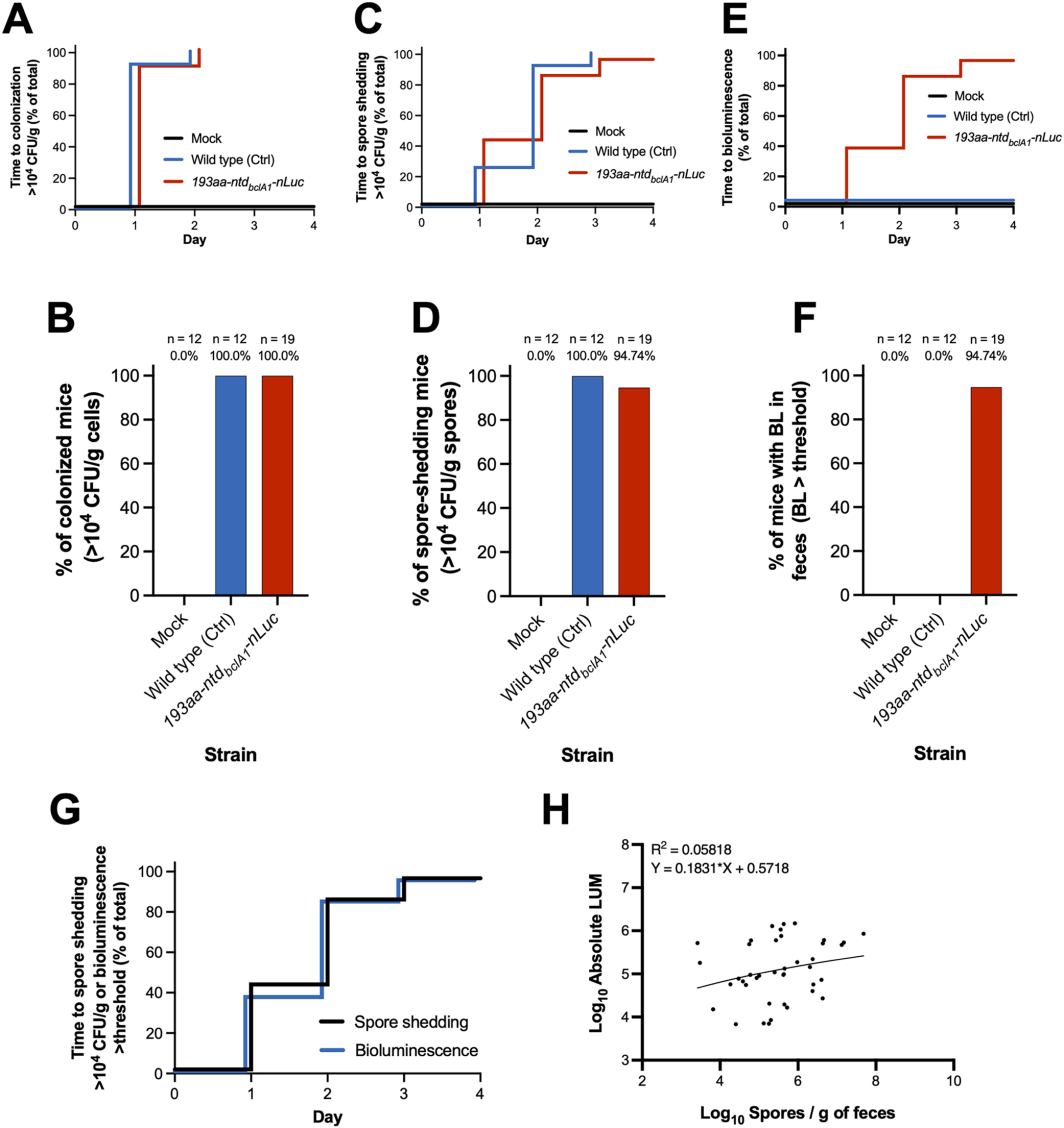
Temporal and quantitative relationship between fecal colonization, spore shedding, and bioluminescence during *C. difficile* infection in murine model. (A) Kaplan–Meier plot showing time to colonization >10^4^ CFU/g. (B) Kaplan–Meier plot showing time to spore shedding >10^4^ CFU/g. (C) Kaplan–Meier plot showing time to bioluminescence above the daily threshold. The daily threshold was determined as μ + 2.81 × σ and calculated using LUM values from mock controls. (D) Percentage of colonized mice with fecal colonization >10^4^ CFU/g cells at any time point post-infection. (E) Percentage of spore-shedding mice with fecal spore load >10^4^ CFU/g spores at any time. (F) Percentage of mice with bioluminescence above the threshold at any time point during CDI. The threshold was calculated as the mean (μ) and standard deviation (σ) of bioluminescence readings in mock animals’ fecal samples and defined bioluminescence positive threshold as μ + 2.81 × σ. (G) Overlap of Kaplan–Meier curves showing the time to spore shedding (>10^4^ CFU/g) and time to detection of bioluminescence (absolute LUM above threshold) in infected mice. (H) Log–log plot comparing spore load (spores/g feces) with corresponding bioluminescence values (absolute LUM, average of replicates) measured in fecal samples. Only data points from samples with bioluminescence above the established detection threshold for both replicates are included. Nonlinear regression analysis was performed; equation and R^2^ value are shown on the plot. Statistical analyses were performed using one-way ANOVA with Tukey’s multiple comparisons test. Significance levels are indicated as follows: *P* < 0.05 (*), *P* < 0.01 (**), *P* < 0.001 (***), and *P* < 0.0001 (****).

Stools were also assayed for bioluminescence to assess whether *C. difficile* *nLuc* strain could form bioluminescent spores. Stool bioluminescence was detectable only in *nLuc*-infected mice, with the bioluminescence signal appearing on Day 1, increasing through Day 3, while remaining at background levels for wild-type-infected and mock mice (Figures 5I and S11, S12, S13N). For classification of positive signals, a daily threshold was defined as the mock group mean plus 2.81 times the standard deviation (μ + 2.81 × σ), which corresponds to the 99.8^th^ percentile of a normal distribution.^79^ Using this threshold, a subset of *nLuc*-infected mice exceeded the threshold at each time point and peaked around 7 × 10^4^ a.u., whereas wild-type and mock groups consistently remained below 4.7 × 10^4^ a.u. (Figures 5I and S11, S12, S13N). Replicate 1 used undiluted feces, yielding robust positives across multiple mice and time points that clearly exceeded the threshold set by control (mock) samples alone. This demonstrated that these controls provide sufficient resolution for positivity determination without any need to dilute samples. In contrast, replicate 2 diluted samples 1:10, resulting in mostly negative signals despite technical triplicates per mouse (often only one positive per positive mouse). Yet, all but one mouse still showed detectable bioluminescence on at least 1 day. Based on prior data establishing a limit of detection at 10^4^ spores (Figure 4B), this dilution effectively requires >10^5^ spores per sample for reliable detection. These findings confirm that even post-dilution, the assay remained sensitive without improving discrimination over undiluted controls, since the dilution unnecessarily suppressed overall detectability (Figure 5I). Overall, the *193aa-ntd*_*bclA1*_ *-nLuc* spore reporter preserves *C. difficile* virulence, colonization, and spore-shedding rates and enables detection of bioluminescence in stools during infection.

### Temporality of Fecal Bioluminescence Correlates with Spore Shedding during CDI

To determine whether fecal bioluminescence could be used as a temporal indicator of spore shedding, we compared the timing and frequency of fecal luminescent signals with culture-based measurements. Kaplan–Meier analysis showed that 90% of wild-type and *nLuc*-infected mice exceeded the colonization threshold (>10^4^ CFU/g feces) by Day 1 post-infection and 100% by Day 2, whereas no mock animal reached this threshold (Figure 6A,B). Similarly, spore shedding >10^4^ CFU/g feces occurred in 100% of wild-type infected mice and ∼95% of *nLuc*-infected mice by Day 3, with no detectable spores in mock controls (Figure 6C, D). Using the previously defined daily bioluminescence threshold, 95% of *nLuc*-infected mice had fecal bioluminescence above the threshold by Day 3, while all wild-type and mock animals remained below the threshold throughout Days 1–4 (Figure 6E,F). Kaplan–Meier curves for spore shedding and bioluminescence in the *nLuc* group closely overlapped, indicating that detection of fecal luminescence closely mirrors the onset of spore shedding *in vivo* (Figure 6G). Finally, we assessed whether fecal bioluminescence correlated quantitatively with spore shedding CFU counts and could be used to quantify spore shedding. We performed linear regression analysis restricted to samples from mice that shed spores at a level >10^4^ CFU/g feces. Upon correlating CFU with bioluminescence, we observed null correlation (R^2^ = 0.058), indicating no quantitative relationship between bioluminescence and spore load. Collectively, these results demonstrate that fecal bioluminescence can predict the presence of shedding spores in the stools during CDI but does not provide a quantitative measure of spores being shed.

### NanoLuc Reporter Does Not Impact *C. difficile* Spore Infectivity or Disease Progression Independent of Host Sex

Women are at higher risk of CDI and recurrent CDI than men,^80^ and female mice develop more severe CDI than males in experimental infection models, in part due to estrous cycle-dependent hormone effects on immune responses.^81^ Host sex can influence infection outcomes;^81^ because of this, we evaluated whether host sex influences reporter strain infectivity or disease progression (Figure S13). Weight loss was similar between sexes, with mean maximal losses in the 5–10% range for both wild-type and *nLuc* groups and negligible change in mock controls (Figure S13A). Kaplan–Meier analysis revealed no significant sex-based differences in diarrhea onset, with incidence reaching 100% in infected groups (Figure S13B). Maximal diarrhea severity was equivalent between sexes, with mean maximal diarrhea scores of 3 for both males and females in wild-type and *nLuc*-infected groups, and no statistically significant differences (Figure S13C). Sex did not measurably affect colonization or spore shedding. Fecal vegetative and spore load in stools over time were similar between males and females within each infection group, with values generally in the 10^4^–10^7^ CFU/g range, and cecal vegetative and spore loads at peak infection also overlapped between ∼10^5^ and 10^7^ CFU/g for both sexes, with no significant sex-specific differences (Figure S13D-F). Kaplan–Meier analysis of colonization, spore shedding, and bioluminescence showed parallel timing for males and females. Both sexes reached the colonization threshold by Day 2, and in *nLuc*-infected mice, ∼85–90% of males and females exceeded the spore-shedding and bioluminescence thresholds by Day 2, while no mock or wild-type mice of either sex surpassed the bioluminescence threshold (Figure S13H–J). In this model, the NanoLuc spore-surface reporter behaves similarly in male and female mice, and host sex does not significantly influence colonization, spore shedding, or reporter detection under these conditions.

### Fluorescent Reporter Spore Tagging Shows Poor Retention in Mature Spores

To expand the utility of the P_*cdeC*_-*ntd*_*bclA1*_ as a spore-surface anchor, the fluorescent reporters *mScarlet-i3* and *mNeonGreen* were fused to either the 48-aa- or 193-aa-*ntd*_*bclA1*_ and subsequently integrated at the *pyrE* locus of R20291_CM210_ or R20291_CM196_ strains (Figures 7A and S14, S15, S16, S17A).^43,82^ The 48-aa- and 193-aa-ntd_*bclA1*_-*mScarlet-i3* strains showed no detectable red signal at stages I–II but displayed fluorescence from stage III onward, peaking in late sporulating cells (Figures 7B and S17B–C). Only a subset of free and purified spores retained red fluorescence, while wild-type exhibited only low background (Figures 7B and S18). In contrast, *mNeonGreen* fusions were indistinguishable from *C. difficile* autofluorescence across all stages (Figure S17B).

**Figure 7:**
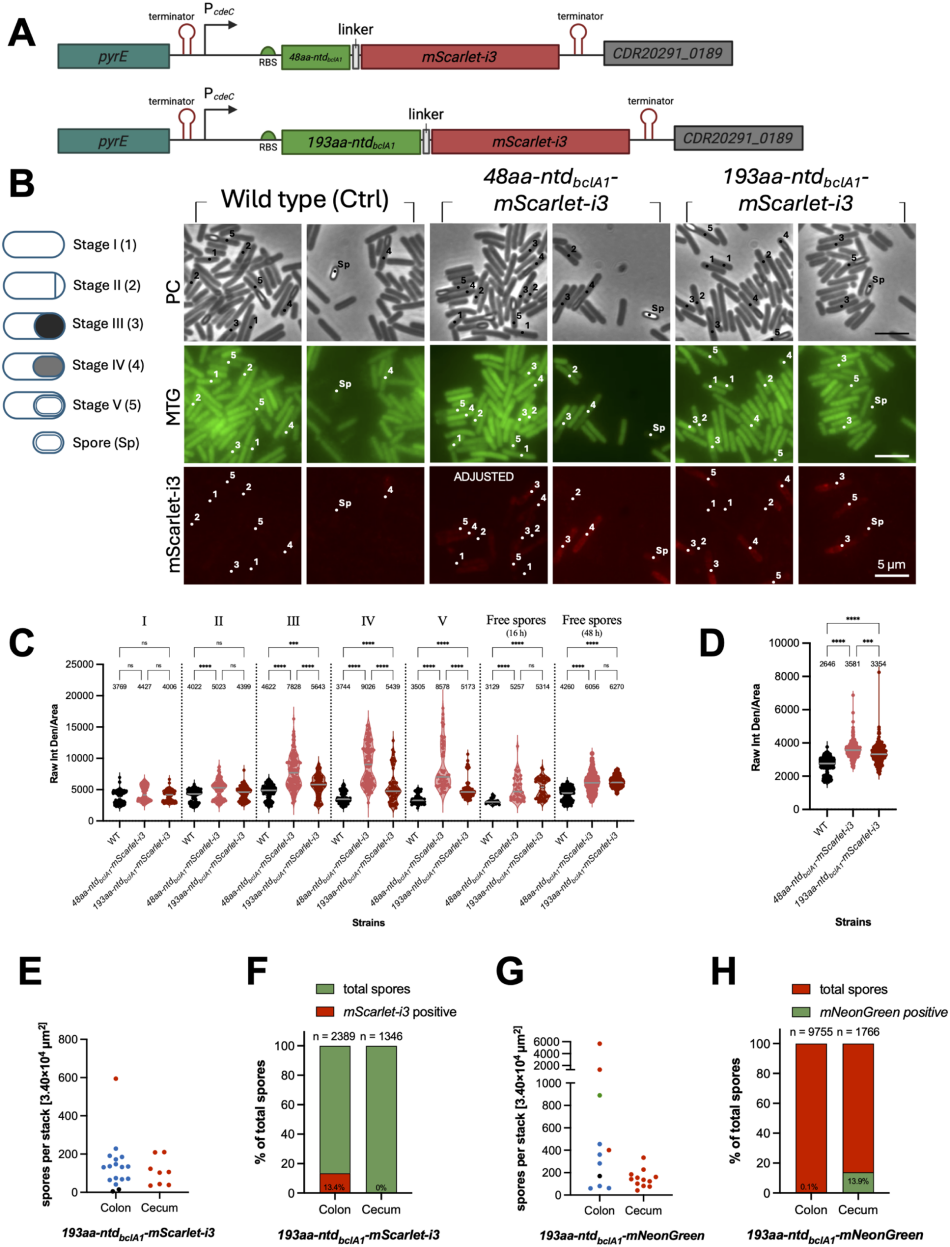
Analysis of*mScarlet*-*i3*expression during *C. difficile* sporulation and*mNeonGreen*and*mScarlet-i3*-tagged *C. difficile* spores formed during infection in a murine model of CDI. (A) Schematic representation of constructs expressing *mScarlet-i3* fused to either a 48-aa or 193-aa *ntd_bclA1_*, driven by the native *cdeC* promoter and inserted at the *pyrE* locus. Each construct includes a ribosome binding site (RBS), linker region, and terminator sequences. (B) Left: schematic of sporulation stages with corresponding stage numbers used for identification. Right: multichannel fluorescence microscopy of wild-type (WT) control, *48aa-ntd_bclA1_-mScarlet-i3*, and *193aa-ntd_bclA1_-mScarlet-i3*. For each strain, panels show phase contrast (PC), membrane stain (MTG, green), and *mScarlet-i3* (red) fluorescence images. Representative images highlighting key sporulation stages (I–V) and mature spores (Sp) as indicated (left). Numbered cells correspond to distinct developmental stages, as diagrammed. (C) Quantification of *mScarlet-i3* fluorescence intensity (raw integrated density per area) as detected by the mCherry/Texas Red filter set across sporulation stages I–V and free spores at 16 and 48 h for each strain. (D) Quantification of *mScarlet-i3* fluorescence intensity in purified spores at 48 h. Data in (C–D) was obtained from two independent biological replicates, with >60 cells analyzed per condition except for spores at 16 h (>20 cells). Each data point represents an individual cell; gray lines indicate medians. Statistical significance was determined by ordinary one-way ANOVA followed by Šídák’s multiple comparisons test. Significance levels are indicated as follows: *P* < 0.05 (*), *P* < 0.01 (**), *P* < 0.001 (***), and *P* < 0.0001 (****). Scale bar: 5 μm. (E, G) Plots showing total spores per field (3.4 × 10^4^ μm^2^) in colon and cecum tissue from mice infected with the *193aa-ntd_bclA1_-mScarlet-i3* or *193aa-ntd_bclA1_-mNeonGreen* strain. Different colors represent data from different animals. (F, H) Proportion of spores within colon and cecum tissues of infected mice with the *193aa-ntd_bclA1_-mScarlet-i3* or *193aa-ntd_bclA1_-mNeonGreen* strain detected by reporter fluorescence. n = 4 mice for the *mNeonGreen* strain and n = 4 mice for the *mScarlet-i3* strain.

Single-cell fluorescence quantification confirmed these observations (Figures 7C–D and S17C–D). Both fusions showed similarly low intensities at stages I–II (∼4,000–5,000 a.u.), but at stage III, the 48-aa construct reached ∼7,800 a.u., significantly higher than the 193-aa fusion (∼5,600 a.u.) (Figure 7C). This ∼40% difference persisted through stages IV–V. Fluorescence decreased by ∼39% in free spores and further in purified spores (∼3,600 vs. ∼3,400 a.u.), with the 48-aa fusion remaining slightly brighter (Figure 7C–D). Wild-type cells maintained low background levels across all stages (Figure 7C–D). Overall, *mScarlet-i3* fluorescence was detectable during late sporulation and in some spores, with higher brightness in the 48-aa fusion but less efficient retention at the spore surface. Intrinsic green autofluorescence in *C. difficile* obscures detection of the *mNeonGreen* reporter (Figure S17C–D). Although the 48-aa and 193-aa fusion constructs showed high single-cell fluorescence (∼10,000–15,000 a.u.) across all developmental stages, these intensities were comparable to or lower than those observed in wild-type cells and spores (Figure S17C–D). Consequently, the *mNeonGreen* signal cannot be reliably distinguished from background fluorescence *in vitro*. Thus, while *mScarlet-i3* localizes to a subset of late-stage sporulating cells and spores, retention is inefficient. Extended analysis is provided in Supplementary Information S2.1.

### Fluorescent Reporters Label Only a Subpopulation of Spores

Since single-cell analysis revealed broad distributions of fluorescence intensities, we re-plotted *mScarlet-i3* data for each fusion strain by developmental stage to gain more insight into fluorescence heterogeneity (Figures S19 and S20). Distributions deviated from normality in many stages (e.g., I, IV–V, spores; Figure S21A–H), indicating distinct subpopulations. Applying a fluorescence threshold as the wild-type mean (μ) plus 2.576 standard deviations (σ), giving a 99.5% confidence interval, to classify the cells and spores into *mScarlet-i3*-positive and -negative cells (Figure S21I–P) confirmed that only a subset of late sporulation cells and spores (>13% in some cases) accumulated detectable signal for both fusions. These analyses suggest a bimodal fluorescence heterogeneity during late sporulation, with released spores displaying weaker but clearly heterogeneous fluorescence confined to a subset of the population. Extended data analysis is provided in Supplementary Information S2.2.

### *In Vivo* Detection of Fluorescently tagged *C. difficile* spores in Colon and Cecum

To assess *in vivo* utility, antibiotic-treated mice were infected with wild-type, *193aantd*_*bclA1*_ *-mNeonGreen*, and *193aantd*_*bclA1*_ *-mScarlet-i3* strains. At the peak of infection (day 3), mice were sacrificed, and cecum and colon tissue were stained for total spores with anti-spore chicken antibody as a control for total spores^11,83^ (Figure S22A). The stains were verified by ribotyping and reporter-specific PCR (Figure S23). For *mScarlet-i3*, ∼13.4% of colonic spores (∼141/3.40 × 10^4^ μm^2^) showed red fluorescence (567 nm excitation, 580–630 nm detection), but none in the cecum (2,389 and 1,346 total spores, respectively; Figure 7E–F). For *mNeonGreen*, no colonic spores fluoresced (488 nm excitation, 500–550 nm detection), but ∼13.9% of cecal spores did (887 and 147 total/3.40 × 10^4^ μm^2^; Figure 7G-H). A noteworthy aspect was that fluorescence-based spore detection *in vivo* seems to be rare and site-dependent. Overall, these results provide insight into how *C. difficile* spores are tagged and imaged *in vivo*, highlighting substantial limitations and improvements of both reporters for visualizing *C. difficile* spores *in vivo*. Extended *in vivo* analysis is provided in Supplementary Information S2.3.

In the mother cell, its localization and retention on the spore surface was less efficient.

## Discussions

*C. difficile* is a major cause of healthcare-associated infections because its spores persist in the environment and mediate transmission between hosts.^9,84,85^ Current methods for detecting and quantifying *C. difficile* spores include bacteriological culturing, qPCR, and microscopy;^11,14–18^ although they offer temporal and anatomical resolution, they do not recognize *C. difficile* spores formed solely during CDI rather than those present in the infective dose. This restricts the ability to monitor newly formed spores *in situ* during infection. Although recent bioluminescent and fluorescent reporters allow single-cell studies for *C. difficile*,^42,43,86,87^ they have not been adapted to track spore formation within the gastrointestinal tract. In this work, we developed the first spore-specific tagging system for *C. difficile* using the BclA1 N-terminal domain to anchor reporters to the spore surface. The NanoLuc-based system detects spore shedding above ∼10^4^ CFU/g in stools but does not provide a quantitative correlation between CFU and luminescence. Moreover, fluorescent reporters followed bimodal expression and low tagging efficiency, revealing underappreciated challenges for future optimization. Moreover, tissue-specific expression/tagging was also observed, exposing major challenges for tagging *C. difficile* spores formed during an infection.

Unlike prior systems that express luciferase intracellularly in metabolically active bacteria or rely on self-luminescent *lux* operons, this work introduces a modular BclA1 N-terminal tag that localizes NanoLuc on the *C. difficile* spore surface. To our knowledge, luciferase has not previously been displayed on the *C. difficile* or other Gram-positive bacteria spore surfaces for *in vivo* detection. The design uses the sporulation-specific P_*cdeC*_, in place of the weak native P_*bclA1*_, to drive NanoLuc expression and the *bclA1* N-terminal as a spore surface anchor. Because the exosporium forms the outermost spore layer, surface-displayed NanoLuc is directly accessible to excitation and substrate without germination or permeabilization, making it more suitable for detecting intact spores shed during infection than reporters buried in the coat, cortex, or core, where protective layers can limit substrate access and attenuate signal. In R20291 strains, *bclA1* is pseudogenized and produces a truncated N-terminal fragment that still localizes to the spore surface,^51,62^ which motivated our use of N-terminal BclA1 fusions rather than full-length constructs. Two N-terminal variants were tested in parallel: a truncated 48-aa form produced by the nonsense mutation in R20291 and a repaired 193-aa variant. We did not test full-length BclA1-reporter fusions in a *bclA1* mutant background or quantify endogenous BclA1 levels in the NanoLuc strain relative to the parental strain, as suitable anti-BclA1 antibodies for specific detection in sporulating cultures are currently not available. Future work will require the development of BclA1-directed reagents to determine labeling efficiency and to assess how reporter fusions affect native BclA1 expression across the spore population. In the tested variants, the 193-aa tag produced higher bioluminescence and localized to the spore coat/exosporium without altering spore ultrastructure or spore infectivity in the murine model. The luminescent signal from tagged spores increased in proportion to NanoLuc substrate *in vitro*, with maximal outputs achieved at a 1:100 dilution and reliable detection down to 1:100,000. However, unlike *lux* operons that produce their own substrate,^88^ NanoLuc requires exogenous furimazine,^49^ so spatial and temporal variability in substrate delivery and pharmacokinetics must be considered as additional constraints for *in vivo* detection. A further limitation for the detection of bacteria in stools is matrix interference, a well-documented limitation for bioluminescence assays.^77,78^ In fecal matrices, the effective dynamic range narrowed to ∼10^4^–10^6^ spores, consistent with quenching and light scattering by stool. In the murine model, fecal bioluminescence was detected only at ≥10^4^ spores per gram and correlated with spore counts (R^2^ ≈ 0.06), whereas *lux*-tagged *E. coli* and recombinant lactic acid bacteria typically achieve reliable fecal detection near 10^5^–10^6^ CFU per 100 mg and near-linear photon-CFU relationships with R^2^ ≥ 0.94–0.98 across several logs.^29,78,89^ These differences likely reflect limited enzyme load per spore, fecal quenching, and scattering. In previously reported work, bioluminescent proteins are expressed throughout metabolically active bacterial cells or *lux* operons, which produce their own luciferin, supporting proportional quantitative readouts.^29,78,89,90^ By contrast, our NanoLuc is anchored to the exosporium of dormant spores and depends entirely on externally supplied furimazine.^49^ As a result, our Nanoluc spore tag could be best interpreted as a qualitative marker that detects spore shedding ≥10^4^ spores per gram rather than a tool optimized for precise CFU quantification. Future work will be needed to apply whole-body and organ-focused bioluminescence imaging to define the *in situ* sensitivity, anatomical distribution, and temporal dynamics of NanoLuc-tagged spores along the gastrointestinal tract, and to determine how closely tissue photon flux correlates with local spore burden.

Attempts were made to achieve inducible, temporally restricted *nLuc* expression so that only spores formed after a specific trigger (e.g., antibiotic exposure or recurrence) would be labeled. Despite successful surface tagging of *C. difficile* spores, *tetR*-based regulation did not yield luminescence levels comparable to those of unregulated tags. The TetR system fully repressed bioluminescence in the absence of an inducer, but activation with anhydrotetracycline (aTc) remained weak relative to native promoters, even after introducing *tetO* sites into both the *tetR* and *nLuc* promoters to tune repressor levels and operator occupancy. Although TetR/aTc systems provide tight repression and tunable induction in other organisms,^71,91^ the growth conditions required for *C. difficile* may contribute to the limited induction observed here, as aTc degrades rapidly at 37 °C.^92^ In addition, aTc stability, penetration, and the timing of TetR production relative to sporulation-specific promoters collectively limit inducibility. It remains possible that placing *tetR* under an alternative promoter, either weaker or differently timed with respect to sporulation, could improve repressor levels and allow induction. However, these limitations suggest that future work should focus on alternative regulatory strategies that have already shown tight control in bacteria, including *C. difficile*.^93–99^ Xylose-inducible systems could provide dose-responsive induction using an inexpensive, gut-relevant sugar to control NanoLuc synthesis with xylose administration.^93,100^ Cumate-inducible promoters, which have been used in *L. monocytogenes*,^94^ could offer an additional option for expression control that is independent of antibiotic analogs, potentially avoiding the uptake and stability problems that limit aTc-based systems under anaerobic conditions.^95,96^ Beyond transcriptional control, post-transcriptional regulation (for example, inducible riboswitches or toehold switches placed upstream of *nLuc*) could be used to gate translation only in the presence of a small-molecule cue.^97–99^ In these systems, *nLuc* mRNA can be transcribed from a sporulation promoter but would remain translationally silent until binding of an inducer restructures the 5′ UTR to expose the ribosome-binding site, or until a trigger RNA unlocks a toehold switch. This would allow NanoLuc synthesis to be restricted to the presence of a small molecule without relying solely on promoter strength or TetR levels.^97–99^ These strategies could allow for inducible expression of spore tagging system, while avoiding aTc stability and induction limitations that constrained the TetR system in this work. In practice, we envision a next generation of constructs that combine a robust sporulation-specific promoter (such as P_*cdeC*_) with an orthogonal inducer (e.g., xylose or cumate) or RNA-based switch to restrict NanoLuc synthesis to defined windows of infection or treatment.

Additional attempts for fluorescent spore tagging resulted in reporter expression during *in vitro* sporulation but provided unreliable labeling of *C. difficile* spores formed *in vivo*. Previously, in a dual *mNeonGreen*/*mScarlet-i3* transcriptional reporter system under P_*cwp2*_ control, both fluorophores produced signals in defined vegetative subpopulations using widefield LED excitation optimized for cytosolic imaging (470 nm for mNeonGreen*/*515/40 nm emission; 550 nm for mScarlet-i3/595/40 nm emission), confirming their folding and maturation compatibility in *C. difficile*.^43,101^ Here, by contrast, mScarlet-i3 was detected in sporulating cells *in vitro,* but fluorescence was consistently lost upon release of mature spores for both truncated (48-aa) and repaired (193-aa) N-terminal variants, suggesting a defect in localization, retention, or maturation of the fluorophores on the spore surface. While it is unclear why results using the NanoLuc construct are not consistent with those using fluorescent proteins, it is possible that mScarlet-i3 localization to the spore surface is impacted by other exosporium proteins such as BclAs, CdeC, and CdeM, which can cause steric constraints hindering their incorporation. It is also plausible that the larger size of mScarlet-i3 (∼25.8 kDa), compared to NanoLuc (∼19 kDa), may limit its anchoring to the spore surface.^18,43,63,82^ In addition, the exosporium context may exacerbate folding and processing problems if mScarlet-i3 maturation is slower than the window between synthesis of the fusion and completion of coat/exosporium assembly. Prior exosporium proteomics show processing and shedding of BclA-family proteins and raise the possibility that mScarlet-i3 fusions are proteolyzed or released during spore maturation rather than stably retained on the surface.^102,103^ Consistent with exosporium heterogeneity, only a subset of spores displayed detectable red fluorescence, in line with the coexistence of thick and thin exosporium morphotypes and likely differences in exosporium assembly between these forms.^51,104^ Additionally, reporter expression followed the timing of P_*cdeC*_ activation at late stages of sporulation, suggesting that both fluorescent and NanoLuc tags likely label only a subpopulation of spores rather than the entire spore population. Other groups have used mNeonGreen as a transcriptional reporter in *C. difficile* but already reported substantial green autofluorescence; the data here confirm that limitation and extend it to spore-surface tags and to *in vivo* intestinal tissues.^43,57,105^ In infected mice, reliable fluorescent detection was confined to a subset of colon regions for mScarlet-i3 and to cecal regions for mNeonGreen, likely reflecting combined limits of reporter expression and stability during *in vivo* spore formation, particularly as our confocal parameters (488 nm/500–550 nm for mNeonGreen; 567 nm/580–630 nm for mScarlet-i3) prioritized narrow *in vivo* detection windows over the broader LED/filter sets used for validation and in previous studies, potentially reducing sensitivity for low-level spore surface signals. Despite the limited fluorescence observed with mScarlet-i3 and mNeonGreen, NanoLuc-tagged spores produced a broad, quantifiable dynamic range in fecal bioluminescence assays and enabled reliable detection of spore shedding at ≥10^4^ spores per gram, supporting efficient spore tagging. However, the fraction of spores that express *nLu*c *in situ* across different intestinal regions remains undefined. We attempted immunofluorescence using anti-NanoLuc antibodies to localize the tag on the spore surface, but the antibody failed to recognize conformational epitopes under our conditions, precluding accurate single-spore localization. Future work combining *in vivo* bioluminescence imaging with optimized antibody-based or alternative labeling strategies will be required to determine whether surface-displayed luciferase provides superior tissue-resolved information compared to fluorescent protein tags. Overall, these observations indicate that the future use of mScarlet-i3 and mNeonGreen will require significant optimization, particularly during spore assembly-tagging, to be useful for *in vivo* spore tracking. Improving anaerobic optimization of fluorescent reporters or autofluorescence may help overcome some of the hurdles observed in this work.

## Supplementary Material













## Notes

The following supporting information is available free of charge at the ACS website. . The Supporting Information is available free of charge at https://pubs.acs.org/doi/10.1021/acssynbio.5c00961.

Figure S1. Deletion of *pyrE* fragment in R20291_CM196_ *C. difficile* strain; Figure S2. Restoration of *pyrE* fragment in R20291_CM196\_ *ΔpyrE* *C. difficile* strain; Figure S3. Promoter regulation and phenotypic characterization of bioluminescent spore-tagged strains in *C. difficile;* Figure S4. Confirmation of insertion of *nLuc* in spore-tagged mutants by PCR; Figure S5. Growth curves of *C. difficile*NanoLuc spore tagged mutants compared to the R20291 Δ*pyrE*/*pyrE*+ control strain; Figure S6. Confirmation of insertion of *nLuc* and growth curves of spore tagged mutants; Figure S7. Transmission electron microscopy of NanoLuc-tagged and wild-type *C. difficile* spores; Figure S8. Raw images of western blots and SDS-PAGE gel of purified *C. difficile* spores; Figure S9. Raw data of bioluminescence readings for dynamic range analysis of NanoLuc-tagged *C. difficile* spores in fecal samples; Figure S10. Ribotype PCR and PCR detection of infectious strain identity in mouse isolates; Figure S11. Raw data of bioluminescence readings of fecal samples during CDI from Day 1–4 post-infection (replicate 1); Figure S12. Raw data of bioluminescence readings of fecal samples during CDI from Day 1–4 post-infection (replicate 2); Figure S13. Sex-stratified analysis of NanoLuc-tagged *C. difficile* strain dynamics in a murine CDI model; Figure S14. Growth curves of *C. difficile* *mScarlet-i3* and *mNeonGreen* spore-tagged mutants compared to R20291 *ΔpyrE/pyrE*+ control strain; Figure S15. PCR detection of *mScarlet-i3* and *mNeonGreen* and growth curves of spore-tagged mutants; Figure S16. *mScarlet-i3* expression during *C. difficile* sporulation; Figure S17. Analysis of *mNeonGreen* expression during *C. difficile* sporulation cycle; Figure S18. Representative images of *mScarlet-i3* expression in free spores (48 h) and purified *C. difficile* spores; Figure S19. Heterogeneity of *mScarlet-i3* expression during *C. difficile* sporulation cycle; Figure S20. Heterogeneity of *mScarlet-i3* expression during the *C. difficile* sporulation cycle in free spores (48 h) and purified spores; Figure S21. Normality and per-cell fluorescence distributions across sporulation stages in *mScarlet-i3*-tagged strains; Figure S22. Confocal imaging of *mNeonGreen* and *mScarlet-i3*-tagged *C. difficile* spores formed during infection in a murine model of CDI; and Figure S23. Ribotype PCR and PCR detection of infectious strain identity in mouse isolates (PDF)
Table of contents (PDF)
S1: Extended Materials and Methods: Bacterial strains and growth conditions; Construction of CM196 ΔpyrE C. difficile strain; Plasmid construction; Construction of C. difficile reporter strains; C. difficile growth curves; Sporulation efficiency using phase-contrast microscopy; Purification of C. difficile spores; Bioluminescence assays with purified spores; Nano-Glo reagent titration; Dynamic range of bioluminescence of spores in fecal samples; Transmission Electron Microscopy; Western blotting; Murine model of CDI; Quantification of C. difficile vegetative cells and spores from feces and cecum; Bioluminescence from feces and cecum; PCR ribotyping and confirmation of nLuc fragment in stool-derived strains; Fluorescent Microscopy of fluorescent spore surface reporter strains; Imaging of fluorescent spore surface reporter strains during CDI; Tissue Fixation, Immunofluorescence Staining and Confocal Imaging; PCR ribotyping and confirmation of mScarlet-i3 or mNeonGreen fragment in stool-derived strains; Statistical Analysis and S2: Fluorescent reporter data analysis: mScarlet-i3 is expressed by sporulating cells but poorly retained in mature spores; mScarlet-i3 tagging is restricted to a subpopulation of C. difficile cells; and In vivo detection of fluorescently tagged C. difficile spores in colon and cecum (PDF)
Tables S1. Bacterial strains used (PDF)
Table S2. Plasmids used (PDF)
Table S3. Primers used (PDF)
