# Supplementary material for: Development and Evaluation of Exosporium-Anchored Bioluminescent and Fluorescent Reporters for Tracking Clostridioides difficile Spores Formed In Vivo
Source: ACS Synth Biol. 2026 May 15;15(6):2338–55. doi: 10.1021/acssynbio.5c00961 (PMC13288923; doi:10.1021/acssynbio.5c00961)
Supplement: Supplementary file 1 [file sb5c00961_si_001.pdf]

Development and evaluation of exosporium-anchored bioluminescent and fluorescent reporters for tracking *Clostridioides difficile* spores produced *in vivo*.

Osiris K. Lopez-Garcia[1,2], Trey Hejtmancik[1], Marjorie Pizarro-Guajardo[1], Lindsey Brehm[1], Christian Brito-Silva[3], and Daniel G. Paredes-Sabja[1,2,4*]

[1]Department of Biology, Texas A&M University, College Station, Texas, U.S.A.

[2]Interdisciplinary Graduate Program in Genetics & Genomics, Texas A&M University, College Station, Texas, U.S.A.

[3]ANID – Millennium Science Initiative Program – Millennium Nucleus in the Biology of the Intestinal Microbiota, Santiago, Chile.

[4]Department of Biology, Texas A&M University, College Station, Texas, U.S.A, Email: dparedes-sabja@tamu.edu

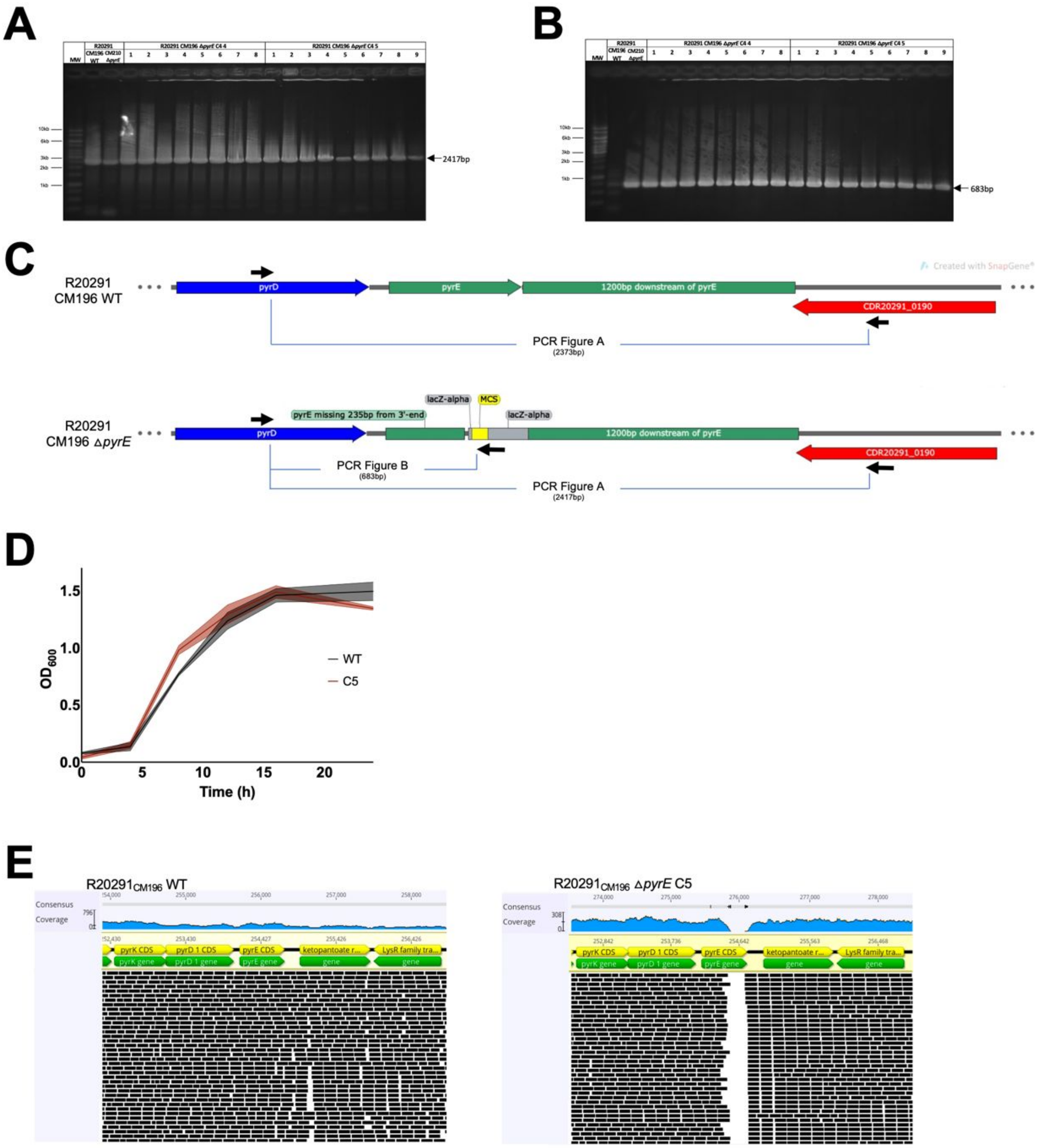

F

SNP Analysis of R20291CM196 ΔpyrE constructs against R20291 GCA_015732555.1_ASM1573255 genome									
Variant						R20291 CM196 WT		R20291 CM196 ΔpyrE C5	
Locus Tag	CDS	Protein ID	Product	Change	Amino Acid Change	Position	Variant Frequency	CDS Position	Variant Frequency
CDIF27147_00009	rsbV CDS	QPK94785.1	anti-sigma-B factor antagonist	C -> T	L -> F	9186	100.00%	9186	100.00%
CDIF27147_00010	rsbW CDS	QPK94786.1	serine-protein kinase	G -> T	G -> V	9694	100.00%	9694	100.00%
CDIF27147_01523	putative CDS	QPK96158.1	putative secreted protein	T -> C	D -> G	1605292	100.00%	1605292	100.00%
CDIF27147_02036	vncR CDS	QPK96652.1	two-component system response regulator	A -> G	D -> G	2134234	98.90%	2134234	99.40%
CDIF27147_02733	putative CDS	QPK97326.1	two-component sensor histidine kinase	(T)7 -> (T)6		2895029	92.60%	2895029	96.60%
	intergenic			(A)5 -> (A)6		685627	91.70%	685827	97.50%
CDIF27147_00429	rbsK CDS	QPK95094.1	ribokinase	(A)5 -> (A)4		371626	89.70%	371626	93.60%
	intergenic			(A)12 -> (A)13		3859939	83.00%	3859939	91.00%
CDIF27147_01523	putative CDS	QPK96158.1	putative secreted protein	C -> A	D -> Y	1605284	64.90%	1605284	73.70%
CDIF27147_01523	putative CDS	QPK96158.1	putative secreted protein	CAA -> TAG		1605279	50.0% -> 64.3%		
CDIF27147_00311	pyrE CDS	QPK94978.1	orotate phosphoribosyltransferase	T -> C				254271	99.50%
CDIF27147_00311	pyrE CDS	QPK94978.1	orotate phosphoribosyltransferase	A -> C					
CDIF27147_00311	pyrE CDS	QPK94978.1	orotate phosphoribosyltransferase	AA -> GT	K -> V			254509	94.3% -> 97.1%
CDIF27147_00311	pyrE CDS	QPK94978.1	orotate phosphoribosyltransferase	ACT -> GCC	T -> A			254503	70.6% -> 75.0%
CDIF27147_00311	pyrE CDS	QPK94978.1	orotate phosphoribosyltransferase	G -> C	G -> A				
CDIF27147_00311	pyrE CDS	QPK94978.1	orotate phosphoribosyltransferase	ACT -> GCG	T -> A			254500	61.0% -> 70.6%
CDIF27147_01523	putative CDS	QPK96158.1	putative secreted protein	A -> T	L -> M				
	intergenic			A -> C				2544736	77.80%
	intergenic			+C				1602932	70.80%
	intergenic			+CC				1602932	70.80%
CDIF27147_01523	putative CDS	QPK96158.1	putative secreted protein	CA -> AG	L -> S			1605287	60.0% -> 85.7%
CDIF27147_00311	pyrE CDS	QPK94978.1	orotate phosphoribosyltransferase	TT -> AA					
	intergenic			A -> GC					

Figure S1. Deletion of *pyrE* fragment in R20291$_{CM196}$ *C. difficile* strain. (A) Detection of *pyrE* loci by PCR amplification of *C. difficile* R20291$_{CM196}$ wild type and Δ*pyrE* mutant strains using primers that anneal to the upstream and downstream genes, *pyrD* and CDR20291_0190 respectively. Expected size for mutant strains is 2417bp. (B) Detection of *lacZ-alpha* fragment

insertion by PCR amplification of *C. difficile* $R20291_{CM196}$ wild type and Δ*pyrE* mutant strains using primers that anneal to upstream gene *pyrD* and to inserted *lacZ-alpha* fragment. No amplification is expected for the wild type strain and a 683bp fragment is expected for mutant strains. (C) Schematic map of *C. difficile* $R20291_{CM196}$ *pyrE* loci in wild type and Δ*pyrE* mutant strains. Black arrows represent binding sites of primers. (D) Effect of deletion of *pyrE* fragment on growth of mutant strains. Growth curves of three technical replicates of *C. difficile* $R20291_{CM196}$ wild type and Δ*pyrE* mutant strain grown in Brain Heart Infusion medium for 24 h. *C. difficile* CM196 R20291 wild type strain was used as control, shown in black. Shown are the OD_{600} measurements on the y-axis, error bands show the range of values of the three replicate measurements. (E) Genomic DNA from wildtype and mutant was sequenced by Illumina with mapped read depth of minimum 400Mbp. Paired end reads were mapped to *C. difficile* R20291 GCA_015732555.1_ASM1573255 reference genome using Bowtie2 with default parameters. Shown is the coverage of the reads in the *pyrE* loci. Alignments of mutant C5 resulted in no alignment of reads to the last 235bp of *pyrE.* (F) Single nucleotide polymorphisms detection and identification in the genomic sequences assembled to *C. difficile* R20291 GCA_015732555.1_ASM1573255 reference genome.

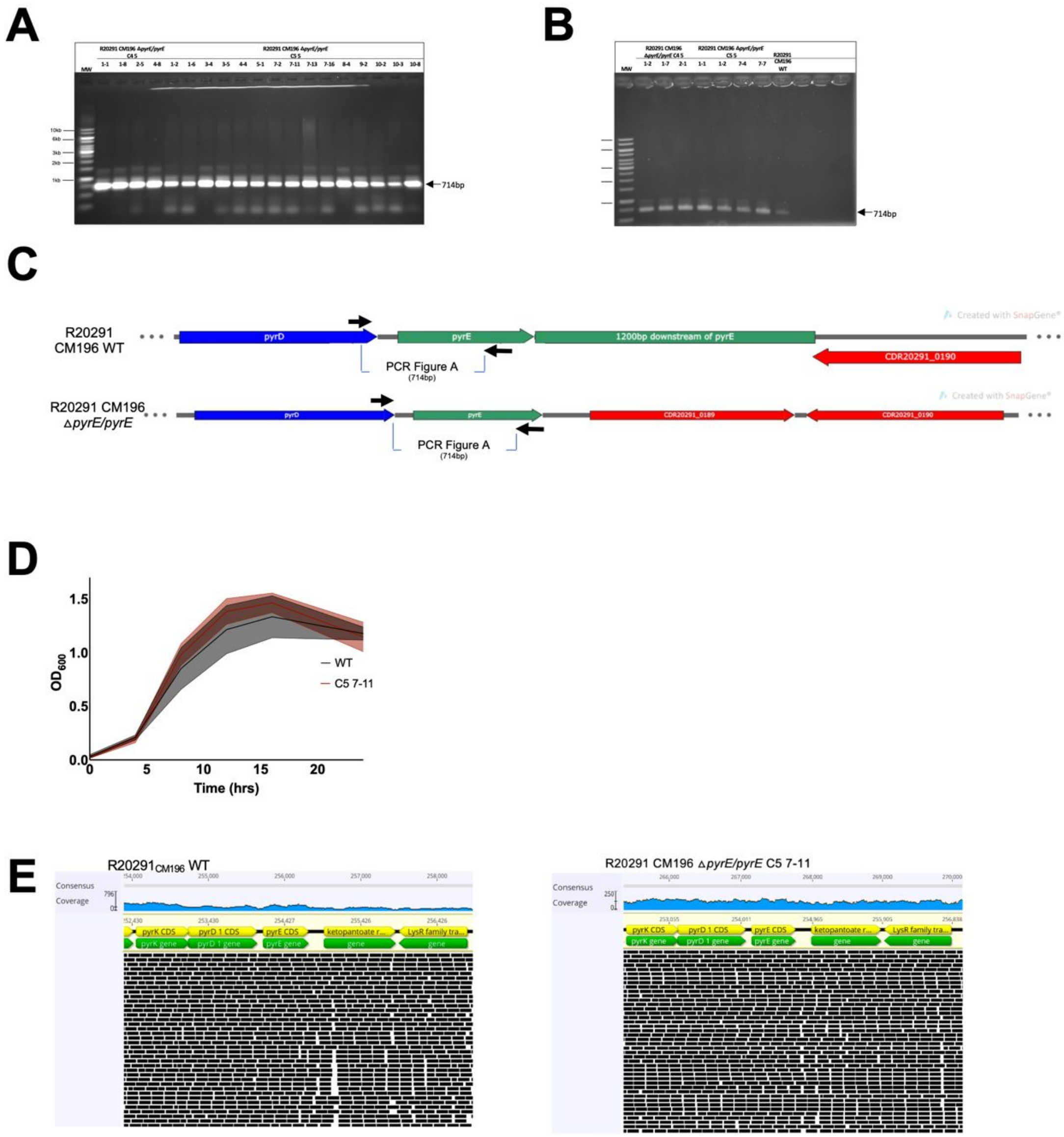

F

SNP Analysis of *C. difficile* R20291$_{CM196}$ Δ*pyrE/pyrE* mutants assembled to *C. difficile* R20291 GCA_015732555.1_ASM1573255 reference genome								
Variant					R20291 CM196 WT		R20291 CM196 Δ*pyrE/pyrE* C5 7-11	
CDS	Protein ID	Product	Change	Amino Acid Change	Genomic Position	Variant Frequency (%)	Genomic Position	Variant Frequency (%)
rsbW	QPK94786.1	serine-protein kinase	G -> T	G -> V	9694	100.00	9694	100.00
vncR	QPK96652.1	two-component system response regulator	A -> G	D -> G	2134234	99.30	2134234	100.00
rsbV	QPK94785.1	anti-sigma-B factor antagonist	C -> T	L -> F	9186	99.20	9186	100.00
putative	QPK96158.1	putative secreted protein	T -> C	D -> G	1605292	96.20	1605292	100.00
rbsK	QPK95094.1	ribokinase	(A)5 -> (A)4		371626	95.20	371626	90.40
intergenic			(A)5 -> (A)6		685627	93.10	685627	93.10
putative	QPK97326.1	two-component sensor histidine kinase	(T)7 -> (T)6		2895029	92.80	2895029	96.80
intergenic			(A)12 -> (A)13		3859939	90.90	3859939	85.60
pyrE	QPK94978.1	orotate phosphoribosyltransferase	T -> C					
putative	QPK96158.1	putative secreted protein	T -> A					
intergenic			A -> G					
intergenic			T -> A					
intergenic			TT -> AA					
putative	QPK96158.1	putative secreted protein	C -> A	D -> Y			1605284	83.30

Figure S2. Restoration of *pyrE* fragment in R20291$_{CM196}$ Δ*pyrE C. difficile* strain. (A-B) Detection of fully restored *pyrE* by PCR amplification of *C. difficile* R20291$_{CM196}$ wild type and Δ*pyrE* mutant strains using primers that anneal to upstream gene *pyrD* and to *pyrE* gene

fragment. A 714bp fragment is expected for wild type and mutant strains. (C) Schematic map of *C. difficile* R20291$_{CM196}$ *pyrE* loci in wild type and Δ*pyrE* mutant strains. Black arrows represent binding sites of primers. (D) Effect of restoration of *pyrE* fragment on growth of mutant strains. Growth curves of three technical replicates of *C. difficile* R20291$_{CM196}$ wild type and mutant strain grown in Brain Heart Infusion medium for 24 h. *C. difficile* R20291$_{CM196}$ wildtype strain was used as control, shown in black. Shown are the OD_{600} measurements on the y-axis, error bands show the range of values of the three replicate measurements. (E) Genomic DNA from wildtype and mutant was sequenced by Illumina with mapped read depth of minimum 400Mbp. Paired end reads were mapped to *C. difficile* R20291 GCA_015732555.1_ASM1573255 reference genome using Bowtie2 with default parameters. Shown is the coverage of the reads in the *pyrE* loci. Alignments of mutant C5 7-11 resulted in full alignment of reads to the full length of *pyrE.* (F) Single nucleotide polymorphisms detection and identification in the genomic sequences assembled to *C. difficile* R20291 GCA_015732555.1_ASM1573255 reference genome.

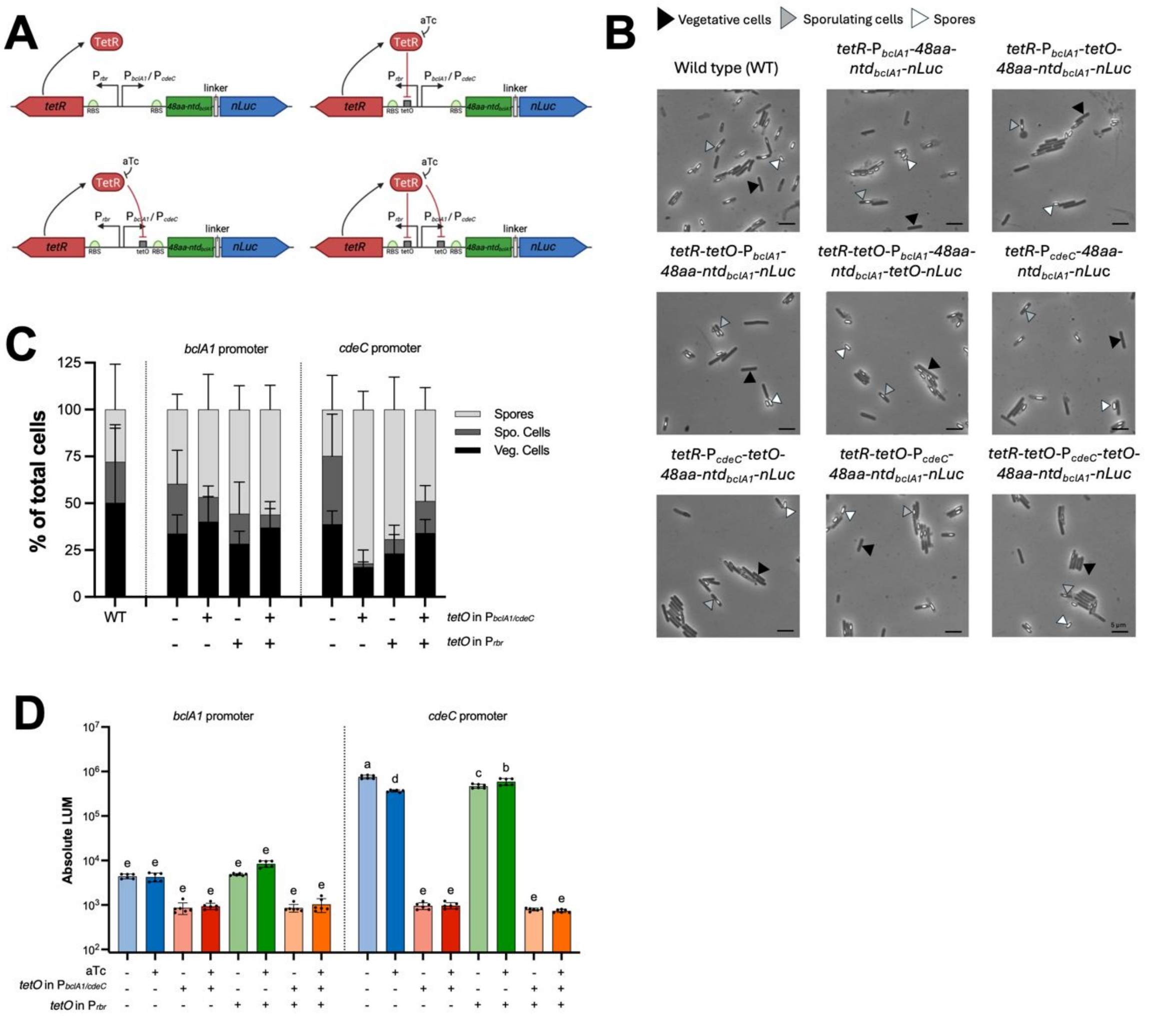

Figure S3. Promoter regulation and phenotypic characterization of bioluminescent spore-tagged strains in *C. difficile*. (A) Schematic representation of inducible expression systems for NanoLuc-tagged constructs driven by either *bclA1* or *cdeC* promoter, with or without regulation by the tetracycline-inducible repressor (*tetR*) in either the *rubrerythrin* promoter, *bclA1*/*cdeC* promoter or both. (B) Representative phase-contrast micrographs of sporulating cultures after 16 h of incubation in sporulation medium. (C) Sporulation efficiency of wild-type and NanoLuc-tagged strains after 16 h of incubation in sporulation medium, determined by quantification of vegetative cells, sporulating cells and spores in phase-contrast images. Sporulation efficiency is reported as the percentage of each cell type relative to the total cells counted. Data reported as means ± SD from three independent replicates, with >300 cells quantified per strain in each replicate. (D) Bioluminescence quantification of purified *C. difficile* spores expressing *nLuc* constructs under either *bclA1* or *cdeC* promoter, in the presence or absence of the inducer anhydrotetracycline (aTc). Luminescence values (absolute luminescence units, LUM) presented in Log_{10} scale as means ± SD from two independent biological replicates, each measured in technical triplicate. Statistical significance was determined by ordinary one-way ANOVA,

followed by Šídák's multiple comparisons test. Groups sharing the same letter are not significantly different, while different letters indicate significant differences ($P < 0.05$).

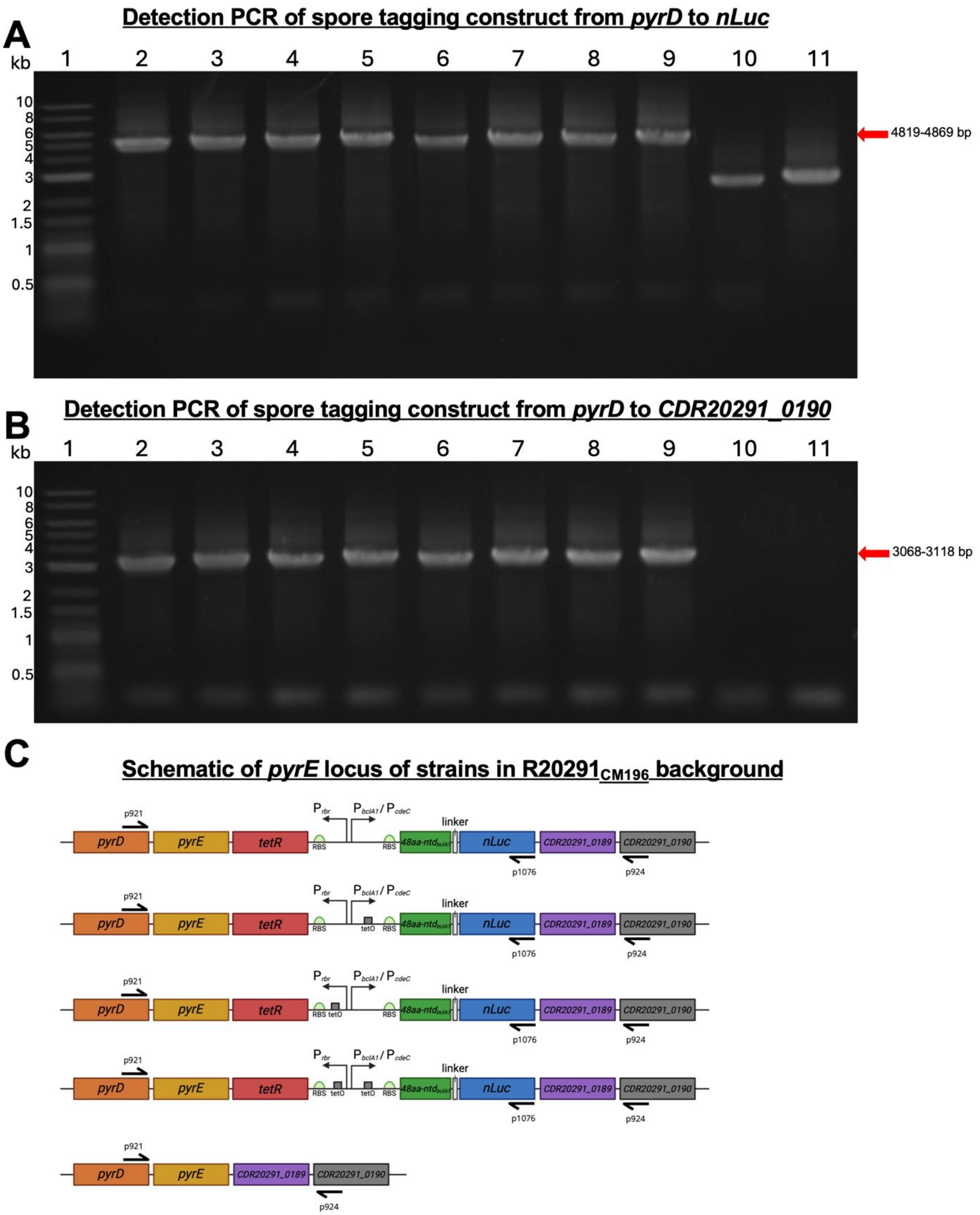

Figure S4. Confirmation of insertion of *nLuc* in spore tagged mutants by PCR. (A) Agarose gel electrophoresis of PCR products amplified from genomic DNA using primers that anneal to the upstream and downstream genes flanking the *pyrE* locus. Lane 1: ZR 1kb DNA marker (Zymo) (kb); Lane 2: R20291$_{CM196}$ Δ*pyrE*/*pyrE*+ *tetR*-P$_{rbr}$-P$_{bclA1}$-*tetO*-*48aa*-*ntd*$_{bclA1}$-*nLuc*; Lane

3: R20291$_{CM196}$ Δ*pyrE*/*pyrE*+ *tetR*-P$_{rbr}$-*tetO*-P$_{bclA1}$-*tetO-48aa-ntd*$_{bclA1}$-*nLuc*; Lane 4: R20291$_{CM196}$ Δ*pyrE*/*pyrE*+ *tetR*-P$_{rbr}$-P$_{cdeC}$-*tetO-48aa-ntd*$_{bclA1}$-*nLuc*; Lane 5: R20291$_{CM196}$ Δ*pyrE*/*pyrE*+ *tetR*-P$_{rbr}$-*tetO*-P$_{cdeC}$-*tetO-48aa-ntd*$_{bclA1}$-*nLuc*; Lane 6: R20291$_{CM196}$ Δ*pyrE*/*pyrE*+ *tetR*-P$_{rbr}$-P$_{bclA1}$-*48aa-ntd*$_{bclA1}$-*nLuc*; Lane 7: R20291$_{CM196}$ Δ*pyrE*/*pyrE*+ *tetR*-P$_{rbr}$-P$_{cdeC}$-*48aa-ntd*$_{bclA1}$-*nLuc*; Lane 8: R20291$_{CM196}$ Δ*pyrE*/*pyrE*+ *tetR*-P$_{rbr}$-*tetO*-P$_{bclA1}$-*48aa-ntd*$_{bclA1}$-*nLuc*; Lane 9: R20291$_{CM196}$ Δ*pyrE*/*pyrE*+ *tetR*-P$_{rbr}$-*tetO*-P$_{cdeC}$-*48aa-ntd*$_{bclA1}$-*nLuc*; Lane 10: R20291$_{CM196}$ WT; Lane 11: R20291$_{CM196}$ Δ*pyrE.* Mutant strains, each showing a band of ~4819-4869 bp, which is substantially larger than the wild-type control (lane 10; 2373 bp) and the Δ*pyrE* deletion control (lane 11; 2465 bp). The presence of the higher molecular weight band in mutant lanes, relative to controls, confirms correct integration at the *pyrE* locus. (B) PCR confirmation of *nLuc* reporter gene insertion, using a forward primer annealing outside *pyrE* loci and a reverse primer binding within *nLuc*. Lane numbering is as in (A). All mutant strains (lanes 2-9) yield a product of ~3068-3118 bp, confirming *nLuc* insertion. No amplification is observed in the wild-type or Δ*pyrE* controls (lanes 10-11). (C) Schematic of *pyrE* locus of strains in R20291$_{CM196}$ background with locations of primers used for PCR detection.

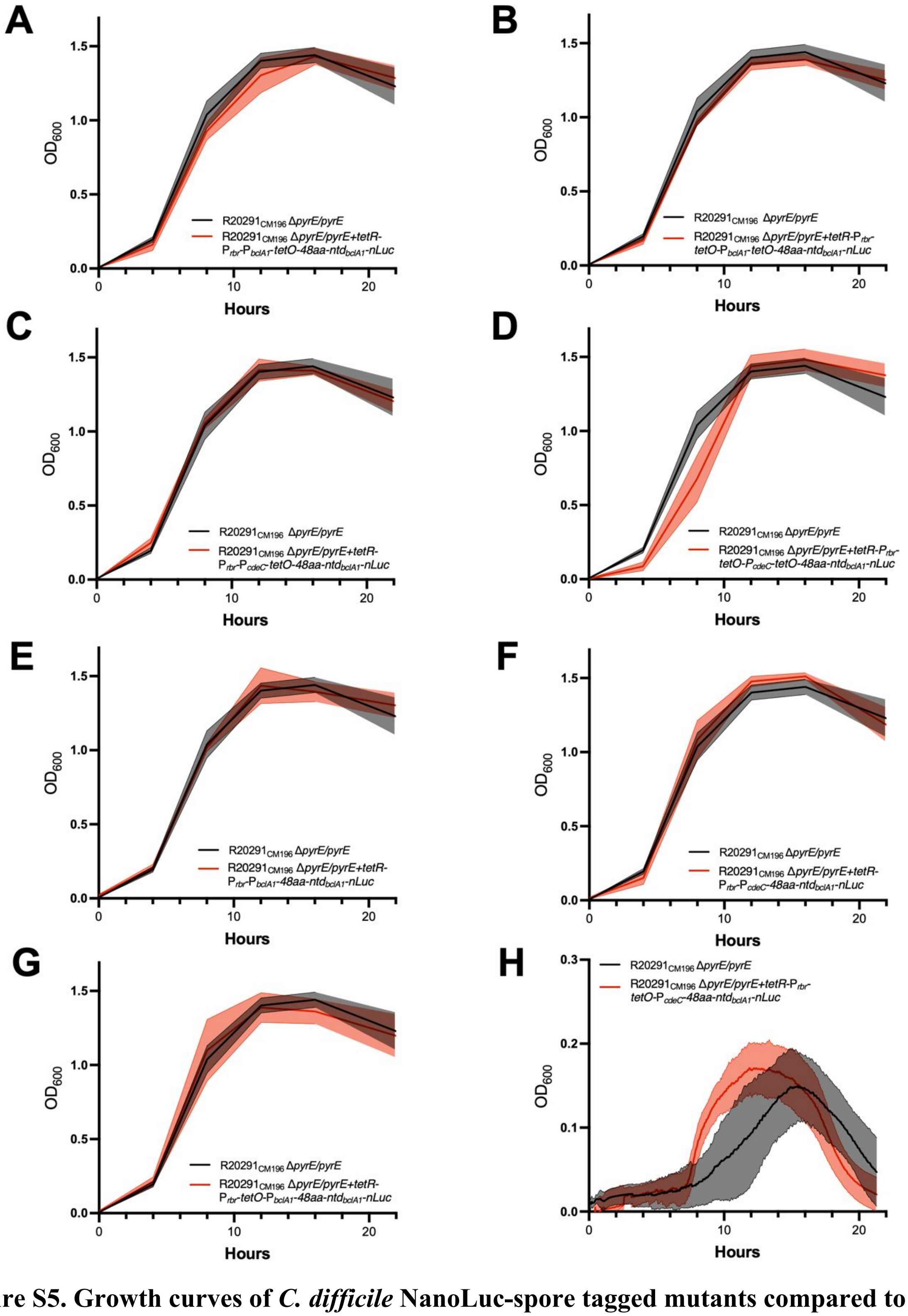

Figure S5. Growth curves of *C. difficile* NanoLuc-spore tagged mutants compared to the R20291 Δ*pyrE*/*pyrE*+ control strain. Panels (A-H) display the growth curves of mutant strains cultured anaerobically, with OD600 measured over time. The strains shown in panels (A) through (H) are: (A) R20291$_{CM196}$ Δ*pyrE/pyrE*+ *tetR*-P$_{rbr}$-P$_{bclA1}$-*tetO-48aa-ntd*$_{bclA1}$-*nLuc*; (B)

R20291$_{\mathrm{CM196}}$ Δ*pyrE/pyrE*+ *tetR*-P$_{rbr}$-*tetO*-P$_{bclA1}$-*tetO-48aa-ntd*$_{bclA1}$-*nLuc*; (C) R20291$_{\mathrm{CM196}}$ Δ*pyrE/pyrE*+ *tetR*-P$_{rbr}$-P$_{cdeC}$-*tetO-48aa-ntd*$_{bclA1}$-*nLuc*; (D) R20291$_{\mathrm{CM196}}$ Δ*pyrE/pyrE*+ *tetR*-P$_{rbr}$-*tetO*-P$_{cdeC}$-*tetO-48aa-ntd*$_{bclA1}$-*nLuc*; (E) R20291$_{\mathrm{CM196}}$ Δ*pyrE/pyrE*+ *tetR*-P$_{rbr}$-P$_{bclA1}$-*48aa-ntd*$_{bclA1}$-*nLuc*; (F) R20291$_{\mathrm{CM196}}$ Δ*pyrE/pyrE*+ *tetR*-P$_{rbr}$-P$_{cdeC}$-*48aa-ntd*$_{bclA1}$-*nLuc*; (G) R20291$_{\mathrm{CM196}}$ Δ*pyrE/pyrE*+ *tetR*-P$_{rbr}$-*tetO*-P$_{bclA1}$-*48aa-ntd*$_{bclA1}$-*nLuc*; and
(H) R20291$_{\mathrm{CM196}}$ Δ*pyrE/pyrE*+ *tetR*-P$_{rbr}$-*tetO*-P$_{cdeC}$-*48aa-ntd-bclA1-nLuc*. The R20291$_{\mathrm{CM196}}$ Δ*pyrE/py*rE control strain growth is included as a black line in each panel for comparison. Data represent the mean of three independent biological replicates, with standard deviation indicated as shaded error bands.

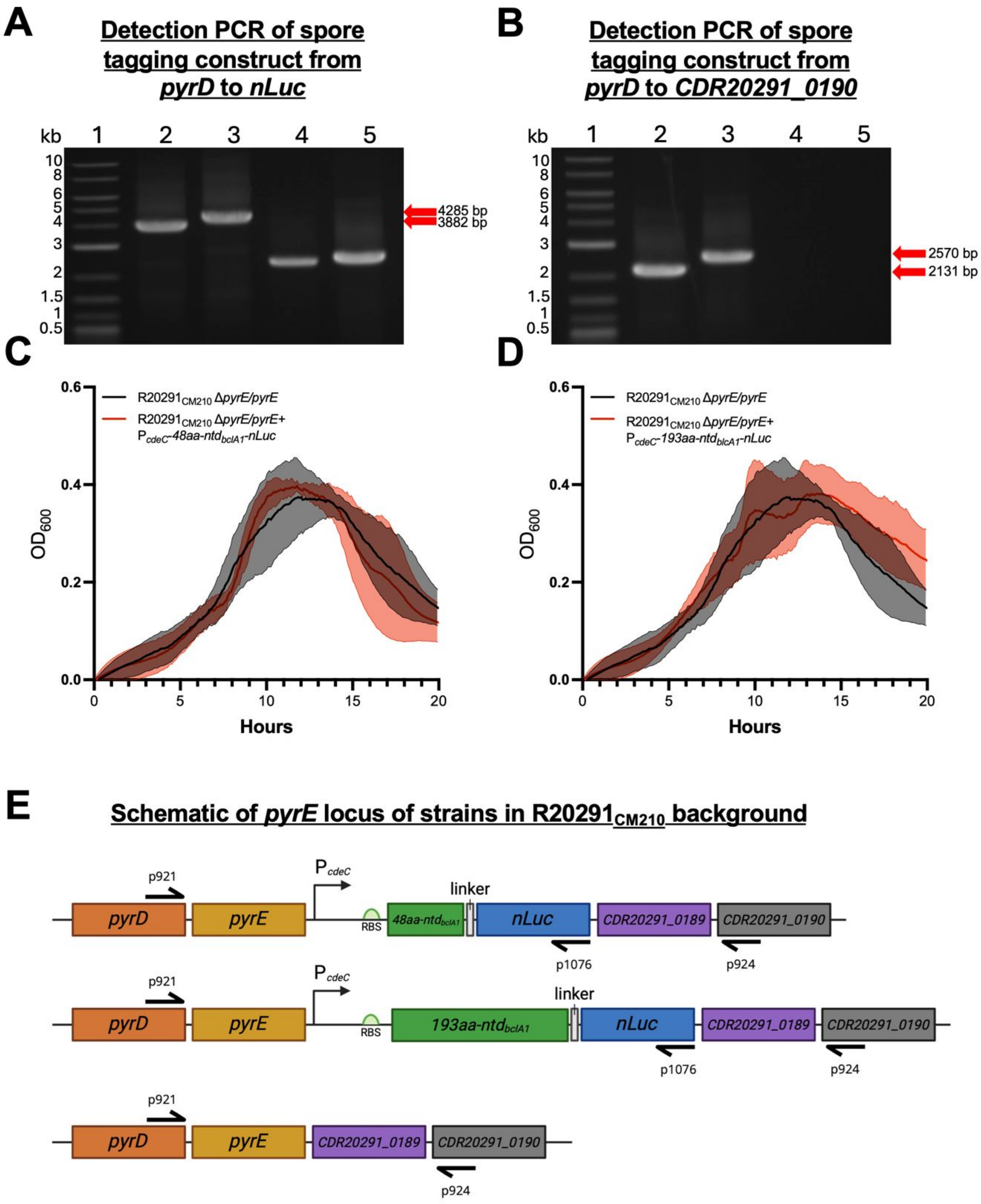

Figure S6. Confirmation of insertion of *nLuc* and growth curves of spore tagged mutants. (A) Agarose gel electrophoresis of PCR products amplified from genomic DNA using primers that anneal to the upstream and downstream genes flanking the *pyrE* locus. Lane 1: ZR 1kb DNA marker (Zymo) (kb); Lane 2: R20291$_{CM210}$ Δ*pyrE*/*pyrE*+ P$_{cdeC}$-*48aa-ntd$_{bclA1}$-nLuc*; Lane 3: R20291$_{CM210}$ Δ*pyrE*/*pyrE*+ P$_{cdeC}$-*193aa-ntd$_{bclA1}$-nLuc*; Lane 4: R20291$_{CM210}$ WT; Lane 5: R20291$_{CM210}$ Δ*pyrE.* Mutant strains, each showing a band of 3882 and 4285 bp, which is larger than expected band wild-type control (lane 4; 2373 bp) and the Δ*pyrE* control (lane 5; 2465 bp).

The presence of the higher molecular weight band in mutant lanes, relative to controls, confirms correct insertion of constructs at the *pyrE* locus. (B) PCR confirmation of *nLuc* reporter gene insertion, using a forward primer annealing outside *pyrE* loci and a reverse primer binding within *nLuc*. Lane numbering is as in (A). All mutant strains (lanes 2-3) yield a product of 2131 and 2570 bp, confirming *nLuc* insertion. No amplification is observed in the wild-type or Δ*pyrE* controls (lanes 4-5). Panels (C-D) display the growth curves of mutant strains cultured anaerobically, with OD_{600} measured over time. The strains shown are: (C) $R20291_{CM210}$ Δ*pyrE/pyrE*+ P_{cdeC}-*48aa-ntd*$_{bclA1}$-*nLuc*; and (D) $R20291_{CM210}$ Δ*pyrE/pyrE*+ P_{cdeC}-*193aa-ntd*$_{bclA1}$-*nLuc*. The $R20291_{CM210}$ Δ*pyrE/py*rE control strain growth is included as a black line in each panel for comparison. Data represent the mean of three independent biological replicates, with standard deviation indicated as shaded error bands. (E) Schematic of *pyrE* locus of strains in $R20291_{CM210}$ background with locations of primers used for PCR detection.

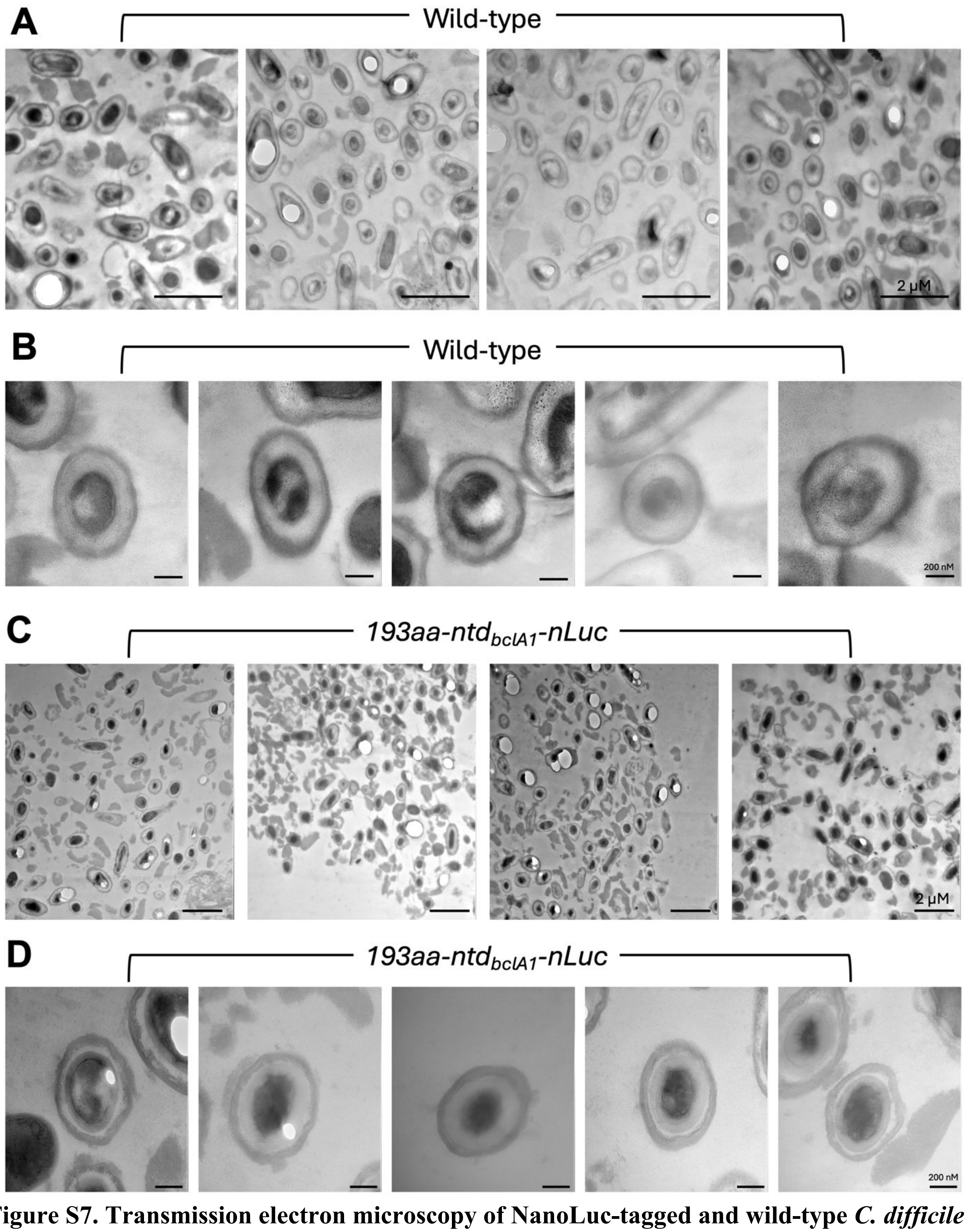

Figure S7. Transmission electron microscopy of NanoLuc-tagged and wild-type *C. difficile* spores. (A) Representative TEM images of purified wild-type spores; scale bars represent 2 µm. (B) Higher magnification TEM images of individual wild-type spores illustrating spore and exosporium morphology; scale bars represent 200 nm. (C) Representative TEM images of purified *193aa-ntd*$_{bclA1}$*-nLuc* spores acquired under the same imaging conditions as in panel A;

scale bars represent 2 μm. (D) Higher magnification TEM images of individual *193aa-ntd*$_{bclA1}$-*nLuc* spores showing spore and exosporium morphology; scale bars represent 200 nm.

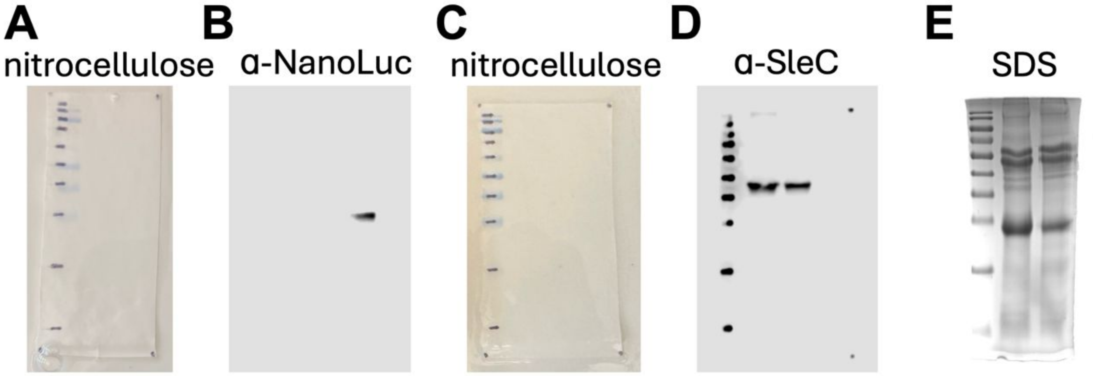

Figure S8. Raw images of western blots and SDS-PAGE gel of purified *C. difficile* spores. (A) Image of membrane used for western blot with anti-NanoLuc antibody. (B) Western blot imagining of spore coat and exosporium extracts from purified wild type WT and *193aa-ntd*$_{bclA1}$-*nLuc* spores using antibody against NanoLuc (C) Image of membrane used for western blot with anti-SleC antibody. (D) Western blot imaging of spore coat and exosporium extracts from purified WT and *193aa-ntd*$_{bclA1}$-*nLuc* spores with antibody against SleC. (E) Uncropped Coomassie-stained gel is shown as an additional loading control.

PBS
Undiluted fecal samples
1:10 fecal samples
1:50 fecal samples

A

	1	2	3	4	5	6	7	8	9	10	11	12
A	WT 10^6		WT 10^1		WT 10^6		WT 10^1		WT 10^6		WT 10^1	
B	NLuc 10^6		NLuc 10^5		NLuc 10^4		NLuc 10^3		NLuc 10^2		NLuc 10^1	
C	WT 10^6		WT 10^1									
D	NLuc 10^6		NLuc 10^5		NLuc 10^4		NLuc 10^3		NLuc 10^2		NLuc 10^1	
E												
F	NLuc 10^6		NLuc 10^5		NLuc 10^4		NLuc 10^3		NLuc 10^2		NLuc 10^1	
G												
H	NLuc 10^6		NLuc 10^5		NLuc 10^4		NLuc 10^3		NLuc 10^2		NLuc 10^1	

B

	1	2	3	4	5	6	7	8	9	10	11	12
A	31377	30507	27574	25869	25170	24485	25346	25327	25818	27447	27707	25403
B	OVRFLW	OVRFLW	354654	359591	64020	62734	29624	28692	22788	22356	21461	23782
C	31971	33440	28851	28865	29375	27979	29263	30314	30446	29511	28482	28005
D	930097	907686	86532	86323	32734	33176	29311	27924	24993	26468	27384	28130
E	30123	31963	30637	28950	30077	28503	28927	27297	29014	29469	28378	28654
F	246014	239086	40606	41529	30045	29199	27767	25893	27167	27507	26233	25566
G	44702	47539	33355	29764	27522	27695	28130	27157	26632	27497	27581	26664
H	OVRFLW	OVRFLW	770982	758320	114958	113303	35239	36793	27638	25595	25185	26397

C

	1	2	3	4	5	6	7	8	9	10	11	12
A	26166	26987	23251	23592	19579	21199	19788	20358	22450	22018	19890	20747
B	3739696	3771342	310337	291087	45917	44966	21901	21636	19223	20625	19865	19114
C	27319	27652	25006	24403	25051	23005	24650	22715	24816	24329	22668	22349
D	933589	938250	98679	94599	28778	31141	23872	23557	22572	22914	21272	21095
E	25077	26444	23942	25011	23922	24207	23030	23866	23247	22843	23175	22201
F	235560	224144	38288	42175	24603	23449	22793	22689	21100	21105	21273	20908
G	43147	41891	27482	25363	24032	23987	22917	24195	24127	21563	22490	19790
H	OVRFLW	OVRFLW	1156011	1139894	111179	109399	28441	29971	21248	20898	20121	20017

D

	1	2	3	4	5	6	7	8	9	10	11	12
A	27774	27497	27388	25926	21317	22872	21563	21788	26146	25303	24235	24455
B	2366678	2420057	106003	103369	32857	32558	23217	23968	22534	24746	20752	22571
C	28103	28403	26684	26412	26901	28732	26512	28282	26228	26708	26239	27231
D	592303	599779	49758	46457	26668	29117	25425	27420	23092	25982	27110	25481
E	29517	28566	29597	28296	28167	27783	27670	26453	26881	25643	27245	25238
F	162452	149159	31735	29536	27629	26824	26661	26178	25624	26115	24701	24480
G	31813	32152	30123	27363	25418	27739	26251	26292	25794	25929	26131	25538
H	3847401	4045354	335507	363974	72082	68586	29211	29230	26487	25449	24351	24252

Figure S9. Raw data of bioluminescence readings for dynamic range analysis of NanoLuc-tagged *C. difficile* spores in fecal samples. (A) 96-well plate layout with distribution of samples per treatment in duplicate. Blue colored wells were used for samples using PBS, purple for samples using undiluted feces, red for samples using 1:10 dilution of feces samples and green for samples using 1:50 dilution of feces samples. (B-D) Replicate bioluminescence readings recorded at 460 nm from three independent assays.

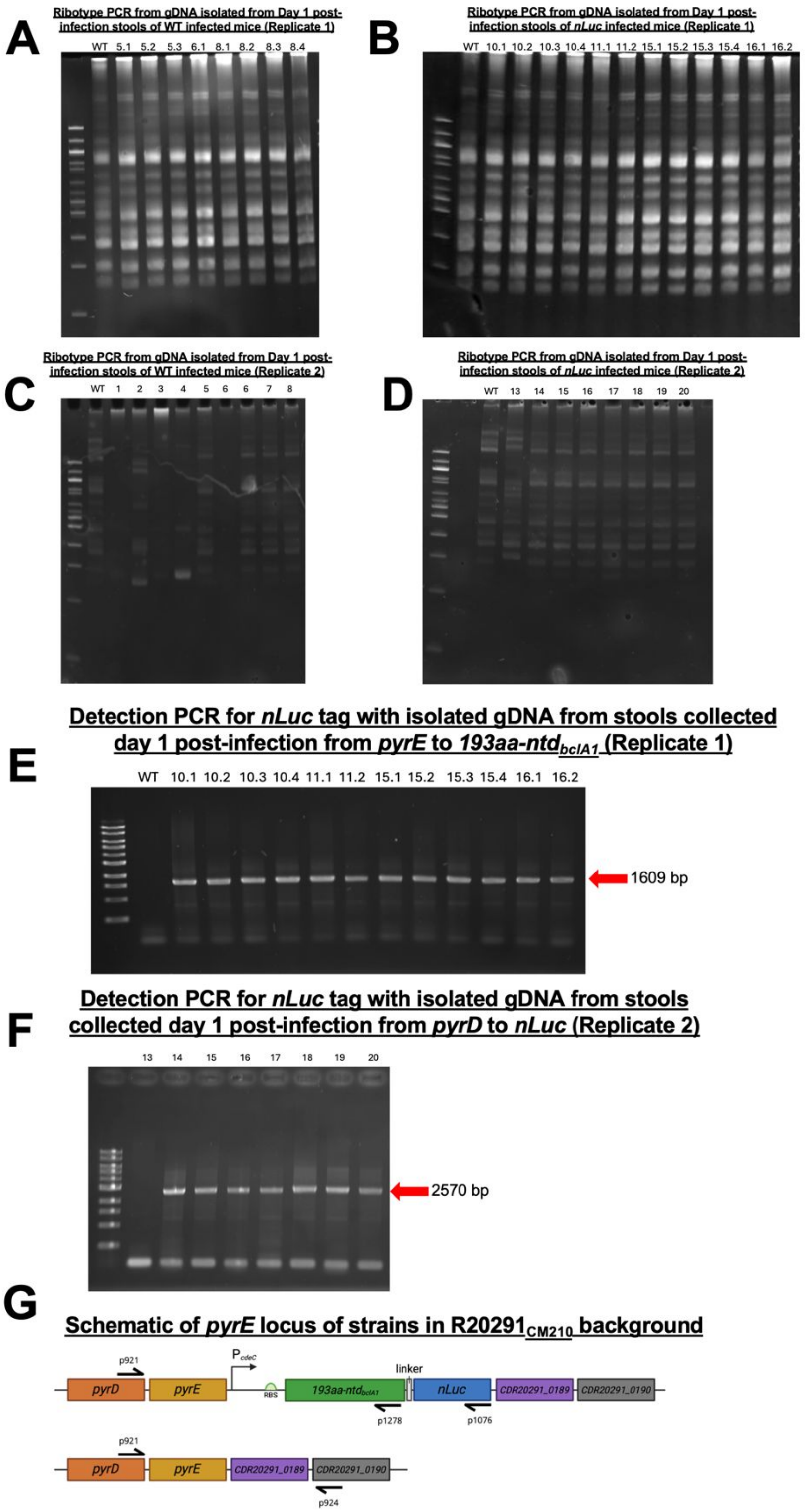

Figure S10. Ribotype PCR and PCR detection of infectious strain identity in mouse isolates. (A-D) Ribotype PCR profiles of isolates from fecal samples Day 1 post-infection.

Bands correspond to amplification of 16S and 23S rRNA regions. Lanes are grouped by mouse number and compared with wild-type (WT) control, to confirm if mice were infected with the correct ribotype matching the input R20291$_{CM210}$ infectious strain. A, wild type infected mice (5.1-6.4) from first replicate; B, *193aa-ntd*$_{bclA1}$*-nLuc* infected mice (10.1-16.2) from first replicate; C, Mock PBS infected mice (1-4) and wild type infected mice (5-8) from second replicate; D, *193aa-ntd*$_{bclA1}$*-nLuc* infected mice (13-20) from second replicate. (E) PCR amplification from *pyrE* to *193aa-ntd*$_{bclA1}$. Genomic DNA from isolates in *193aa-ntd*$_{bclA1}$*-nLuc* infected mice was screened for the presence of the *193aa-ntd*$_{bclA1}$*-nLuc*-specific DNA fragment (1609 bp), with wild type as negative control. *193aa-ntd*$_{bclA1}$*-nLuc* infected mice (10.1-16.2) from first replicate. (E) PCR amplification from *pyrD* to *nLuc.* Genomic DNA from isolates in *193aa-ntd*$_{bclA1}$*-nLuc* infected mice was screened for the presence of the *193aa-ntd*$_{bclA1}$*-nLuc*-specific DNA fragment (2570 bp), with wild type as negative control. *193aa-ntd*$_{bclA1}$*-nLuc* infected mice (13-20) from second replicate. (G) Schematic of *pyrE* locus of strains in R20291$_{CM210}$ background with locations of primers used for PCR detection.

A

	1	2	3	4	5	6	7	8	9	10	11	12
A	1.1	1.1	1.2	1.2	1.3	1.3	1.4	1.4	3.1	3.1	3.2	3.2
B	3.3	3.3	3.4	3.4	5.1	5.1	5.2	5.2	5.3	5.3	6.1	6.1
C									8.1	8.1	8.2	8.2
D	8.3	8.3	8.4	8.4								
E	10.1	10.1	10.2	10.2	10.3	10.3	10.4	10.4	11.1	11.1	11.2	11.2
F												
G	15.1	15.1	15.2	15.2	15.3	15.3	15.4	15.4	16.1	16.1	16.2	16.2
H												

B

	1	2	3	4	5	6	7	8	9	10	11	12
A	46809	46654	43361	46327	44611	43087	45470	43969	45728	45220	46878	44098
B	42688	43952	43023	45629	43080	42886	48094	47929	46954	44914	45662	48059
C									44358	46110	44580	41807
D	40230	42110	42397	41552								
E	45696	46300	41678	40939	40898	41763	47546	46068	43039	42155	76810	79239
F												
G	42207	44247	94490	86785	99136	97506	41260	42194	56236	59096	54969	55709
H												

C

	1	2	3	4	5	6	7	8	9	10	11	12
A	5903	5885	5429	5686	5311	5837	5481	5385	5621	5393	5754	5409
B	5257	5385	5807	6093	5985	5940	6144	5778	5513	5830	6264	5744
C									5343	5268	6022	5569
D	5598	5508	6047	6087								
E	16410	16881	19618	19506	7162	7114	26190	28170	58138	55728	39779	39941
F												
G	8074	8986	6470	6061	6703	7410	6636	7014	70851	74570	834266	865089

D

	1	2	3	4	5	6	7	8	9	10	11	12
A	1.1	1.1	1.2	1.2	1.3	1.3	1.4	1.4	3.1	3.1	3.2	3.2
B	3.3	3.3	3.4	3.4	5.1	5.1	5.2	5.2	5.3	5.3	6.1	6.1
C												
D			10.1	10.1	10.2	10.2	10.3	10.3	10.4	10.4	11.1	11.1
E	11.2	11.2										
F			15.1	15.1	15.2	15.2	15.3	15.3	15.4	15.4	16.1	16.1
G	16.2	16.2										
H												

E

	1	2	3	4	5	6	7	8	9	10	11	12
A	55477	56488	53476	54779	60181	59412	54257	53554	60309	56764	54475	58093
B	54365	60187	55249	55895	61977	62254	56668	54204	59770	56446	51731	50003
C												
D			58736	59437	186032	173774	188199	185216	209226	229882	614363	597728
E	134261	133301										
F			62081	60402	79511	81807	91718	98403	66110	68529	114893	104233
G	95144	95241										
H												

F

	1	2	3	4	5	6	7	8	9	10	11	12
A	1.3	1.3	1.4	1.4	3.3	3.3	3.4	3.4	5.2	5.2	5.3	5.3
B							10.3	10.3	10.4	10.4	11.1	11.1
C							15.2	15.2	15.4	15.4	16.2	16.2
D												
E												
F												
G												
H												

G

	1	2	3	4	5	6	7	8	9	10	11	12
A	12153	11973	11161	10384	12011	11396	12320	11665	12366	11998	12529	11954
B							15299	15099	19648	21175	530315	543791
C							11536	10815	12390	12777	144627	146131
D												
E												
F												
G												
H												

Figure S11. Raw data of bioluminescence readings of fecal samples during CDI from Day 1-4 post-infection replicate 1. (A) 96-well plate layout with distribution of samples for Day 1 and 2. (B) Bioluminescence readings recorded at 460 nm from Day 1 post-infection fecal samples. (C) Bioluminescence readings recorded at 460 nm from Day 2 post-infection fecal

samples. (D) 96-well plate layout with distribution of samples for Day 3. (E) Bioluminescence readings recorded at 460 nm from Day 3 post-infection fecal samples. (F) 96-well plate layout with distribution of samples for Day 4. (G) Bioluminescence readings recorded at 460 nm from Day 4 post-infection fecal samples.

A

	1	2	3	4	5	6	7	8	9	10	11	12
A	1		2		3		4		5		6	
B	7		8									
C												
D	13			14			15			16		
E												
F	17			18			19			20		
G												
H												

B

	1	2	3	4	5	6	7	8	9	10	11	12
A	49079	47434	48813	48719	50878	47073	51551	50079	45797	48889	49796	51264
B	46898	49818	49957									
C												
D	47905	53621	46371	50447	49409	49821	51105	50265	54536	47211	48259	52940
E												
F	49055	48106	50832	147987	159024	138725	50684	47251	47660	50289	49851	49526
G												
H												

C

	1	2	3	4	5	6	7	8	9	10	11	12
A	42799	42033	39474	43390	44455	45014	43076	42948	43642	44219	45834	42463
B	43851	47229	44105	45254								
C												
D	45149	44331	45877	127754	123408	135577	50768	50114	51823	44002	44460	42261
E												
F	41200	43367	44684	41621	42806	42835	124707	147735	158889	44758	43520	41675
G												
H												

D

	1	2	3	4	5	6	7	8	9	10	11	12
A	45153	46754	45998	45402	42317	45598	44331	47052	44658	43234	45347	45438
B	42408	47228	44144	49484								
C												
D	44684	44489	44391	79919	71681	74779	49933	46302	44472	48132	44202	47076
E												
F	114151	105594	102410	58992	59626	60085	47922	49519	49174	61310	60961	59105
G												
H												

E

	1	2	3	4	5	6	7	8	9	10	11	12
A	3		4		7		8					
B												
C												
D	16			20								
E												
F												
G												
H												

F

	1	2	3	4	5	6	7	8	9	10	11	12
A	44643	47270	45379	48102	45907	47627	48738	46757				
B												
C												
D	46110	45458	45348	43280	46035	45954						
E												
F												
G												
H												

Figure S12. Raw data of bioluminescence readings of fecal samples during CDI from Day 1-4 post-infection replicate 2. (A) 96-well plate layout with distribution of samples for Day 1, 2 and 3. (B) Bioluminescence readings recorded at 460 nm from Day 1 post-infection fecal samples. (C) Bioluminescence readings recorded at 460 nm from Day 2 post-infection fecal samples. (D) Bioluminescence readings recorded at 460 nm from Day 3 post-infection fecal

samples. (E) 96-well plate layout with distribution of samples for Day 4. (F) Bioluminescence readings recorded at 460 nm from Day 4 post-infection fecal samples.

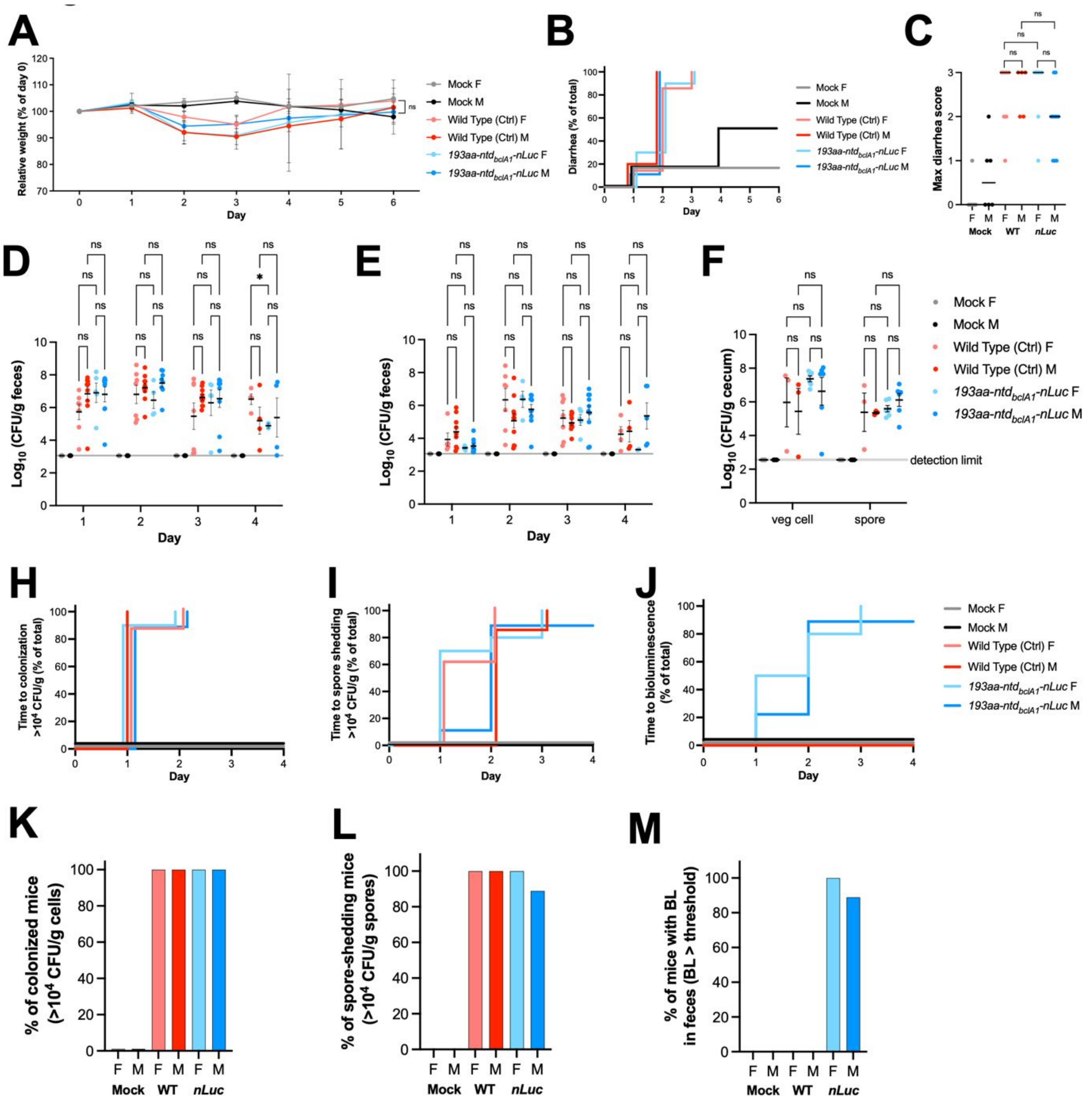

Figure S13. Sex-stratified analysis of NanoLu*c*-tagged *C. difficile* strain dynamics in a murine CDI model. (A) Relative weight change (% of day 0) for Mock, Wild type, and *193aa-ntd$_{bclA1}$-nLuc* infected mice throughout 6 days post-infection, with data shown separately for females (F) and males (M). (B) Kaplan-Meier curves for time to diarrhea onset across infection groups and sexes. (C) Maximum diarrhea scores recorded during infection, separated by infection group and sex. (D-F) Fecal colonization (D), spore shedding (E), and cecum colonization (F) over time, with data stratified by sex. (H-J) Kaplan-Meier plots for time to key

infection milestones: colonization (H), spore shedding (I), and bioluminescence detection above threshold (J), by sex and infection group. (K-M) Percentages of colonized mice (K), spore shedding mice (L), and mice with bioluminescence above threshold in feces (M) at any point during infection, separated by sex. Bioluminescence positive threshold determined as $\mu + 2.81 \times \sigma$. (A-M) Data represent combined results from two independent experiments with the following group sizes: Mock (n = 6 males, 6 females), wild type (n = 5 males, 7 females), *193aa-ntd$_{bclA1}$-nLuc* (n = 10 males, 10 females). Each symbol represents an individual mouse; bars and curves indicate group means ± SEM or cumulative incidence. Statistical analyses were performed using one-way ANOVA with Tukey's multiple comparisons test. Significance levels are indicated as follows: $P < 0.05$ (*), $P < 0.01$ (**), $P < 0.001$ (***), and $P < 0.0001$ (****). Groups that share a letter are not significantly different, groups with different letters differ significantly ($P < 0.05$).

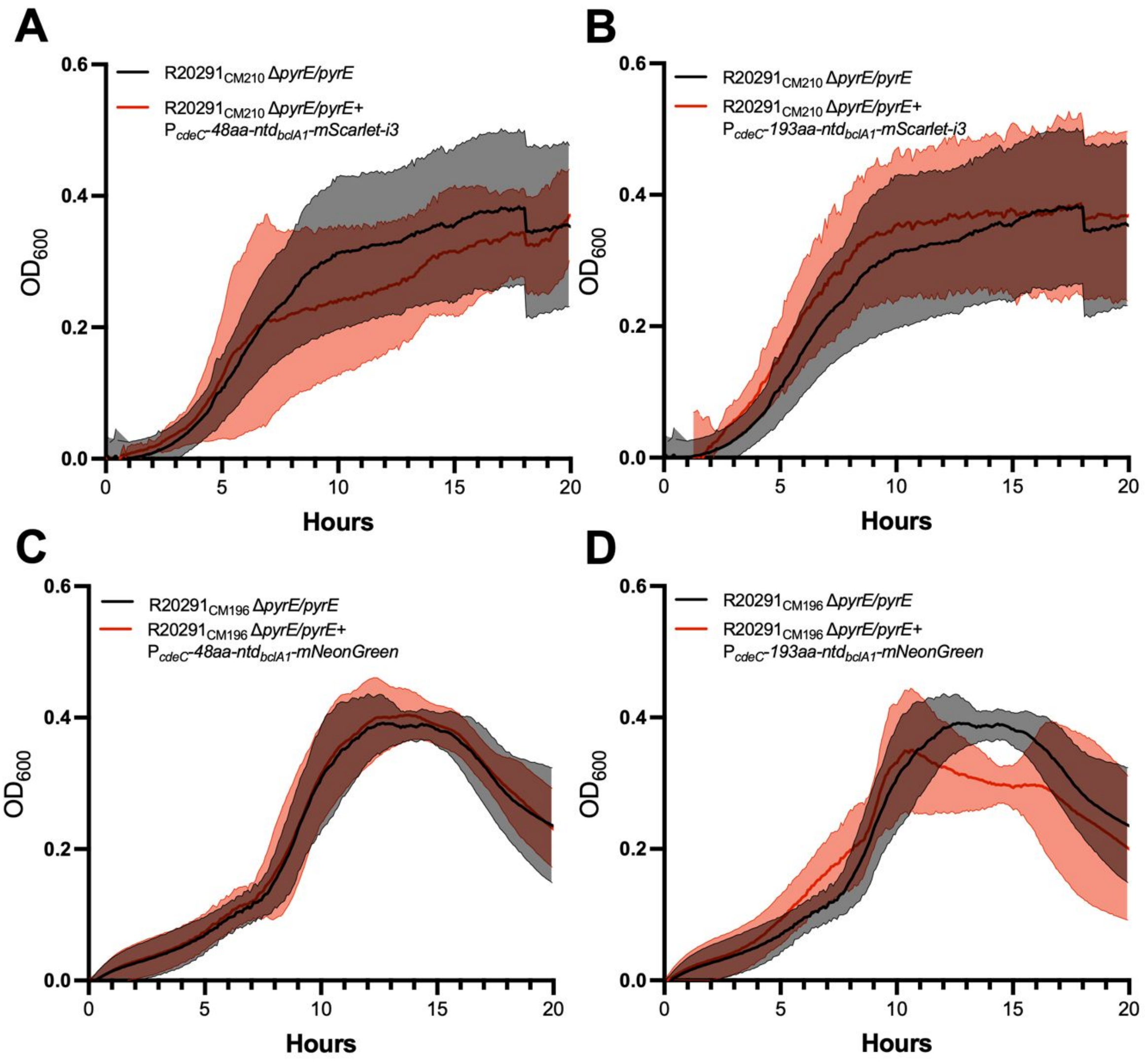

Figure S14. Growth curves of *C. difficile mScarlet-i3* and *mNeonGreen* spore tagged

mutants compared to R20291 *ΔpyrE/pyrE+* control strain. Panels (A-D) display the growth curves of mutant strains cultured anaerobically, with OD_{600} measured over time. The strains shown in panels (A) through (D) are: (A) $R20291_{CM210}$ Δ*pyrE/pyrE*+ P_{cdeC}-*48aa-ntd*$_{bclA1}$-*mScarlet-i3*; (B) $R20291_{CM210}$ Δ*pyrE/pyrE*+ P_{cdeC}-*193aa-ntd*$_{bclA1}$-*mScarlet-i3*; (C) $R20291_{CM196}$ Δ*pyrE/pyrE*+ P_{cdeC}-*48aa-ntd*$_{bclA1}$-*mNeonGreen*; and (D) $R20291_{CM196}$ Δ*pyrE/pyrE*+ P_{cdeC}-*193aa-ntd*$_{bclA1}$-*mNeonGreen*. The $R20291_{CM210}$ or $R20291_{CM196}$ Δ*pyrE/py*rE control strain growth is included as a black line in each panel for comparison. Data represent the mean of three independent biological replicates, with standard deviation indicated as shaded error bands.

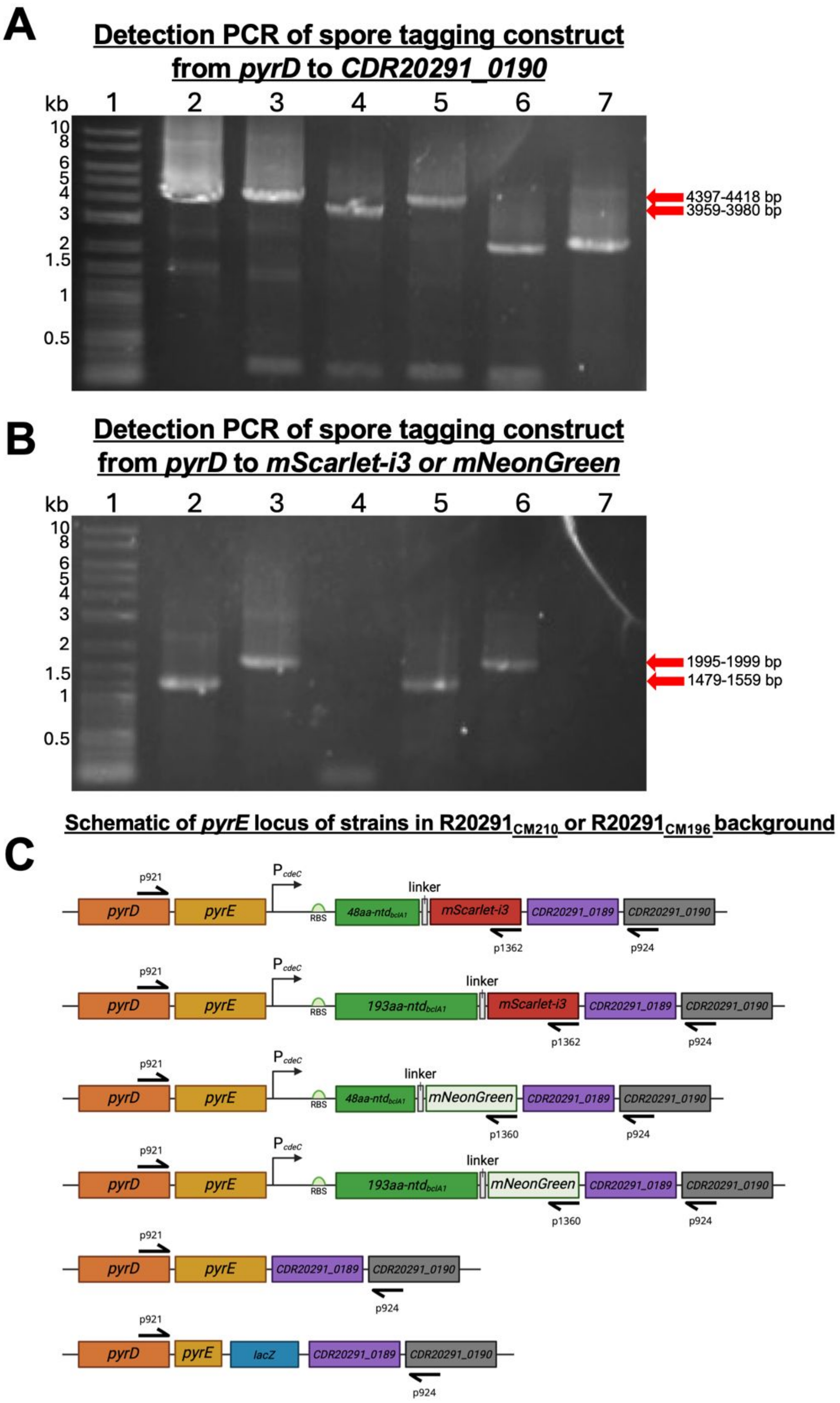

Figure S15. PCR detection of *mScarlet-i3* and *mNeonGreen* and growth curves of spore tagged mutants. (A) Agarose gel electrophoresis of PCR products amplified from genomic DNA using primers that anneal to the upstream and downstream genes flanking the *pyrE* locus. Lane 1: ZR 1kb DNA marker (Zymo) (kb); Lane 2: R20291$_{CM210}$ Δ*pyrE/pyrE*+ P$_{cdeC}$-*48aa-ntd*$_{bclA1}$-*mScarlet-i3*; Lane 3: R20291$_{CM210}$ Δ*pyrE/pyrE*+ P$_{cdeC}$-*193aa-ntd*$_{bclA1}$-*mScarlet-i3*; Lane 4: R20291$_{CM196}$ Δ*pyrE/pyrE*+ P$_{cdeC}$-*48aa-ntd*$_{bclA1}$-*mNeonGreen*; Lane 5: R20291$_{CM196}$

Δ*pyrE/pyrE*+ P_{cdeC}-*193aa-ntd*$_{bclA1}$-*mNeonGreen*; Lane 6: R20291$_{CM210}$ WT; Lane 7: R20291$_{CM210}$ Δ*pyrE*. Mutant strains, each showing a band of ~3903-4419 bp, larger than wild type control (lane 6; 2373 bp) and the Δ*pyrE* deletion control (lane 7; 2465 bp). (B) PCR confirmation of *mScarlet-i3* and *mNeonGreen* fluorescent reporter insertion, using a forward primer annealing outside *pyrE* loci and a reverse primer binding within *mScarlet-i3* or *mNeonGreen*. Lane 1: ZR 1kb DNA marker (Zymo) (kb); Lane 2: R20291$_{CM210}$ Δ*pyrE/pyrE*+ P_{cdeC}-*48aa-ntd*$_{bclA1}$-*mScarlet-i3*; Lane 3: R20291$_{CM210}$ Δ*pyrE/pyrE*+ P_{cdeC}-*193aa-ntd*$_{bclA1}$-*mScarlet-i3*; Lane 4: R20291$_{CM210}$ WT; Lane 5: R20291$_{CM196}$ Δ*pyrE/pyrE*+ P_{cdeC}-*48aa-ntd*$_{bclA1}$-*mNeonGreen*; Lane 6: R20291$_{CM196}$ Δ*pyrE/pyrE*+ P_{cdeC}-*193aa-ntd*$_{bclA1}$-*mNeonGreen*; Lane 7: R20291$_{CM196}$ WT. Mutant strains with *mScarlet-i3* (lanes 2-3) yield a product of 1559 and 1999 bp and mutant strains with *mNeonGreen* (lanes 5-6) yield a product of 1479 and 1995 bp, confirming fluorescent gene insertion. No amplification is observed in the wild-type controls (lanes 4 and 7). (C) Schematic of *pyrE* locus of strains in R20291$_{CM210}$ or R20291$_{CM196}$ background with locations of primers used for PCR detection.

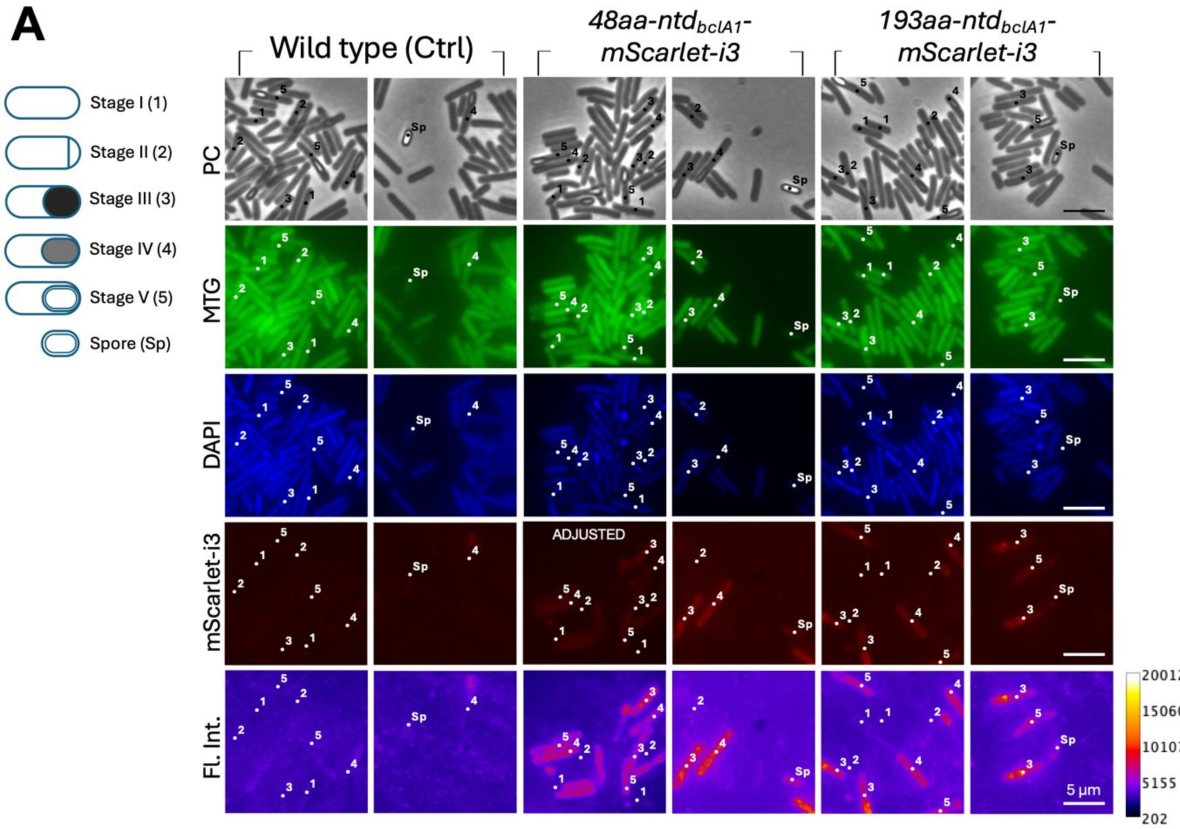

Figure S16. *mScarlet-i3* expression during *C. difficile* sporulation. (A) Schematic representation of constructs expressing *mScarlet-i3* fused to either a 48aa or 193aa *ntd*$_{bclA1}$, driven by the native *cdeC* promoter and inserted at the *pyrE* locus. Each construct includes a ribosome binding site (RBS), linker region, and terminator sequences. (B) Left, schematic of sporulation stages with corresponding stage number used for identification. Right, multichannel fluorescence microscopy of wild-type (WT) control, *48aa-ntd*$_{bclA1}$-*mScarlet-i3*, and *193aa-*

ntd_{bclA1}-*mScarlet-i3*. For each strain, panels show phase contrast (PC), membrane stain (MTG, green), DNA stain (DAPI, blue), and *mScarlet-i3* (red) fluorescence images, alongside fluorescence intensity heatmaps (Fl. Int., pseudo-colored). Representative images highlighting key sporulation stages (I-V) and mature spores (Sp) as indicated (left). Numbered cells correspond to distinct developmental stages, as diagrammed.

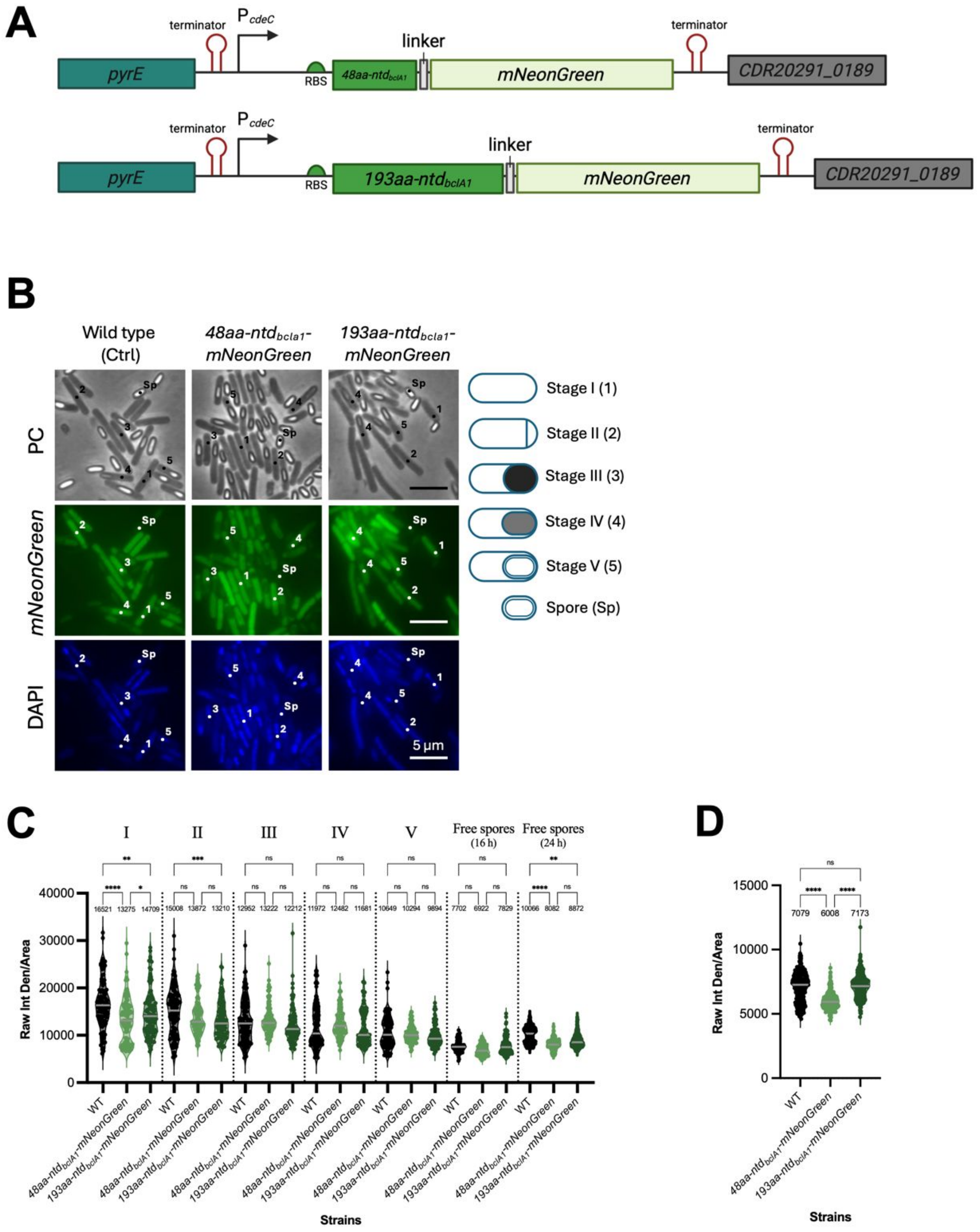

Figure S17. Analysis of *mNeonGreen* expression during *C. difficile* sporulation cycle. (A)

Schematic representation of constructs expressing *mNeonGreen* fused to either a 48-aa or 193-aa ntd_{bclA1}, driven by the native *cdeC* promoter and inserted at the *pyrE* locus. Each construct includes a ribosome binding site (RBS), linker region, and terminator sequences. (B) Multichannel fluorescence microscopy of wild type (WT) control, *48aa-*ntd_{bclA1}*-mNeonGreen*, and *193aa-*ntd_{bclA1}*-mNeonGreen* strains. For each strain, panels show phase contrast (PC), *mNeonGreen* (green), DNA stain (DAPI, blue) fluorescence images. Representative images highlighting key sporulation stages (I-V) and mature spores (Sp) as indicated (left). Numbered cells correspond to distinct developmental stages, as diagrammed. (C) Quantification of *mNeonGreen* fluorescence intensity (raw integrated density per area) across sporulation stages I-V and free spores at 16 h and 24 h, for each strain. (D) Quantification of *mNeonGreen* fluorescence intensity in purified spores at 24 h. Data in (C-D) was obtained from two independent biological replicates, with >100 cells analyzed per condition. Each data point represents an individual cell; gray lines indicate medians. Statistical significance was determined by ordinary one-way ANOVA followed by Šídák's multiple comparisons test. Significance levels are indicated as follows: $P < 0.05$ (*), $P < 0.01$ (**), $P < 0.001$ (***), and $P < 0.0001$ (****). Scale bar, 5 μM.

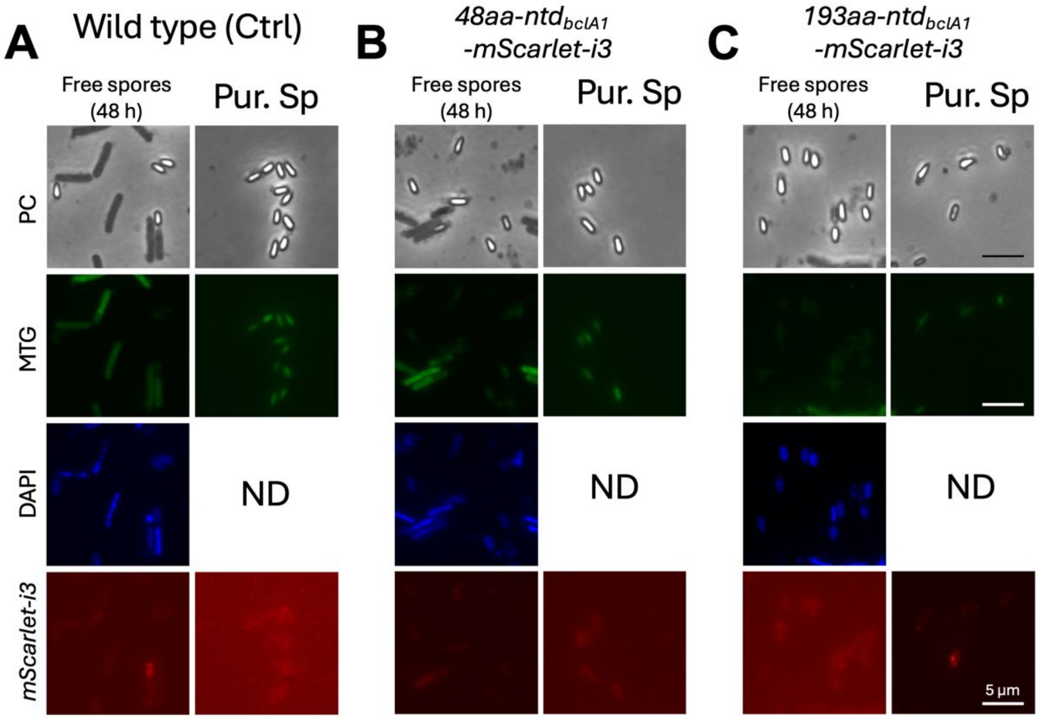

Figure S18. Representative images of *mScarlet-i3* expression in free spores (48 h) and purified *C. difficile* spores. (A-C) Representative multichannel fluorescence micrographs of spores at 48 h and purified spores. For each strain, images show phase contrast (PC), membrane stain (MTG, green), DNA stain (DAPI, blue), and *mScarlet-i3* fluorescence (red). Purified spores

were not stained for DNA (ND; not determined). (A) Wild-type (WT) spores. (B) *48aa-ntd*$_{bclA1}$-*mScarlet-i3* spores. (C) *193aa-ntd*$_{bclA1}$-*mScarlet-i3* spores. Scale bar, 5 µm.

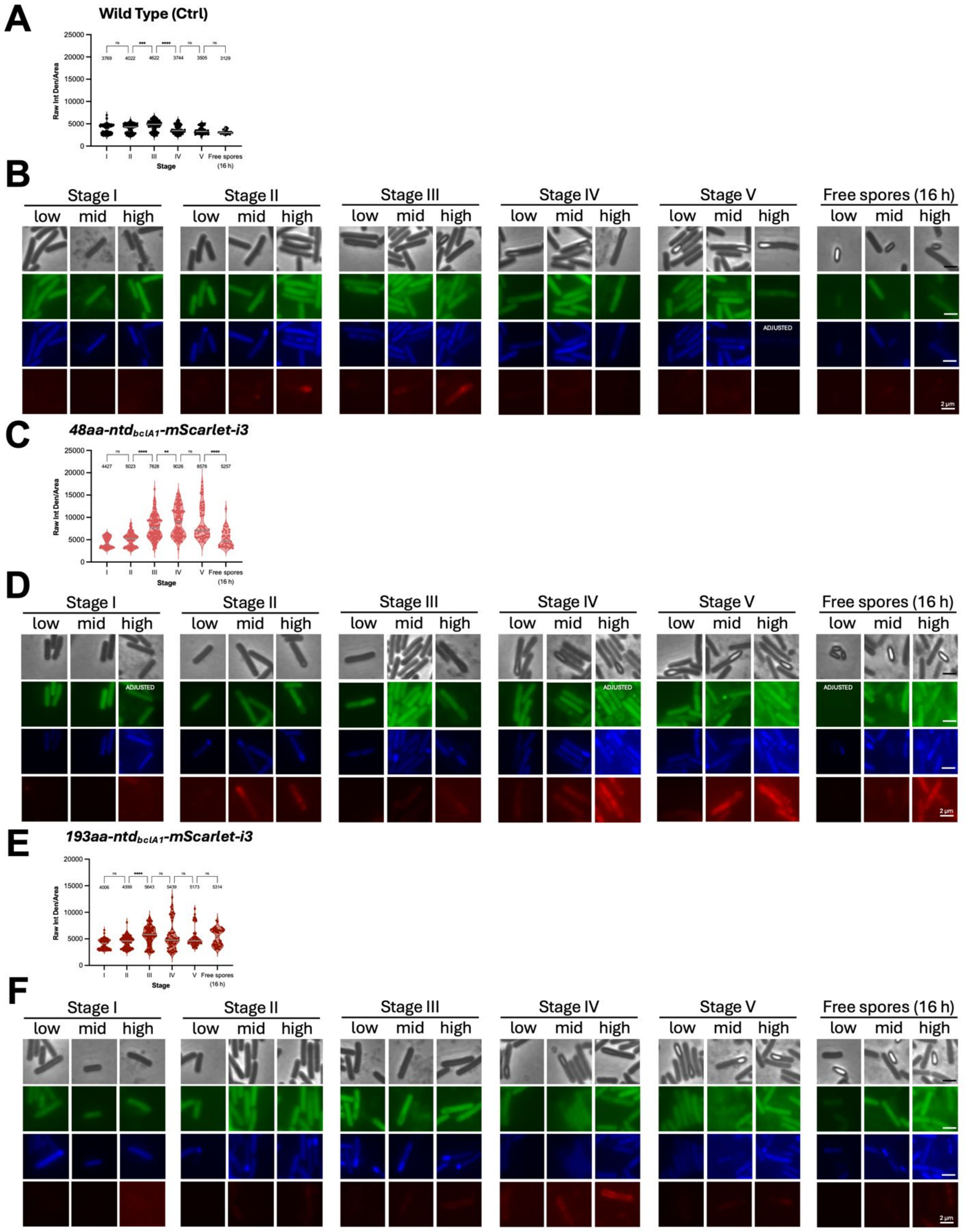

Figure S19. Heterogeneity of *mScarlet-i3* expression during *C. difficile* sporulation cycle.
(A) Quantification of fluorescence intensity as detected by mCherry/Texas Red filter set (raw integrated density per area) at different sporulation stages and in free spores at 16 h for wild-type

(WT) cells. (B) Representative multichannel fluorescence micrographs of WT cells grouped by low, mid, and high signal, across stages I-V and spores (16 h). For each group, images show phase contrast (top), membrane stain (MTG, green), DNA stain (DAPI, blue), and *mScarlet-i3* fluorescence (red). (C) Quantification of fluorescence intensity as detected by mCherry/Texas Red filter set at distinct sporulation stages and in spores (16 h) for *48aa-ntd*$_{bclA1}$*-mScarlet-i3*. (D) Representative images of *48aa-ntd*$_{bclA1}$*-mScarlet-i3* cells grouped by low, mid, and high *mScarlet-i3* signal at each stage and time point, shown as in (B). (E) Quantification of *mScarlet-i3* fluorescence intensity as detected by mCherry/Texas Red filter set at each sporulation stage and in spores (16 h) for *193aa-ntd*$_{bclA1}$*-mScarlet-i3* cells. (F) Representative images of *193aa-ntd*$_{bclA1}$*-mScarlet-i3* cells grouped by low, mid, and high *mScarlet-i3* signal across stages and time point, as above. Data in (A), (C), and (E) are shown as violin plots with individual cell values; lines indicate the median. >60 cells were analyzed per condition except for spores at 16 h (>20 cells). Representative images in (B), (D), and (F) were chosen from the lower, mid and upper ranges of per-cell fluorescence measured with mCherry/Texas Red filter set. Representative cells were picked from lowest fluorescence intensity, average fluorescence intensity and highest fluorescence intensity. Statistical comparisons were performed using ordinary one-way ANOVA assuming Gaussian distribution with Šídák's multiple comparisons test. Significance levels are indicated as follows: $P < 0.05$ (*), $P < 0.01$ (**), $P < 0.001$ (***), and $P < 0.0001$ (****). Scale bar, 2 µm.

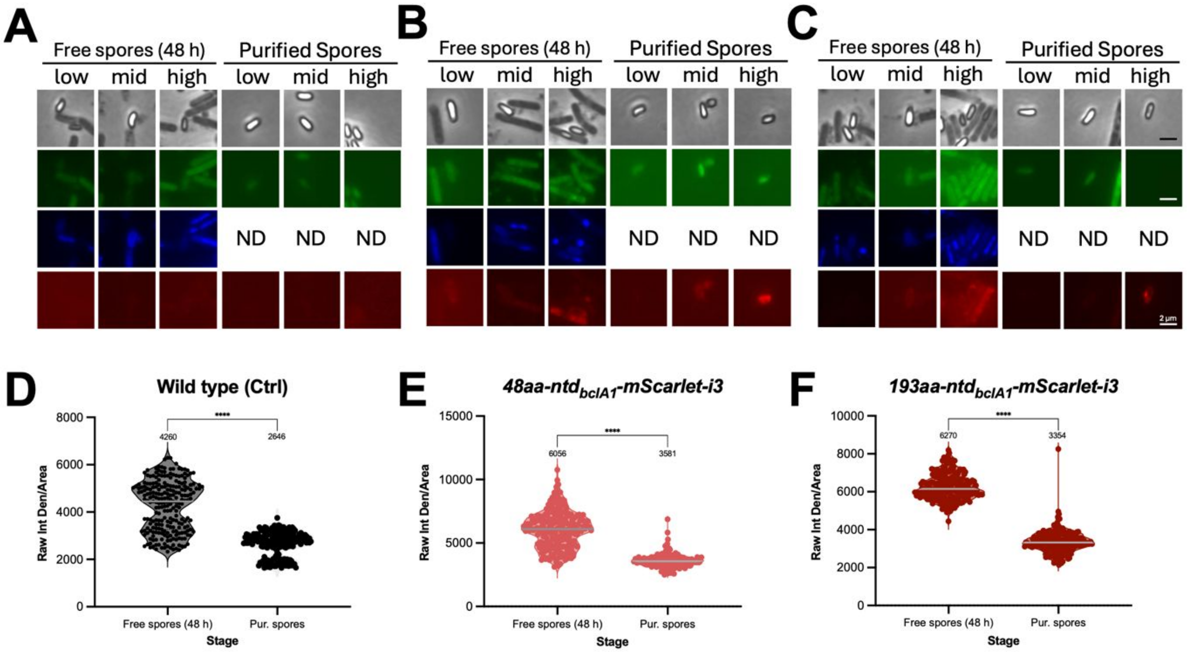

Figure S20. Heterogeneity of *mScarlet-i3* expression during the *C. difficile* sporulation cycle in free spores (48 h) and purified spores. (A) Representative multichannel fluorescence

micrographs of wild type grouped by low, mid, and high signal, across in free spores (48 h) and purified spores. For each group, images show phase contrast (top), membrane stain (MTG, green), DNA stain (DAPI, blue), and *mScarlet-i3* fluorescence (red). Purified spores were not stained for DNA (ND; not determined). (B) Representative images of *48aa-ntd*$_{bclA1}$*-mScarlet-i3* grouped by low, mid, and high *mScarlet-i3* signal for free spores (48 h) and purified spores, shown as in (A). (C) Representative images of *193aa-ntd*$_{bclA1}$*-mScarlet-i3* grouped by low, mid, and high *mScarlet-i3* signal across stages and time point, as shown in (A). (D) Quantification of red fluorescence intensity as detected by mCherry/Texas Red filter set (raw integrated density per area) at different in free spores (16 h) and purified spores for wild type (WT). (E) Quantification of *mScarlet-i3* fluorescence intensity as detected by mCherry/Texas Red filter set in free spores (16 h) and purified spores for *48aa-ntd*$_{bclA1}$*-mScarlet-i3*. (F) Quantification of *mScarlet-i3* fluorescence intensity as detected by mCherry/Texas Red filter set in free spores (16 h) and purified spores for *193aa-ntd*$_{bclA1}$*-mScarlet-i3*. Data in (D-E) is shown as violin plots with individual spore values; lines indicate the median. >60 spores were analyzed per condition. Representative images in (A-C), were chosen from the lower, mid and upper ranges of per-cell fluorescence measured with mCherry/Texas Red filter set. Representative spores were picked from lowest fluorescence intensity, average fluorescence intensity and highest fluorescence intensity. Statistical comparisons were performed using ordinary one-way ANOVA assuming Gaussian distribution with Šídák's multiple comparisons test. Significance levels are indicated as follows: $P < 0.05$ (*), $P < 0.01$ (**), $P < 0.001$ (***), and $P < 0.0001$ (****). Scale bar, 2 µm.

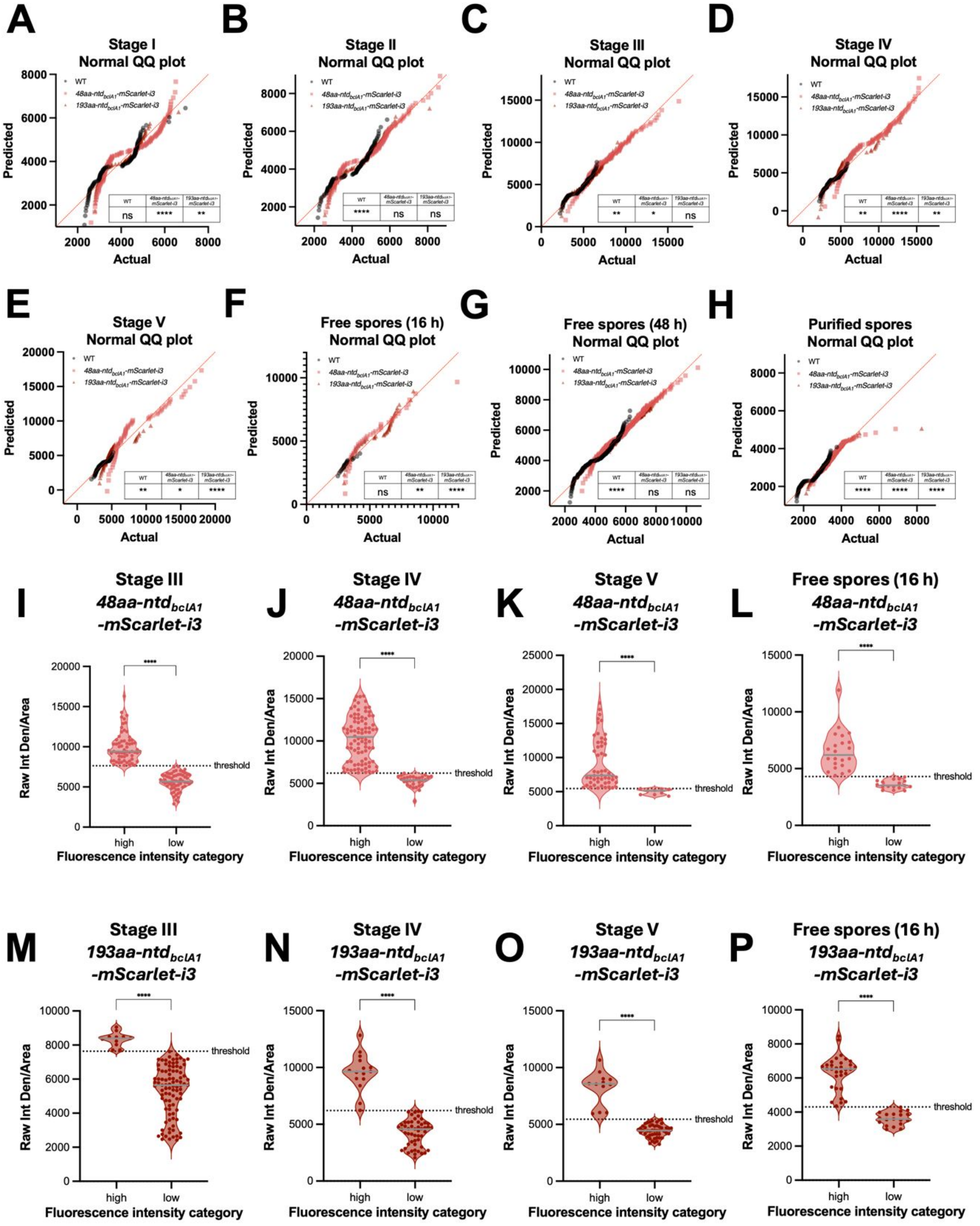

Figure S21. Normality and per-cell fluorescence distributions across sporulation stages in *mScarlet-i3* tagged strains. (A-H) Normal Q-Q plot of raw integrated density per area values used to assess Gaussianity for *mScarlet-i3* expression in each sporulation stage, free spores at 16 h and 48 h, and purified spores. Normality was evaluated with the D'Agostino-Pearson omnibus test (α = 0.05). (I-P) Violin plots display per-cell fluorescence distributions for the indicated

constructs at stages III-V and in free spores (16 h), split into high versus low intensity categories relative to each dataset threshold. Threshold was calculated using the mean (μ) and standard deviation (σ) of fluorescence intensities for control spores (Wild type (WT)), and defined the threshold as $\mu + 2.576 \times \sigma$. Individual cells are overlaid, and horizontal lines denote median. Within each stage, high versus low categories were compared using two-tailed unpaired Welch's t-test. Statistical significance annotated as * $P < 0.05$, ** $P < 0.01$, *** $P < 0.001$, and **** $P < 0.0001$.

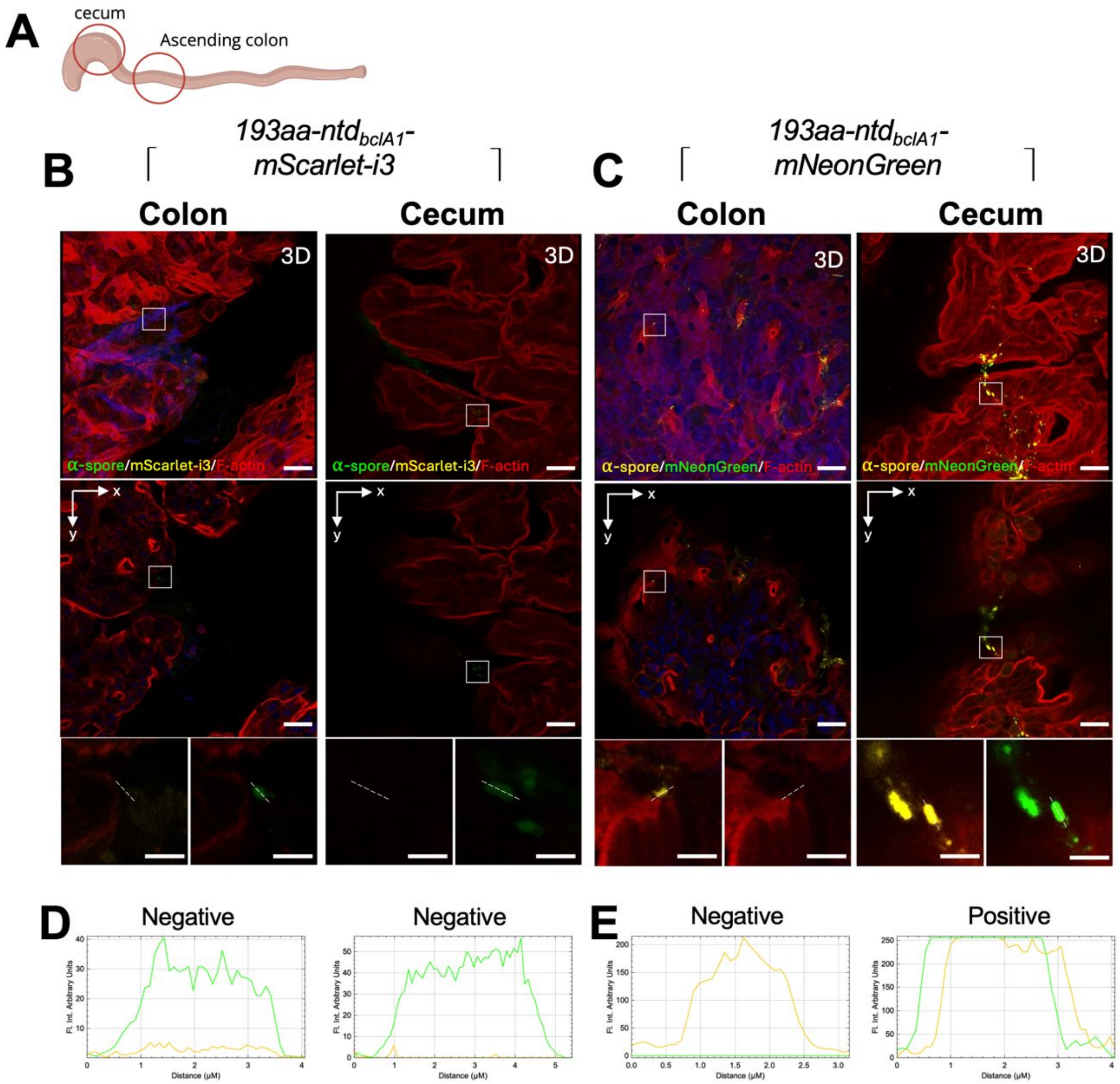

Figure S22. Confocal imaging of *mNeonGreen* and *mScarlet-i3*-tagged *C. difficile* spores formed during infection in a murine model of CDI. (A) Schematic of GI tract with highlighted extracted sections, cecum and colon. (B-C) Confocal z-stacks of colon and cecum tissue from mice

infected with *193aa-ntd*$_{bclA1}$*-mScarlet-i3* or *193aa-ntd*$_{bclA1}$*-mNeonGreen* strain with 3D renderings and magnified insets; channels display F-actin (red), nuclei (DAPI, blue), anti-spore immunolabeling (α-spore), and reporter fluorescence where indicated (*mScarlet-i3* in red; *mNeonGreen* in green). (D) Plot profiles of fluorescence intensity of *C. difficile* spores detected in green or red-orange channels from colon and cecum tissues infected with *193aa-ntd*$_{bclA1}$*-mScarlet-i3* strain. (E) Plot profiles of fluorescence intensity of *C. difficile* spores detected in green or red-orange channels from colon and cecum tissues infected with *193aa-ntd*$_{bclA1}$*-mNeonGreen* strain.

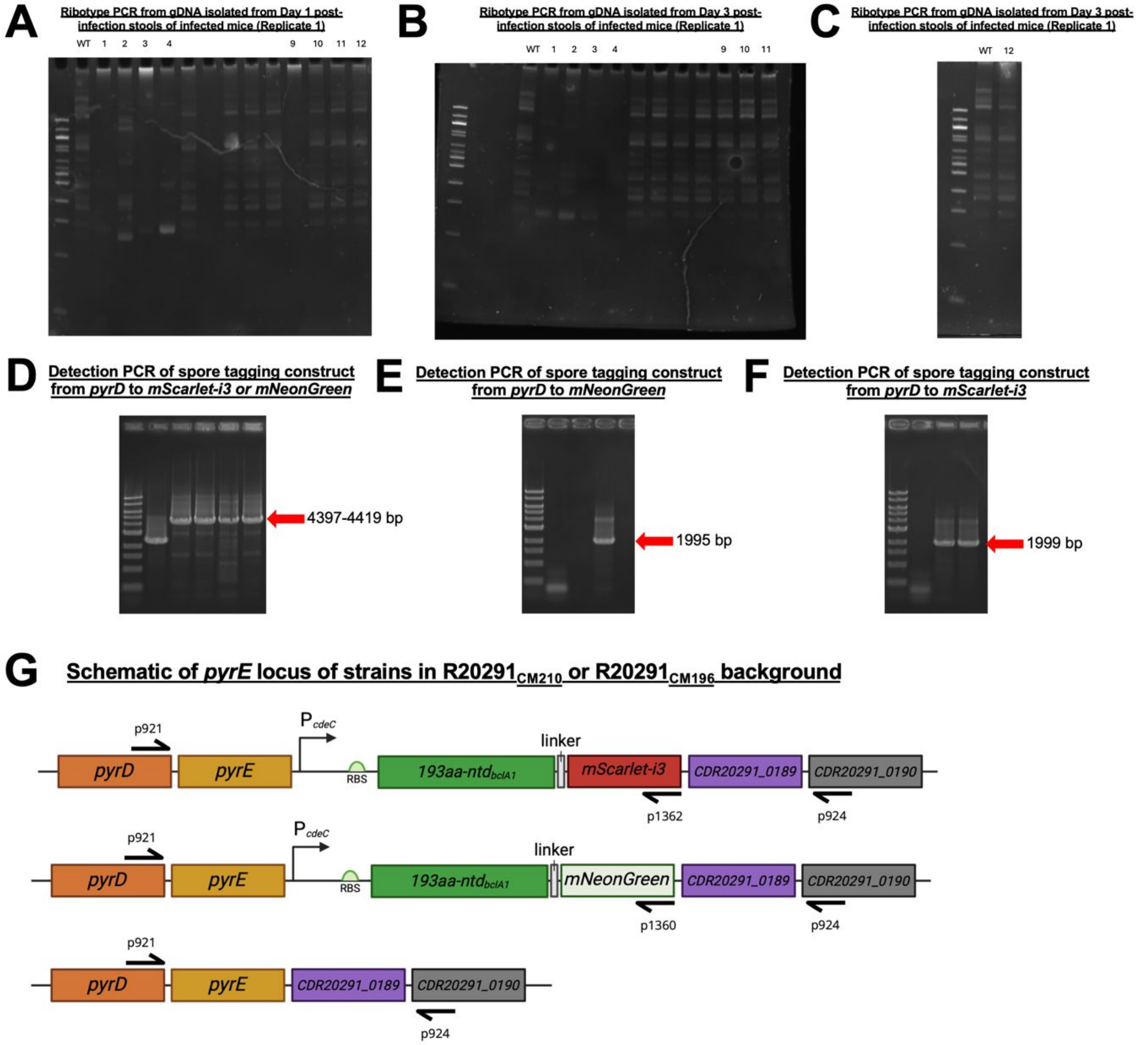

Figure S23. Ribotype PCR and PCR detection of infectious strain identity in mouse isolates. (A-C) Ribotype PCR profiles of isolates from fecal samples Day 1 and 3 post-infection.

Bands correspond to amplification of 16S and 23S rRNA regions. Lanes labeled as 1-4 are for mock controls infected with PBS-saline, lanes labeled as 9-10 are for *193aa-ntd*$_{bclA1}$*-mNeonGreen* infected mice, and 11-12 for *193aa-ntd*$_{bclA1}$*-mScarlet-i3* compared with wild type (WT) control, confirming mice were infected with the correct ribotype matching the input R20291$_{CM210}$ or R20291$_{CM196}$ infectious strain. (D) Agarose gel electrophoresis of PCR products amplified from genomic DNA from isolates using primers that anneal to the upstream and downstream genes flanking the *pyrE* locus. Lane 1: ZR 1kb DNA marker (Zymo) (kb); Lane 2: R20291$_{CM210}$ Δ*pyrE/pyrE+;* Lane 3: isolate from infected mouse 9; Lane 4: isolate from infected mouse 10 ; Lane 4: isolate from infected mouse 11; Lane 5: isolate from infected mouse 12. Isolates from infected mice with *193aa-ntd*$_{bclA1}$*-mNeonGreen* or 1*93aa-ntd*$_{bclA1}$*-mScarlet-i3* strains, each showing a band of ~4397-4419 bp, larger than wild type control (lane 6; 2373 bp).(E) PCR confirmation of *mNeonGreen* fluorescent reporter presence in genomic DNA from isolates, using a forward primer annealing outside *pyrE* loci and a reverse primer binding within *mNeonGreen*. Lane 1: ZR 1kb DNA marker (Zymo) (kb); Lane 2: R20291$_{CM196}$ Δ*pyrE/pyrE+*; Lane 3: isolate from infected mouse 9; Lane 4: isolate from infected mouse 10. PCR product of 1995bp, confirms fluorescent gene presence in isolate from mouse 10 (lane 4). No amplification is observed in the wild type control (lane 2). (F) PCR confirmation of *mScarlet-i3* fluorescent reporter presence in genomic DNA from isolates, using a forward primer annealing outside *pyrE* loci and a reverse primer binding within *mScarlet-i3*. Lane 1: ZR 1kb DNA marker (Zymo) (kb); Lane 2: R20291$_{CM210}$ Δ*pyrE/pyrE+*; Lane 3: isolate from infected mouse 11; Lane 4: isolate from infected mouse 12. PCR product of 1999bp, confirms fluorescent gene presence in isolates from mice 11 and 11 (lanes 3 and 4). No amplification is observed in the wild type control (lane 2). (G) Schematic of *pyrE* locus of strains in R20291$_{CM210}$ or R20291$_{CM196}$ background with locations of primers used for PCR detection.
