# Supplementary material for: Development and Evaluation of Exosporium-Anchored Bioluminescent and Fluorescent Reporters for Tracking Clostridioides difficile Spores Formed In Vivo
Source: ACS Synth Biol. 2026 May 15;15(6):2338–55. doi: 10.1021/acssynbio.5c00961 (PMC13288923; doi:10.1021/acssynbio.5c00961)
Supplement: Supplementary file 2 [file sb5c00961_si_002.pdf]

Development and evaluation of exosporium-anchored bioluminescent and fluorescent reporters for tracking *Clostridioides difficile* spores produced *in vivo*.

Osiris K. Lopez-Garcia[1,2], Trey Hejtmancik[1], Marjorie Pizarro-Guajardo[1], Lindsey Brehm[1], Christian Brito-Silva[3], and Daniel G. Paredes-Sabja[1,2,4*]

[1]Department of Biology, Texas A&M University, College Station, Texas, U.S.A.

[2]Interdisciplinary Graduate Program in Genetics & Genomics, Texas A&M University, College Station, Texas, U.S.A.

[3]ANID – Millennium Science Initiative Program – Millennium Nucleus in the Biology of the Intestinal Microbiota, Santiago, Chile.

[4]Department of Biology, Texas A&M University, College Station, Texas, U.S.A, Email: dparedes-sabja@tamu.edu

Table of Contents Supporting Information

Extended Materials and Methods, and Results

Tables S1. Bacterial strains used

Table S2. Plasmids used.

Table S3. Primers used.

Figure S1. Deletion of *pyrE* fragment in $R20291_{CM196}$ *C. difficile* strain.

Figure S2. Restoration of *pyrE* fragment in $R20291_{CM196}$ *ΔpyrE C. difficile* strain.

Figure S3. Promoter regulation and phenotypic characterization of bioluminescent spore-tagged strains in *C. difficile*.

Figure S4. Confirmation of insertion of *nLuc* in spore tagged mutants by PCR.

Figure S5. Growth curves of *C. difficile* NanoLuc spore tagged mutants compared to the R20291 Δ*pyrE*/*pyrE*+ control strain.

Figure S6. Confirmation of insertion of *nLuc* and growth curves of spore tagged mutants.

Figure S7. Transmission electron microscopy of NanoLuc-tagged and wild-type *C. difficile* spores.

Figure S8. Raw images of western blots and SDS-PAGE gel of purified *C. difficile* spores.

Figure S9. Raw data of bioluminescence readings for dynamic range analysis of NanoLuc-tagged *C. difficile* spores in fecal samples.

Figure S10. Ribotype PCR and PCR detection of infectious strain identity in mouse isolates.

Figure S11. Raw data of bioluminescence readings of fecal samples during CDI from Day 1-4 post-infection replicate 1.

Figure S12. Raw data of bioluminescence readings of fecal samples during CDI from Day 1-4 post-infection replicate 2.

Figure S13. Sex-stratified analysis of NanoLuc-tagged *C. difficile* strain dynamics in a murine CDI model.

Figure S14. Growth curves of *C. difficile mScarlet-i3* and *mNeonGreen* spore tagged mutants compared to R20291 *ΔpyrE/pyrE*+ control strain.

Figure S15. PCR detection of *mScarlet-i3* and *mNeonGreen* and growth curves of spore tagged mutants.

Figure S16. *mScarlet-i3* expression during *C. difficile* sporulation.

Figure S17. Analysis of *mNeonGreen* expression during *C. difficile* sporulation cycle.

Figure S18. Representative images of *mScarlet-i3* expression in free spores (48 h) and purified *C. difficile* spores.

Figure S19. Heterogeneity of *mScarlet-i3* expression during *C. difficile* sporulation cycle.

Figure S20. Heterogeneity of *mScarlet-i3* expression during the *C. difficile* sporulation cycle in free spores (48 h) and purified spores.

Figure S21. Normality and per-cell fluorescence distributions across sporulation stages in *mScarlet-i3* tagged strains.

Figure S22. Confocal imaging of *mNeonGreen* and *mScarlet-i3*-tagged *C. difficile* spores formed during infection in a murine model of CDI.

Figure S23. Ribotype PCR and PCR detection of infectious strain identity in mouse isolates.
