# Supplementary material for: Development and Evaluation of Exosporium-Anchored Bioluminescent and Fluorescent Reporters for Tracking Clostridioides difficile Spores Formed In Vivo
Source: ACS Synth Biol. 2026 May 15;15(6):2338–55. doi: 10.1021/acssynbio.5c00961 (PMC13288923; doi:10.1021/acssynbio.5c00961)
Supplement: Supplementary file 3 [file sb5c00961_si_003.pdf]

Development and evaluation of exosporium-anchored bioluminescent and fluorescent reporters for tracking *Clostridioides difficile* spores produced *in vivo*.

Osiris K. Lopez-Garcia[1,2], Trey Hejtmancik[1], Marjorie Pizarro-Guajardo[1], Lindsey Brehm[1], Christian Brito-Silva[3], and Daniel G. Paredes-Sabja[1,2,4*]

[1]Department of Biology, Texas A&M University, College Station, Texas, U.S.A.

[2]Interdisciplinary Graduate Program in Genetics & Genomics, Texas A&M University, College Station, Texas, U.S.A.

[3]ANID – Millennium Science Initiative Program – Millennium Nucleus in the Biology of the Intestinal Microbiota, Santiago, Chile.

[4]Department of Biology, Texas A&M University, College Station, Texas, U.S.A, Email: dparedes-sabja@tamu.edu

S1 Extended Material and Methods

Bacterial strains and growth conditions

Escherichia coli strains (Table S1) were routinely grown aerobically at 37 °C under aerobic conditions with shaking at 1 × g in Luria-Bertani medium, supplemented with 10 µg/mL tetracycline, 50 µg/mL kanamycin, 50 µg/mL chloramphenicol or 100 µg/mL ampicillin, where appropriate. *C. difficile* strains (Table S1) were routinely grown at 37 °C under anaerobic conditions in a Coy Laboratory anaerobic chamber (4% H_2, 5% CO_2, 85% N_2) in BHIS medium: 3.7% (w/v) brain heart infusion broth supplemented with 0.5% (w/v) yeast extract (Difco) and 0.1% (w/v) L-cysteine or on BHIS agar plates.

For mutant construction, a defined *C. difficile* minimal medium (CDMM) media was prepared when performing genetic selections as described [44-46]. For CDMM reagents preparation, 5x amino acids (50 mg/mL casamino acids, 2.5 mg/mL L-tryptophan, and 2.5 mg/mL L-cysteine), 10x salts (50 mg/mL Na_2HPO_4, 50 mg/mL $NaHCO_3$, 9 mg/mL KH_2PO_4, and 9 mg/mL NaCl), 20x glucose (200 mg/mL d-glucose), 50x trace salts (2.0 mg/mL $(NH_4)_2SO_4$, 1.3 mg/mL $CaCl_2{\cdot}2H_2O$, 1.0 mg/mL $MgCl_2{\cdot}6H_2O$, 0.5 mg/mL $MnCl_2{\cdot}4H_2O$, and 0.05 mg/mL $CoCl_2{\cdot}6H_2O$), 100x iron (0.4 mg/mL $FeSO_4{\cdot}7H_2O$), and 100x vitamins (0.1 mg/mL D-biotin, 0.1 mg/mL calcium-d-pantothenate, and 0.1 mg/mL pyridoxine) stock solutions were made by dissolving reagents in Milli-Q water and filter sterilizing (0.2-µm pore size) prior to use. Solutions were mixed to obtain CDMM media with final concentration of 10 mg/mL casamino acids, 0.5 mg/mL L-tryptophan, 0.5 mg/mL L-cysteine, 5 mg/mL Na_2HPO_4, 5 mg/mL $NaHCO_3$, 0.9 mg/mL KH_2PO_4, 0.9 mg/mL NaCl, 10 mg/mL D-glucose, 0.04 mg/mL $(NH_4)_2SO_4$, 0.026 mg/mL $CaCl_2{\cdot}2H_2O$, 0.02 mg/mL $MgCl_2{\cdot}6H_2O$, 0.01 mg/mL $MnCl_2{\cdot}4H_2O$, 0.001 mg/mL $CoCl_2{\cdot}6H_2O$, 0.004 mg/mL $FeSO_4{\cdot}7H_2O$, 0.001 mg/mL d-biotin, 0.001 mg/mL calcium-D-pantothenate, and 0.001 mg/mL pyridoxine. For solid medium, agar (BD, USA) was added to CDMM for a final concentration of 1.0% weight/vol. Finally, media were supplemented with uracil (Sigma-Aldrich, USA) at 5 mg/mL to a final concentration of 5 µg/mL and 5-fluoroorotic acid (5-FOA; USBiological, USA) at 100 mg/mL for a final concentration of 2 mg/mL as needed.

Construction of CM196 Δ*pyrE C. difficile* strain

The allele-exchange cassette was assembled into pMTL-YN4, an allele-exchange vector optimized for *pyrE*-based selection in *C. difficile*, using Gibson assembly workflow in *E. coli* (Table S2) [45].

pMTL-YN4 derives from the pMTL80000 modular series and functions as the KO delivery vector in the YN4 system for *C. difficile* allelic exchange [45]. Construction of pMTL-YN4-*ΔpyrE* was done using primers p974 (FP DpyrE-LHA GC) and p975 (RP DpyrE-RHA GC) to amplify *ΔpyrE* loci from R20291 Δ*pyrE* which was then inserted in between AscI and SbfI restriction sites of the pMTL-YN4 vector (Table S2, Table S3). Resulting plasmid was sequenced confirmed using Sanger sequencing by Eton Bioscience (San Diego, CA).

To generate mutant, deletion of *pyrE* in *C. difficile* $R20291_{CM196}$ was made by allelic exchange [45]. Conjugation of plasmid carrying pMTL-YN4-Δ*pyrE* was performed by transforming plasmid into *E. coli* CA434 and mating with *C. difficile* $R20291_{CM196}$ [45]. Transconjugants were selected by subculturing on BHIS agar containing 15 µg/mL thiamphenicol, 16 µg/mL cefoxitin and 250 µg/mL cycloserine and re-streaked five times. Single-crossover mutants identified were streaked onto CDMM with 1.5% (w/v) agar supplemented with 2 mg/mL 5-FOA (USBiological, USA) and 5 µg/mL uracil (Sigma-Aldrich, USA) to select for deletion of *pyrE* fragment [44-46]. Replicate plating on BHIS and BHIS plates containing 15 µg/mL thiamphenicol was done to select transconjugants without thiamphenicol resistance to ensure resistance cassette from plasmid is not being expressed. Colonies sensitive to thiamphenicol were screened by PCR to confirm deletion of *pyrE* by using primers p921 (FP pyrD) and p924 (RP 0190), and insertion of *lacZ-alpha* fragment using primers p921 (FP pyrD) and p946 (RP pyrE lacZ det) (Fig S1A-C). Growth curve analyses were performed to exclude growth defects (Fig S1D). Whole-genome sequencing was conducted on mutant strain using Illumina sequencing by SeqCenter (Pittsburgh, PA) to confirm the absence of off-target mutations and validate the intended genetic modifications (Fig S1E-F).

Restoration of *pyrE* loci in *C. difficile* CM196 Δ*pyrE* was made by allelic exchange [45]. Conjugation of plasmid carrying pMTL-YN2 was performed by transforming plasmid into *E. coli* CA434 and mating with *C. difficile* CM196 Δ*pyrE* [45]. Transconjugants were selected by subculturing on BHIS agar containing 15 µg/mL thiamphenicol, 16 µg/mL cefoxitin and 250 µg/mL cycloserine and re-streaked five times. Single-crossover mutants identified were streaked onto CDMM with 1.5% (w/v) agar to select for restoration of *pyrE* fragment [44-46]. Replicate plating on BHIS and BHIS plates containing 15 µg/mL thiamphenicol was done to select transconjugants without thiamphenicol resistance to ensure resistance cassette from plasmid is not being expressed. Colonies sensitive to thiamphenicol were screened by PCR to confirm restoration of *pyrE* by using primers p528 (FP-pyrE detection) and p529 (RP-pyrER detection) (Fig S2A-C). Growth curve analyses were performed to exclude growth defects (Fig S2D). Whole-genome sequencing was conducted on mutant strain using Illumina sequencing by SeqCenter (Pittsburgh, PA) to confirm the absence of off-target mutations and validate the intended genetic modifications (Fig S2E-F).

Plasmid construction

Plasmids were constructed using standard molecular cloning techniques, by restriction-ligation and/or Gibson assembly in *E. coli* using pBlue-Heron (pBH), pMTL-YN2C or pMTL-YN2C-TT backbones were noted [47]. DNA fragments encoding relevant promoter, regulatory, and reporter sequences were amplified using the primers listed in Table S3.

Spore surface reporter constructs combined *48aa*- or *193aa-*ntd_{bclA1} fused to *nLuc* or fluorescence reporters *mScarlet-i3* and *mNeonGreen* under the *bclA1* (P_{bcla1}) or *cdeC* (P_{cdeC}) promoters, with or without *tetO* operator, and where specified tagging system was downstream of *tetR* driven by

rubrerythrin (CDIF27147_03030) promoter (P_{rbr}) (Table S2). The repaired *bclA1* coding sequence for strain R20291 correcting two SNPs (T145A and T739C). SNPs were repaired by three-fragment Gibson assembly using primers p631 (FP-P.BclA1Ra.Sbf), p632 (RP-P-BclA1Ra), p633 (FP-BclA1Rb), p634 (RP-BclA1Rb), p635 (FP-BclA1Rc) and p636 (RP-BclA1Rc.AscI) encoding the corrective nucleotides, and the assembled insert was cloned between the SbfI and AscI sites of the pBlue-Heron vector (Table S2).

The initial set of pBH constructs included pBH-*tetR*-P_{rbr}-P_{bclA1}-*tetO-48aa-ntd*$_{bclA1}$-*nLuc*, pBH-P_{bclA1}-*tetO-193aa-ntd*$_{bclA1}$-*nLuc* and pBH-*tetR*-P_{rbr}-P_{cdeC}-*tetO-48aa-ntd*$_{bclA1}$-*nLuc*. A 2195 bp gBlock containing *tetR* under the rubrerythrin (CDIF27147_03030) promoter and *48aa-ntd*$_{bclA1}$-*nLuc* under the *bclA1* promoter with *tetO* operator and flanked by NotI and NcoI sites was designed and cloned between the NotI and NcoI sites of pBlue-Heron vector by restriction ligation for pBH-*tetR*-P_{rbr}-P_{bclA1}-*tetO-48aa-ntd*$_{bclA1}$-*nLuc*. For pBH-P_{bclA1}-*tetO-193aa-ntd*$_{bclA1}$-*nLuc*, a 968 bp gBlock containing *193aa-ntd*$_{bclA1}$-*nLuc* under the *bclA1* promoter with *tetO* operator and flanked by XbaI and NdeI sites was designed and cloned between the XbaI and NdeI sites of pBH-*tetR*-P_{rbr}-P_{bclA1}-*tetO-48aa-ntd*$_{bclA1}$-*nLuc* by restriction ligation. For pBH-*tetR*-P_{rbr}-P_{cdeC}-*tetO-48aa-ntd*$_{bclA1}$-*nLuc*, a 373 bp gBlock containing *48aa-ntd-bclA1-nLuc* under the *cdeC* promoter with *tetO* operator and flanked by XbaI and BamHI sites was designed and cloned between the XbaI and BamHI sites of pBH-*tetR*-P_{rbr}-P_{bclA1}-*tetO-48aa-ntd*$_{bclA1}$-*nLuc* by restriction ligation (Table S2).

To introduce control expression of *tetR*, *tet* operator (*tetO*) was inserted upstream of *tetR* in P_{rbr} resulting in plasmid pBH-*tetR*-P_{rbr}-tetO-P_{bclA1}-*tetO-48aa-ntd*$_{bclA1}$-*nLuc*. Briefly, to insert *tetO* operator into P_{rbr}, the P_{rbr} region was amplified from pBH-*tetR*-P_{rbr}-P_{bclA1}-*tetO-48aa-ntd*$_{bclA1}$-*nLuc*

using overlap extension PCR. A fragment of 745 bp was amplified using primers p976 (RP Prbr+tetO) and p978 (FP TetR), and a fragment of 655 bp was amplified using primers p977 (FP Prbr+tetO) and p979 (RP PbclA1). These two fragments were combined and subjected to a second amplification with outer primers p978 (FP TetR) and p979 (RP PbclA1) to yield a full-length P_{rbr}-*tetO* fragment. The resulting P_{rbr}-*tetO* fragment was cloned into the parental plasmid pBH-*tetR*-P_{rbr}-P_{bclA1}-*tetO-48aa*-ntd_{bclA1}-*nLuc* using XhoI and XbaI sites to replace the original P_{rbr} region, yielding pBH-*tetR*-P_{rbr}-*tetO*-P_{bclA1}-*tetO-48aa*-ntd_{bclA1}-*nLuc* (Table S2).

Spore surface reporter systems using native promoters were constructed by cloning XbaI-BamHI amplicons into pBH-*tetR*-P_{rbr}-P_{bclA1}-*tetO-48aa*-ntd_{bclA1}-*nLuc* or its P_{rbr}-*tetO* derivative. P_{bclA1} native promoter was amplified from R20291 strain using primers p883 (FP-BCLA10337-PROM) and p884 (RP-BCLA10337-PROM), and inserted between XbaI and BamHI sites of pBH-*tetR*-P_{rbr}-P_{bclA1}-*tetO-48aa*-ntd_{bclA1}-*nLuc* and pBH-*tetR*-P_{rbr}-*tetO*-P_{bclA1}-*tetO-48aa*-ntd_{bclA1}-*nLuc* to yield pBH-*tetR*-P_{rbr}-P_{bclA1}-*48aa*-ntd_{bclA1}-*nLuc* and pBH-*tetR*-P_{rbr}-*tetO*-P_{bclA1}-*48aa*-ntd_{bclA1}-*nLuc* respectively. P_{cdeC} native promoter was amplified from R20291 strain using primers p887 (FP-cdeC-Prom) and p888 (RP-cdeC-Prom), and inserted between XbaI and BamHI sites of pBH-*tetR*-P_{rbr}-P_{bclA1}-*tetO-48aa*-ntd_{bclA1}-*nLuc* and pBH-*tetR*-P_{rbr}-*tetO*-P_{bclA1}-*tetO-48aa*-ntd_{bclA1}-*nLuc* to yield pBH-*tetR*-P_{rbr}-P_{cdeC}-*48aa*-ntd_{bclA1}-*nLuc* and pBH-*tetR*-P_{rbr}-*tetO*-P_{cdeC}-*48aa*-ntd_{bclA1}-*nLuc* respectively (Table S2).

To create *cdeC* variant of pBH-*tetR*-P_{rbr}-*tetO*-P_{bclA1}-*tetO-48aa*-ntd_{bclA1}-*nLuc*, a 548 bp fragment was digested from pBH-*tetR*-P_{rbr}-P_{cdeC}-*tetO-48aa*-ntd_{bclA1}-*nLuc* using XbaI and NdeI and inserted

into pBH-*tetR*-P$_{rbr}$-*tetO*-P$_{bclA1}$-*tetO-48aa-ntd*$_{bclA1}$-*nLuc* by restriction-ligation yielding pBH-*tetR*-P$_{rbr}$-*tetO*-P$_{cdeC}$-*tetO-48aa-ntd*$_{bclA1}$-*nLuc* (Table S2).

For *C. difficile* shuttle constructs, pMTL-YN2C derivatives were built by subcloning EcoRI-NcoI fragments from the corresponding pBlueHeron sources: pMTL-YN2C-*tetR*-P$_{rbr}$-P$_{bclA1}$-*48aa-ntd*$_{bclA1}$-*nLuc*, pMTL-YN2C-*tetR*-P$_{rbr}$-P$_{bclA1}$-*tetO-48aa-ntd*$_{bclA1}$-*nLuc*, pMTL-YN2C-*tetR*-P$_{rbr}$-*tetO*-P$_{bclA1}$-*48aa-ntd*$_{bclA1}$-*nLuc*, and pMTL-YN2C-*tetR*-P$_{rbr}$-*tetO*-P$_{bclA1}$-*tetO-48aa-ntd*$_{bclA1}$-*nLuc*. Analogous P$_{cdeC}$ constructs were generated as pMTL-YN2C-*tetR*-P$_{rbr}$-P$_{cdeC}$-*48aa-ntd*$_{bclA1}$-*nLuc*, pMTL-YN2C-*tetR*-P$_{rbr}$-P$_{cdeC}$-*tetO-48aa- ntd*$_{bclA1}$-*nLuc*, pMTL-YN2C-*tetR*-P$_{rbr}$-*tetO*-P$_{cdeC}$-*48aa-ntd*$_{bclA1}$-*nLuc*, and pMTL-YN2C-*tetR*-P$_{rbr}$-*tetO*-P$_{cdeC}$-*tetO-48aa-ntd*$_{bclA1}$-*nLuc* (Table S2).

To produce *193aa-ntd*$_{bclA1}$-*nLuc* variant under the control of *cdeC* promoter, a 641 bp fragment containing *193aa-ntd*$_{bclA1}$ was amplified from pBH-P$_{bclA1}$-*tetO-193aa-ntd*$_{bclA1}$-*nLuc* using primers p1277 (FP-NTD_BclA1_193aa_BamHI-GC) and p1278 (RP-NTD_BclA1_193aa_NcoI-GC), and a 655 bp fragment containing *nLuc* was amplified from pBH-*tetR*-P$_{rbr}$-P$_{cdeC}$-*48aa-ntd*$_{bclA1}$-*nLuc* using primers p1279 (FP-Nluc_SalI-GC) and p1280 (RP-Nluc_HindIII-GC) were used for Gibson assembly between BamHI and HindIII sites of linearized pMTL-YN2C-*tetR*-P$_{rbr}$-P$_{bclA1}$-*48aa-ntd*$_{bclA1}$-*nLuc* (Table S2).

To produce variants of shuttle constructs with an added native transcriptional terminator of *pyrE*, the pMTL-YN2C-TT was used as a backbone to introduce native promoters' variants without

regulation by *tetR* by restriction-ligation cloning (Javier Ref). For pMTL-YN2C-TT-P_{bclA1}-*48aa-ntd*$_{bclA1}$*-nLuc*, 1136 bp fragment containing native *bclA1* promoter and *48aa-ntd*$_{bclA1}$*-nLuc* was introduced between XbaI and HindIII sites of pMTL-YN2C-TT. For pMTL-YN2C-TT-P_{cdeC}-*48aa- ntd*$_{bclA1}$*-nLuc*, 1136 bp fragment containing native *cdeC* promoter and *48aa-ntd*$_{bclA1}$*-nLuc* was introduced between XbaI and NcoI sites of pMTL-YN2C-TT. For pMTL-YN2C-TT-P_{cdeC}-*193aa- ntd*$_{bclA1}$*-nLuc*, 636 bp fragment containing native *cdeC* promoter was introduced between NcoI and HindIII sites of pMTL-YN2C-TT-P_{cdeC}-*48aa-ntd*$_{bclA1}$*-mNeonGreen* (Table S2).

To make fluorescent spore surface reporter systems with *mNeonGreen*, a 582 bp fragment containing *cdeC* promoter and *48aa-ntd*$_{bclA1}$ was amplified from pBH-*tetR*-P_{rbr}-P_{bclA1}-*tetO-48aa-ntd*$_{bclA1}$*-nLuc* using primers p1359 (FP PcdeC-NTD-BclA1) and p1360 (RP PcdeC-NTD-BclA1 for NeonGreen), and a 759 bp fragment containing *mNeonGreen* was amplified from pMTL-YN1C-P_{cwp2}-*mNeonGreen* using primers p1361 (FP NeonGreen for YN2C-TT-PcdeC-NTD-BclA1-NeonGreen) and p1223 (2913 3' XhoI mNeonGreen Gibson) were used for Gibson assembly between BamHI and XhoI sites of pMTL-YN2C-TT to yield pMTL-YN2C-TT-P_{cdeC}-*48aa-ntd*$_{bclA1}$*-mNeonGreen*. For *193aa-ntd*$_{bclA1}$ variant, a 1024 bp fragment containing *cdeC* promoter and *193aa-ntd*$_{bclA1}$ was amplified from pBH-P_{bclA1}-*tetO-193aa-ntd*$_{bclA1}$*-nLuc* using primers p1359 (FP PcdeC-NTD-BclA1) and p1360 (RP PcdeC-NTD-BclA1 for NeonGreen), and a 759 bp fragment containing *mNeonGreen* was amplified from pMTL-YN1C-P_{cwp2}-*mNeonGreen* using primers p1361 (FP NeonGreen for YN2C-TT-PcdeC-NTD-BclA1-NeonGreen) and p1223 (2913 3' XhoI mNeonGreen Gibson) were used for Gibson assembly between BamHI and XhoI sites of pMTL-YN2C-TT to yield pMTL-YN2C-TT-P_{cdeC}-*193aa-ntd*$_{bclA1}$*-mNeonGreen* (Table S2).

To make fluorescent spore surface reporter systems with *mScarlet-i3*, a 587 bp fragment containing *cdeC* promoter and *48aa-ntd*$_{bclA1}$ was amplified from pBH-*tetR*-P$_{rbr}$-P$_{cdeC}$-*tetO-48aa-ntd*$_{bclA1}$*-nLuc* using primers p1359 (FP PcdeC-NTD-BclA1) and p1523 (RP PcdeC-NTD-BclA1 for mScarlet i3), and a 739 bp fragment containing *mScarlet-i3* was amplified from pMTL-YN1C-P$_{cwp2}$-*mScarlet-i3* using primers p1524 (FP mScarlet-i3) and p1525 (RP 2912 3' XhoI mScarlet-i3 Gibson) were used for Gibson assembly between BamHI and XhoI sites of pMTL-YN2C-TT to yield pMTL-YN2C-TT-P$_{cdeC}$-*48aa-ntd*$_{bclA1}$*-mScarlet-i3*. For *193aa-ntd*$_{bclA1}$ variant, a 1024 bp fragment containing *cdeC* promoter and *193aa-ntd*$_{bclA1}$ was amplified from pBH-P$_{bclA1}$-*tetO-193aa-ntd*$_{bclA1}$*-nLuc* using primers p1359 (FP PcdeC-NTD-BclA1) and p1523 (RP PcdeC-NTD-BclA1 for mScarlet i3), and a 739 bp fragment containing *mScarlet-i3* was amplified from pMTL-YN1C-P$_{cwp2}$-*mScarlet-i3* using primers p1524 (FP mScarlet-i3) and p1525 (RP 2912 3' XhoI mScarlet-i3 Gibson) were used for Gibson assembly between BamHI and XhoI sites of pMTL-YN2C-TT to yield pMTL-YN2C-TT-P$_{cdeC}$-*193aa-ntd*$_{bclA1}$*-mScarlet-i3* (Table S2).

All plasmid constructs were sequenced confirmed using Sanger sequencing by Eton Bioscience (San Diego, CA) or Oxford Nanopore Technology by Plasmidsaurus Inc. (Eugene, OR).

Construction of *C. difficile* reporter strains

To construct *C. difficile* strains, shuttle plasmids carrying inducible spore surface reporter systems (pMTL-YN2C-*tetR*-P$_{rbr}$-P$_{bclA1}$-*48aa-ntd*$_{bclA1}$*-nLuc*, pMTL-YN2C-*tetR*-P$_{rbr}$-P$_{bclA1}$-*tetO-48aa-ntd*$_{bclA1}$*-nLuc*, pMTL-YN2C-*tetR*-P$_{rbr}$-*tetO*-P$_{bclA1}$-*48aa-ntd*$_{bclA1}$*-nLuc*, pMTL-YN2C-*tetR*-P$_{rbr}$-*tetO*-P$_{bclA1}$-*tetO-48aa-ntd*$_{bclA1}$*-nLuc*, pMTL-YN2C-*tetR*-P$_{rbr}$-P$_{cdeC}$-*48aa-ntd*$_{bclA1}$-

nLuc, pYN2C-*tetR*-P_{rbr}-P_{cdeC}-*tetO-48aa-ntd*$_{bclA1}$*-nLuc*, pMTL-YN2C-*tetR*-P_{rbr}-*tetO*-P_{cdeC}-*48aa-ntd*$_{bclA1}$*-nLuc*, and pMTL-YN2C-*tetR*-P_{rbr}-*tetO*-P_{cdeC}-*tetO-48aa-ntd*$_{bclA1}$*-nLuc*) (Table S1, Table S2) were transformed into *E. coli* CA434 strain and subsequently used to conjugate sequence-confirmed plasmids into R20291$_{CM196}$ *C. difficile* strain to insert into *pyrE* locus by allelic exchange using *pyrE* allele [45]. Transformed *E. coli* CA434 strains were mated with *C. difficile* R20291$_{CM196}$ *ΔpyrE* [45]. Transconjugants were selected by subculturing on BHIS agar containing 15 µg/mL thiamphenicol, 16 µg/mL cefoxitin and 250 µg/mL cycloserine and re-streaked five times to select for fast growing colonies. Transconjugants were then streaked onto CDMM with 1.5% (w/v) agar to select for plasmid excision and restoration of *pyrE* locus [44-46]. Replicate plating on BHIS and BHIS plates containing 15 µg/mL thiamphenicol was done to select transconjugants without thiamphenicol resistance to ensure resistance cassette from plasmid is not being expressed. Colonies sensitive to thiamphenicol were screened by PCR to confirm restoration of *pyrE* and insertion of spore surface reporter system into loci by using primers p921 (FP pyrD) and p924 (RP 0190), and insertion of spore surface reporter construct using primers p921 (FP pyrD) and p1076 (RP Nluc-HindIII). Growth curve analyses were performed to select strains that grew like wild type strain. Whole-genome sequencing was conducted on all mutant strains using Illumina sequencing by SeqCenter (Pittsburgh, PA) to confirm the absence of off-target mutations and validate the intended genetic modifications.

Conjugation of plasmids carrying spore surface reporter constructs carrying *48aa-ntd*$_{bclA1}$*-nLuc* or *193aa-ntd*$_{bclA1}$*-nLuc* under the control of *cdeC* native promoter (pMTL-YN2C-TT-P_{cdeC}-*48aa-ntd*$_{bclA1}$*-nLuc*, and pMTL-YN2C-TT-P_{cdeC}-*193aa-ntd*$_{bclA1}$*-nLuc*) was performed as described above for R20291$_{CM196}$ Δ*pyrE*, with identical selection, counter-selection, and verification steps. Briefly,

transconjugants were isolated on BHIS with 15 µg/mL thiamphenicol, 16 µg/mL cefoxitin, and 250 µg/mL cycloserine, then processed through single-crossover isolation, plasmid excision, selection of *pyrE* restoration on CDMM with 1.5% agar, thiamphenicol-sensitivity screening, locus confirmation by PCR (p921 and p924 for *pyrE* restoration and insertion; p921 and p1076 for *nLu*c fusion), growth-curve assessment, and whole-genome sequencing validation.

Fluorescent reporter strains were generated using the same conjugation and allelic exchange workflow described above: pMTL-YN2C-TT-P_{cdeC}-*48aa-ntd*$_{bclA1}$*-mNeonGreen* and pMTL-YN2C-TT-P_{cdeC}-*193aa-ntd*$_{bclA1}$*-mNeonGreen* were transferred from *E. coli* CA434 into *C. difficile* R20291$_{CM196}$ Δ*pyrE*, and pMTL-YN2C-TT-P_{cdeC}-*48aa-ntd*$_{bclA1}$*-mScarlet-i3* and pMTL-YN2C-TT-P_{cdeC}-*193aa-ntd*$_{bclA1}$*-mScarlet-i3* were transferred into R20291$_{CM210}$ Δ*pyrE*. Transconjugants were isolated on BHIS with 15 µg/mL thiamphenicol, 16 µg/mL cefoxitin, and 250 µg/mL cycloserine, then processed through single-crossover isolation, plasmid excision, selection of *pyrE* restoration on CDMM with 1.5% agar, thiamphenicol-sensitivity screening, locus confirmation by PCR (p921 and p924 for *pyrE* restoration and insertion; p921 and p1360 for *mNeonGreen* fusion or p1523 for *mScarlet-i3* fusion), growth-curve assessment, and whole-genome sequencing validation as described for the NanoLuc constructs. To make sure insertions did not resulted in mutations leading to growth deficits growth curves were performed before sequencing.

C. difficile growth curves

For growth curves, BHIS plates were prepared and pre-reduced in COY anaerobic chamber. Frozen stocks of strains were streaked onto plates and incubated at 37 °C for 16 h. Single colonies were inoculated into prewarmed BHIS broth and grown overnight at 37 °C. For growth curve analysis, overnight cultures were diluted 1:100 into fresh BHIS supplemented with 0.5% (w/v) glucose and 0.1% (w/v) sodium taurocholate medium and incubated at 37 °C for 8 h. These cultures were further diluted 1:100 into BHIS broth, and 100 µL aliquots were transferred to a 96-well microplate in triplicate. The plate was loaded into a Cerillo Alto microplate reader, and optical density (OD) measurements were recorded at 600 nm over 20 h under anaerobic conditions.

Sporulation efficiency using phase-contrast microscopy

To assess sporulation efficiency using phase-contrast microscopy, frozen stocks of *C. difficile* strains were streaked on BHIS plates and incubated for 16 h at 37 °C under anaerobic conditions. Next day, we inoculated BHIS broth with single colony and incubated overnight at 37 °C. Then overnight culture was diluted 1:100 and incubated 5 h at 37 °C. 100 µL of 5 h culture was spread on 70:30 sporulation medium plates and incubated for 16 h under strict anaerobic conditions. After incubation, cultures were harvested and resuspended in 1 mL 1X Phosphate buffered saline (PBS). Then, 10 µL were then mounted on 1% agarose pads onto glass slides for phase-contrast microscopy. Using a 100x oil-immersion objective, we captured images from multiple random fields of view for each sample. Cells were classified into three categories based on their morphology and phase-brightness: vegetative cells (phase-dark rods), sporulating cells (phase-dark mother cells containing phase-bright forespores), and mature spores (distinct phase-bright, oval structures). We manually counted at least 300 cells per strain, tallying the numbers in each

category. Sporulation efficiency was calculated as the percentage of cells from each stage out of the total counted population.

Purification of *C. difficile* spores

C. difficile strains were cultured anaerobically at 37 °C in Brain Heart Infusion broth (BHIS; Difco) supplemented with 0.1% sodium taurocholate and 0.2% d-fructose to promote germination. To ensure cultures are actively growing in mid-exponential phase, OD_{600} was measured and cultures were diluted to an OD600 < 0.9, incubated for 90 min at 37 °C before spreading 250 µL onto 70:30 sporulation medium [18]. Plates were then incubated under anaerobic conditions for 5 days at 37 °C and harvested by scrapping plate, resuspending in sterile ice-cold Milli-Q dH_2O water, and incubating overnight at 4 °C to allow vegetative cells lysis and spore release. Following incubation, spores were pelleted down by centrifugation and pellets were washed with sterile ice-cold Milli-Q dH_2O water to remove debris followed by centrifugation at 13,000 g for 5 min. Washes with sterile ice-cold Milli-Q dH_2O water were repeated until spore purity reached 99%. To assess purity, spores were observed on 1% agarose pad and considered pure when sample was free of vegetative cells and/or debris. Purified spores were quantified using a Neubauer chamber and stored in 50 µL aliquots at -80 °C at a concentration of 5 x 10^9 spores/mL until use.

Bioluminescence assays with purified spores

Purified spores were diluted with PBS to a desired concentration of $1x10^9$ spores/mL, unless indicated otherwise, and 50 µL of the diluted spores were transferred to a white 96-well plate in triplicate. The Nano-Glo Luciferase Assay Reagent (Promega N1110) was reconstituted by combining one volume of Nano-Glo Luciferase Assay Substrate with 50 volumes of Nano-Glo

Luciferase Assay Buffer (Promega N1110) Then, 50 μL of Nano-Glo reconstituted reagent was added to each well. Bioluminescence signals were immediately recorded for 30 min using a BioTek Synergy H1 microplate reader at an emission wavelength of 460 nm following reagent addition.

Dynamic range of purified spores was assessed by diluting with PBS to $2x10^8$ spores/mL and serial diluted 6 times. 50 μL of the diluted spores from each dilution were transferred to a white 96-well plate in triplicate. The Nano-Glo Luciferase Assay Reagent (Promega) was reconstituted by combining one volume of Nano-Glo Luciferase Assay Substrate with 50 volumes of Nano-Glo Luciferase Assay Buffer. Then, 50 μL of Nano-Glo reconstituted reagent was added to each well. Bioluminescence signals were immediately recorded for 30 min using a BioTek Synergy H1 microplate reader (Agilent) at an emission wavelength of 460 nm following reagent addition.

Nano-Glo reagent titration

Purified spores were diluted with PBS to $1x10^9$ spores/mL and 50 μL of the diluted spores were transferred to a white 96-well plate in triplicate. The Nano-Glo Luciferase Assay Reagent (Promega) was reconstituted by combining Nano-Glo Luciferase Assay Substrate and Nano-Glo Luciferase Assay Buffer at following dilution ratios: 1:50, 1:75, 1:100, 1:250, 1:500, 1:1,000, 1:5,000, 1:10,000, 1:50,000 and 1:100,000 (substrate: buffer). Subsequently, 50 μL of Nano-Glo reconstituted reagent was added to each well. Bioluminescence signals were immediately recorded for 30 min using a BioTek Synergy H1 microplate reader at an emission wavelength of 460 nm following reagent addition.

Dynamic range of bioluminescence of spores in fecal samples

Fresh fecal samples (~25mg) were weighed in pre-weighed tubes to determine sample mass. Feces were resuspended in PBS to a 4% (w/v) concentration. Spore dilutions were prepared in PBS. To prepare fecal-spore mixtures, 60 µL of spore dilution was added to 240 µL of fecal suspension, yielding a final fecal concentration of 3.2% (w/v); these samples were designated as undiluted fecal samples. Before bioluminescence measurement, 50 µL of the undiluted fecal-spore mixture was transferred in duplicate to white, flat-bottom 96-well microplates. Serial dilutions were prepared from the undiluted fecal-spore samples: a 1:10 dilution was made by transferring 15 µL of undiluted sample into 135 µL PBS, and a 1:50 dilution by transferring 10 µL of undiluted sample into 490 µL PBS. From each dilution, 50 µL was loaded in duplicate into wells of the 96-well plate. Next, Nano-Glo Luciferase Assay Reagent (Promega) was prepared at a 1:100 substrate-to-buffer ratio according to prior titration experiment. Subsequently, 50 µL of 1:100 diluted Nano-Glo reagent was added to each well and mixed thoroughly. Bioluminescence signals were immediately recorded for 30 min using a BioTek Synergy H1 (Agilent, USA) microplate reader at an emission wavelength of 460 nm following reagent addition at room temperature.

Transmission Electron Microscopy

C. difficile spores were processed using modified established protocols [48,49]. Spores were fixed overnight at 4 °C in 3% glutaraldehyde with 0.1 M cacodylate buffer (pH 7.2), centrifuged, and post-fixed in 1% osmium tetroxide (0.05 M HEPES, pH 7.4) overnight at 4°C. After five distilled water washes, samples were dehydrated through a graded acetone series (30%, 50%, 70%, 90% for 15 min each) followed by three 30 min incubations in 100% acetone, retaining residual acetone to prevent rehydration. Embedding was performed in modified Spurr's resin (Quetol ERL 4221)

using a Pelco Biowave processor under alternating vacuum conditions: 1:1 acetone-resin mixtures (10 min without vacuum, 5 min with vacuum) followed by four 5 min pure resin exchanges. Resin-infiltrated pellets were transferred to BEEM capsules, polymerized at 65°C for 48 h, and cured at room temperature for 24 h. Ultrathin sections (~100 nm) were cut on a Leica UC7 ultramicrotome, mounted on glow-discharged 400 mesh carbon-coated copper grids, and double-stained with 2% uranyl acetate (5 min) and Reynold's lead citrate (5 min), with sterile MilliQ water washes between steps in falcon tubes using dipping motion. Imaging was conducted on a JEOL 1200 EX TEM at 100 kV, with micrographs captured using an SIA-15C CCD camera at 2,721 × 3,233 pixel resolution via MaxImDL software. All equipment used is located at the Texas A&M University Microscopy and Imaging Center Core Facility (RRID: SCR_022128).

Western blotting

20 µL of purified spores ($1x10^8$) were pellet down, resuspended in 40 µL of USD buffer (8M Urea, 1% (w/v) SDS, 50 mM DTT, 50 mM Tris-HCl pH 8) and incubated for 90 min at 37 °C [50]. After incubation, samples were pellet down and supernatant was transferred to new microcentrifuge tube. Supernatant was mixed with 2X SDS-PAGE sample loading buffer (Bio-Rad), boiled for five minutes, and electrophoresed on 12% acrylamide gels along PageRuler Plus prestained protein ladder (Thermo Fischer). Proteins were transferred to nitrocellulose membranes (Bio-Rad) and blocked for 1 h at room temperature with 3% BSA in 0.1% Tween-20 Tris-buffered saline (T-TBS). Membranes were then probed with 1:1,000 mouse anti-NanoLuc antibody (Promega N700) in 1% BSA in T-TBS overnight at 4 °C or with 1:10,000 rabbit anti-SleC in 1% BSA in T-TBS for 1 h at room temperature. Anti-SleC was a gift from Dr. Joseph Sorg at Texas A&M University [51]. After rinsing, membranes were incubated for 3 h at room temperature with 1:10,000 goat anti-

rabbit IgG HRP (Thermo 31460) or 1:10,000 goat anti-mouse IgG HRP (Sigma A5278) in 1% BSA in T-TBS. Detection was performed using chemiluminescent substrate (Bio-Rad) and visualized with Li-Cor C-Digit Blot Scanner according to the manufacturer's instructions.

Murine model of CDI

Male and female C57BL/6 mice (6-8 weeks old) were obtained from an established breeding colony in the Biology Department Animal Facility at Texas A&M University, originally derived from Jackson Laboratories. Animals were maintained under controlled conditions with a 12 h light/dark cycle at 20-24 °C and 40-60% relative humidity and provided ad libitum access to autoclaved food and water. Bedding and cages were sterilized by autoclaving prior to use. All animal procedures were conducted in accordance with institutional and national guidelines for animal care and use and were approved by the Institutional Animal Care and Use Committee (IACUC) of Texas A&M University.

Mice received an antibiotic cocktail via drinking water *ad libitum* for 3 days, delivering approximately 4.2 mg/kg/day kanamycin, 3.5 mg/kg/day gentamicin, 4.2 mg/kg/day colistin, 21.5 mg/kg/day metronidazole, and vancomycin 4.5 mg/kg/day, calculated from average water intake and mouse body weight [11,14,52]. Two days following completion of antibiotic treatment, mice received a single100μL intraperitoneal injection of 10 mg/kg clindamycin (Sigma-Aldrich) [14]. One day post-clindamycin, mice were orally gavage with 1×10^5 *C. difficile* spores i) *C. difficile* R20291$_{CM210}$ WT strain (WT), ii) with CM210 R20291 *193aa-ntd$_{bclA1}$-nLuc* strain (*nLuc*). , or iii) PBS-saline (Mock), using a 23-gauge steel ball-tipped gavage needle attached to a 1 mL syringe. Animals were housed individually in sterile cages to prevent cross-contaminations with *ad libitum*

access to food and water. All procedures and handling were performed aseptically in a biosafety cabinet. The biosafety cabinet and gloves were disinfected between cage manipulation using sequential treatment with 5% (v/v) bleach solution followed by 70% (v/v) ethanol solution to prevent cross-contamination.

Following inoculation, mice were monitored for six days to assess development and resolution of disease, weight loss, diarrhea, vegetative cell and spore load in feces, and bioluminescence in fecal and cecal samples. Vegetative cell and spore colony forming units in feces was quantified daily by plating homogenized fecal samples onto agar plates with medium supplemented with 16 µg/mL cefoxitin and 250 µg/mL L-cycloserine (CCFA) for vegetative cells or medium supplemented with 0.1% (w/v) sodium taurocholate, 16 µg/mL cefoxitin and 250 µg/mL L-cycloserine (TCCFA) for spores [52,53]. Diarrhea severity was scored based on stool consistency and appearance as follows: normal stool (score = 1), color change or altered consistency (score = 2), presence of wet tail or mucosa (score = 3), and liquid stools (score = 4). Scores greater than 1 were classified as diarrhea [54]. Additional clinical indices recorded daily included body weight, physical appearance (e.g., hunched posture, piloerection), spontaneous behaviors (lethargy, inactivity, or immobility), and signs of emaciation, monitored as previously described [11,14,52].

Mice reaching moribund status, defined as loss of 20% of initial body weight in combination with inactivity, diarrhea, or a poor fur coat, were humanely euthanized [55,56]. Remaining mice were euthanized at the study endpoint. At day 3 post-infection mice were humanely euthanized via isoflurane overdose followed by cervical dislocation or thoracotomy, in accordance with American

Veterinary Medical Association guidelines. Post-mortem, cecal content was collected for quantification of *C. difficile* vegetative cell and spore load.

Quantification of *C. difficile* vegetative cells and spores from feces and cecum

Feces and cecal samples were processed using modified established protocols [52]. Fecal pellets were collected daily, weighted in pre-weighted tubes and immediately transferred to anaerobic chamber to then be hydrated with 500 µL of sterile pre-reduced PBS, followed by incubation at room temperature for 30 min. To quantify vegetative cell colony forming units, samples were serially diluted and plated onto CCFA plates. For quantification of shed spores, hydrated fecal samples were diluted 1:1 with absolute ethanol and incubated for 1 h at room temperature. Samples were serially diluted and plated onto selective medium supplemented with 0.1% (w/v) sodium taurocholate, 16 µg/mL cefoxitin and 250 µg/mL L-cycloserine (TCCFA plates). The plates were incubated anaerobically at 37 °C for 48 h, colonies counted, and results expressed as Log_{10} of colony forming units (CFU) per gram of feces. Cecal content was collected post-euthanasia, weighted in pre-weighted tubes and processed as fecal samples for determination of vegetative cell and spore load.

Bioluminescence from feces and cecum

Previously hydrated fecal and cecal samples were diluted 1:10 in sterile PBS. From these dilutions, 50 µL aliquots were transferred in triplicate to a white 96-well plate. The Nano-Glo® Luciferase Assay Reagent (Promega) was prepared at a 1:100 substrate-to-buffer ratio according to prior titration experiment. Subsequently, 50 µL of the Nano-Glo® reagent was added to each well.

Bioluminescence signals were immediately recorded for 30 min using a BioTek Synergy H1 microplate reader at an emission wavelength of 460 nm following reagent addition.

Daily thresholds for positive signal determination were defined based on mock (negative control) samples. Threshold was calculated as the mean (μ) and standard deviation (σ) of bioluminescence readings in mock animals' fecal samples, and defined bioluminescence positive threshold as $\mu + 2.81 \times \sigma$. Measurements exceeding this threshold were categorized as positive.

PCR ribotyping and confirmation of *nLuc* fragment in stool-derived strains

Genomic DNA (gDNA) was extracted from *C. difficile* single colonies grown on CCFA plates using a phenol-chloroform extraction method. PCR ribotyping was performed by amplifying the intergenic spacer region between the 16S and 23S rRNA genes [57]. PCR reactions (10 µL) contained 1× ThermoPol reaction buffer, 200 µM dNTPs, 0.2 µM of primers 16S rRNA and 23 rRNA (see Supplementary Table 2), 1.25 U Taq DNA polymerase, and approximately 50 ng template gDNA (New England Biolabs). Cycling conditions were 95 °C for 5 min; 35 cycles of 95 °C for 1 min, 53 °C for 1 min, and 72 °C for 1 min; followed by a final extension at 72 °C for 10 min. PCR products were resolved on 1% agarose gels stained with ethidium bromide and visualized under UV light.

PCR amplification to confirm the presence of the *nLuc*-containing fragment was performed in 25 µL PCR reactions containing 1× ThermoPol reaction buffer, 200 µM dNTPs, 0.2 µM primers (FP pyrE det and RP-NTD_BclA1_193aa_NcoI-GC or RP NLuc-HindIII see Supplementary Table 2), 1.25 U Taq DNA polymerase, and approximately 50 ng template gDNA (New England Biolabs).

Thermal cycling conditions consisted of an initial denaturation at 95 °C for 30 s; 30 cycles of 95 °C for 30 s, annealing at 55 °C for 30 s, extension at 68 °C for 1 min; and a final extension at 68 °C for 5 min. PCR products were visualized by 1% agarose gel electrophoresis with ethidium bromide staining under UV illumination.

Fluorescent Microscopy of fluorescent spore surface reporter strains

Frozen *C. difficile* stocks were streaked onto BHIS agar plates and incubate anaerobically at 37 °C overnight. Next, a single colony was selected to inoculate 5 mL of BHIS broth and incubated anaerobically at 37 °C for 16 h. The following day, 500 µL of the overnight culture was transferred to 5 mL BHIS supplemented with 0.1% (w/v) sodium taurocholate and 0.5% (w/v) glucose, followed by incubation at 37 °C for 5 h to sync exponential growth between strains. Subsequently, 250 µL of the culture was evenly spread onto 70:30 sporulation agar plates, which were incubated anaerobically at 37 °C for 16, 24 or 48 h to induce sporulation. Sporulating cultures were harvested from single 70:30 plate and resuspended in 1 mL PBS followed by three washes with PBS using gentle centrifugation at 6,000 × g for 5 min. For membrane staining, cell pellets were resuspended in 450 µL PBS and stained with 50 µL of membrane dye MTG was added (1 µL of 1 mg/mL stock in 999 µL PBS) followed by three additional PBS washes.

Sporulating cultures were fixed by resuspending the pellet in 500 µL PBS and fixed by addition of 120 µL of fixation cocktail (prepared from 100 µL 16% (w/v) paraformaldehyde and 20 µL 1 M sodium phosphate buffer, pH 7.4). Sporulating cells were fixed in two steps; a first fixation performed aerobically in the dark for 30 min at room temperature, followed by 30 min on ice [43]. Fixed cells were washed three times with PBS before resuspending in 1 mL PBS containing 1 µL

of 1 mg/mL DAPI. After incubation in the dark at room temperature for 2 min, cell pellets were washed three times with PBS and resuspended in 200 µL PBS for imaging. Next, 10 µL aliquots of fixed samples were transferred to agarose pads. Imaging was performed using a Leica DMRX fluorescence microscope equipped with a Hamamatsu C8484 Digital Camera. Phase-contrast images (2546 × 2546 pixels) were acquired with a 150 ms exposure. Fluorescent images (2546 × 2546 pixels) were captured using an mCherry filter set (excitation in red channel, 1 s exposure) and a FITC filter set (green channel, 1.5 s exposure).

Fluorescence micrographs were analyzed using Fiji (ImageJ) by segmentation of individual cells corresponding to distinct developmental stages was accomplished manually using the Freehand Selection tool to define regions of interest (ROIs) encompassing each cell [58]. The raw integrated density and cell area were measured using the Analyze > Measure function with "Area" and "Raw Integrated Density" options enabled. For every cell, raw integrated density and area measurements were exported. Fluorescence intensities were then normalized to cell area (Raw Integrated Density/Area) in Microsoft Excel to enable direct quantitative comparison across cells or experimental groups.

Imaging of fluorescent spore surface reporter strains during CDI

Mice were pretreated as previously described with an antibiotic cocktail in drinking water for 3 days and clindamycin pre-infection. One day post-clindamycin, mice were orally gavaged with 1 × 10^5 *C. difficile* spores i) *C. difficile* R20291$_{CM210}$ WT strain (WT), ii) with R20291$_{CM210}$ *193aa-ntd$_{bclA1}$-mScarlet-i3* (*mScarlet-i3*). , or iii) R20291$_{CM196}$ *193aa-ntd$_{bclA1}$-mNeonGreen* strain (*mNeonGreen*), using a 23-gauge steel ball-tipped gavage needle attached to a 1 mL syringe.

Animals were housed individually in sterile cages to prevent cross-contaminations with *ad libitum* access to food and water. All procedures and handling were performed aseptically in a biosafety cabinet. The biosafety cabinet and gloves were disinfected between cage manipulation using sequential treatment with 5% (v/v) bleach solution followed by 70% (v/v) ethanol solution to prevent cross-contamination.

Mice reaching moribund status, defined as loss of 20% of initial body weight in combination with inactivity, diarrhea, or a poor fur coat, were humanely euthanized [55,56]. Remaining mice were euthanized at the study endpoint. At day 3 post-infection mice were humanely euthanized via isoflurane overdose followed by cervical dislocation or thoracotomy, in accordance with American Veterinary Medical Association guidelines. Post-mortem, colon and cecum tissue was collected immunostaining and confocal imagining.

Tissue Fixation, Immunofluorescence Staining and Confocal Imaging

On day 3 post-infection, colonic and cecal tissues were harvested from *C. difficile* infected mice and immediately processed for immunofluorescence. Tissues were fixed flat in 4% paraformaldehyde containing 30% sucrose and incubated overnight at 4 °C in the same solution. Samples were then washed five times with PBS and permeabilized for 2 h in PBS containing 0.2% Triton X-100. Following permeabilization, tissues were washed five additional times in PBS and blocked for 3 h at room temperature in PBS supplemented with 3% BSA.

Primary antibody staining was performed by incubating tissues overnight at 4 °C with 1:500 chicken anti-spore antibody (Aves Lab 7246) and 1:200 phalloidin AF-647 (Abcam AB176759)

diluted in PBS containing 3% BSA. After washing five times in PBS, tissues were incubated with 1:400 goat anti-chicken AF-488 secondary antibody (Abcam ab150175) or 1:400 goat anti-chicken AF-568 secondary antibody (Abcam 175711) and 1:1000 DAPI (Thermo D21490) for 3 h at room temperature. Samples were then washed five times with PBS, mounted apical side up with ProLong™ Diamond Antifade Mountant (5 µL below and 15 µL above the tissue), and sealed with tape. All incubations and washes performed at room temperature were carried out on a shaker at 90 rpm. All washes were performed in 1 mL PBS, and antibody incubations were performed in 200 µL volume.

Imaging was performed in a Stellaris 5 confocal on a DMI-8 stage using a HC PL FLUOTAR 63x/1.10 IMM objective to quantify *C. difficile* spores tagged with *mNeonGreen* or *mScarlet-i3*. Non-infected tissues from mock mice were included to assess and calibrate background signal. Navigator scans were first generated to survey ~0.11 cm^2 of tissue area (~360 images; field of view 184 µm x 184 µm per image) to identify spore-enriched regions. The entire tissue was screened until at least two spore-enriched areas were located and imaged using four adjacent Z-stacks per region. Confocal stacks were collected from the tissue surface to a depth of 25 µm using 100 serial steps at a 0.25 µm step size. Imaging parameters were standardized using counting mode to ensure consistent laser intensity and gain (2.5% or 17.6% gain, 1% laser intensity). Images were acquired at 2160 x 2160 pixel resolution for single images and 1024 x 1024 pixel resolution for Z-stacks, with a line average of 2 and scan line speed of 600 Hertz. The pinhole size was set to 1.

Confocal image stacks were analyzed using LAS X 3D Analysis software (Leica Microsystems, Germany), and spores were detected in either the green (488 nm excitation) or red (567 nm excitation) spectral channels. Detected objects within 5-10 nm were then reviewed and curated to classify spores based on total spore labeling and reporter fluorescence in each strain. For the *193aa-ntd*$_{bclA1}$*-mNeonGreen* strain, total spores were identified using anti-spore antibody staining detected in the red channel (567 nm excitation), while *mNeonGreen*-positive spores were detected in the green channel (488 nm excitation). Thus, spores were categorized as: (i) total spores (anti-spore positive) and (ii) fluorescent spores (*mNeonGreen*-positive), with fluorescence assessed relative to the total spore population in each tissue region. For the *193aa-ntd*$_{bclA1}$*-mScarlet-i3*strain, the channel assignment was inverted: total spores were detected using anti-spore antibody in the green channel (488 nm excitation), and *mScarlet-i3* fluorescence was detected in the red channel (567 nm excitation). Spores were similarly classified as total spores and fluorescent spores, and the proportion of reporter-positive spores was calculated by dividing the number of fluorescent spores by the total number of anti-spore-labeled spores in the corresponding tissue section.

PCR ribotyping and confirmation of *mScarlet-i3* or *mNeonGreen* fragment in stool-derived strains

Genomic DNA (gDNA) was extracted from *C. difficile* single colonies grown on CCFA plates using a phenol-chloroform extraction method. PCR ribotyping was performed as before by amplifying the intergenic spacer region between the 16S and 23S rRNA genes [57]. PCR amplification to confirm the presence of the *mScarlet-i3* or *mNeonGreen* fragment was performed in 25 µL PCR reactions containing 1× ThermoPol reaction buffer, 200 µM dNTPs, 0.2 µM of

primers p947 (FP pyrE det) and p1523 (RP PcdeC-NTD-BclA1 for mScarlet i3) for *mScarlet-i3* and p1360 (RP PcdeC-NTD-BclA1 for NeonGreen) for *mNeonGreen*, 1.25 U Taq DNA polymerase, and approximately 50 ng template gDNA (New England Biolabs). Thermal cycling conditions consisted of an initial denaturation at 95 °C for 30 s; 30 cycles of 95 °C for 30 s, annealing at 55 °C for 30 s, extension at 68 °C for 1 min; and a final extension at 68 °C for 5 min. PCR products were visualized by 1% agarose gel electrophoresis with ethidium bromide staining under UV illumination.

Statistical Analysis

Statistical analyses were performed using GraphPad Prism 10. For *in vitro* experiments, differences between groups were assessed by ordinary one-way ANOVA assuming Gaussian distribution, followed by Šídák's multiple comparisons test. For dynamic range with purified spores, differences were assessed by Welch and Brown-Forsythe ANOVA, assuming Gaussian distribution with Dunnett's multiple comparisons test. For dynamic range in fecal matter, differences were assessed by ordinary two-way ANOVA with Tukey's multiple comparisons test. For *in vivo* experiments, mixed-effect analysis with Tukey's multiple comparisons test was applied. For fluorescence distribution in fluorescent reporters' samples, normality was evaluated with the D'Agostino-Pearson omnibus test and differences with two tailed unpaired Welch's t-test. Group differences for tissue-associated fluorescent reporter spore detection was done using RM one-way ANOVA with Geisser-Greenhouse correction. Significance levels are indicated as follows: $P < 0.05$ (*), $P < 0.01$ (**), $P < 0.001$ (***), and $P < 0.0001$ (****). Where applicable, groups sharing the same letter are not significantly different ($P \geq 0.05$) according to pairwise comparisons; different letters indicate significant differences.

S2 Extended Results - Fluorescent reporter data analysis

S2.1 *mScarlet-i3* is expressed by sporulating cells but poorly retained in mature spores.

To expand the utility of the $P_{cdeC\_}ntd_{bclA1}$ as a spore-surface anchor, the fluorescent reporters *mScarlet-i3* and *mNeonGreen* were fused to either the 48-aa- or 193-aa- ntd_{bclA1}, and subsequently integrated at the *pyrE* locus of $R20291_{CM210}$ or $R20291_{CM196}$ strains (Fig 7A, Fig. S13, Fig S14, Fig. S15, Fig. S16A) [43, 78]. Sporulating cultures were stained for MTG and DAPI to define the developmental stages and analyzed by fluorescent microscopy. Results show that *48aa-* and *193aa-*ntd_{bclA1}*-mScarlet-i3* strains lacked detectable red signal at stages I-II but displayed fluorescence from stage III onward, with highest intensities observed in late sporulating cells (Fig. 7B, Fig. S17B-C). In free and purified spores, only a subset of spores retained visible red signal, whereas wild type cells showed only low background in the red channel (Fig. 7B, Fig. S17). In contrast, expression of *mNeonGreen* fusions was indistinguishable from the intrinsic *C. difficile* autofluorescence in all analyzed stages (Fig. S16B). In summary, while *mScarlet-i3* fluorescence was visible during late sporulation and a subset of spores, *mNeonGreen* are masked by *C. difficile* autofluorescence.

Since micrographs revealed that only a subset of cells displayed fluorescence, single-cell analysis was used to quantify fluorescence intensity across developmental stages (Fig. 7C-D, S16C-D). Similar fluorescence was observed between 48-aa-, 193-aa- *mScarlet-i3* fusions, and wild type at stages I-II (e.g., ~4,000 to 5,023 a.u.) (Fig. 7C). However, during Stage III, the 48-aa-fusion increased intensity to 7,800 a.u., significantly higher than the 193-aa-fusion (e.g., 5,600

a.u.) (Fig. 7C). This difference between fusions persisted throughout Stage IV and V, with *48aa-ntd*$_{bclA1}$ having ~ 40% higher fluorescence than *193aa-ntd*$_{bclA1}$ (Fig. 7C). A significant decrease in fluorescence intensity of ~39 % was observed in free spores from a 16 and 48 h sporulating cultures for both constructs (Fig. 7C). Purified spores exhibited even lower fluorescence (~3,600 vs. ~3,400 a.u.), with the 48-aa construct remaining slightly brighter (Fig. 7C-D). Wild type sporulating cells remained at low background levels during all stages (Fig. 7C-D). Collectively, these results demonstrate that, in contrast to the higher bioluminescence observed for *193aa-ntd*$_{bclA1}$, the 48-aa-fusion had higher *mScarlet-i3* fluorescence than the 193-aa-fusion. Importantly, results indicate that although *mScarlet-i3* reporter was expressed in the mother cell, its localization and retention to the spore surface was less efficient.

mNeonGreen reporter was similarly quantified by single-cell fluorescence across developmental stages (Fig. S16C-D). Mean green fluorescence for the 48-aa and 193-aa fusions were high at stage I ~13,000-15,000 a.u., remained in a similar 10,000-14,000 a.u. range through stages II-V, and decreased only modestly in free and purified spores (Fig. S16C-D). Across all stages, wild type cells and spores exhibited green fluorescence that frequently matched or exceeded these values (Fig. S16C-D). In summary, intrinsic green autofluorescence in *C. difficile* therefore masks reporter-derived signal from *mNeonGreen* at all developmental stages *in vitro*.

S2.2 *mScarlet-i3* tagging is restricted to a subpopulation of *C. difficile* cells.

Since single-cell analysis revealed broad distributions of fluorescence intensities. Therefore, to gain more insight into fluorescence heterogeneity, *mScarlet-i3* data for each fusion strain were re-plotted by developmental stage (Fig. S18, Fig. S19). We observed that for both, 48-aa- and 193-

aa-fusion, the mean intensity increased modestly from stages I-II, rose significantly at stages III-IV, and then declined at stage V and in free/purified spores (Fig. S18C, E). Importantly, we observed high heterogeneity in the fluorescence intensity in all stages for both constructs, which could be classified into low, mid and high (Fig. S18D and S18F). Similar pattern was observed for in free spores from 16 and 48 h sporulating cultures and in free spores from both constructs (Fig. S20C, E, Fig. S20A-C). These data show pronounced intra-population variability in *mScarlet-i3* reporter expression and suggests that only a subset of cells and spores accumulate detectable levels of the fluorescent reporter.

To investigate the observed heterogeneity, fluorescence intensity distributions for each developmental stage and fusion were tested for normality (Fig. S20A-H). Many stages, particularly stages I, IV, V, free spores at 16 h and purified spores, showed significant deviations from a normal distribution consistent with distinct subpopulations rather than a single homogeneous population. A fluorescence threshold was then defined for each experiment as the wild type mean (μ) plus 2.576 standard deviations (σ), giving 99.5% confidence interval, and was applied to classify the cells and spores into *mScarlet-i3*-positive and -negative cells (Fig. S20I-P). For the 48-aa fusion, cell populations above and below threshold were statistically distinct at stages III-V and in free spores (Fig. S20I-L). The 193-aa-fusion showed a smaller positive subset at stages III-V and free spores with most cells below threshold (Fig. S20M-P). These analyses indicate marked, and in some stages apparently bimodal, fluorescence heterogeneity during late sporulation, with released spores displaying weaker but clearly heterogeneous fluorescence confined to a subset of the population.

S2.3 *In vivo* detection of fluorescently tagged *C. difficile* spores in colon and cecum

mNeonGreen has an excitation and emission of 506 and 517 nm [79], respectively, which overlaps with *C. difficile* autofluorescence excitation and emission [80]. Therefore, 488 nm laser specific excitation and a narrow detection window of 500 to 550 nm could yield detection of *mNeonGreen*-tagged spores during an infection in a murine model of CDI. Moreover, we also tested whether *mScarlet-i3*-specific excitation laser (e.g. 567 nm) and a detection window of 580 to 630 nm, would allow detection of *mScarlet-i3*-tagged spores. For this, antibiotic-treated mice were infected with wild-type, *193aa-ntd*$_{bclA1}$*-mNeonGreen* , and *193aa-ntd*$_{bclA1}$*-mScarlet-i3* strain. During peak of infection (day 3), mice were sacrificed, and cecum and colon tissue were stained for total spores with anti-spore chicken antibody as a control for total spores [11, 81] (Fig. S21A). Ribotyping and reporter-specific PCR of stool-isolated colonies collected from infected mice confirmed that recovered strains matched the inoculated ribotype and had the *mScarlet-i3* or *mNeonGreen* fusions (Fig. S22). Interestingly, for the *193aa-ntd*$_{bclA1}$*-mScarlet-i3* strain, an average of ~ 141 and 104 spores per 3.40×10^4 µm^2 were detected with anti-spore chicken antibody in colon and cecum (Fig. 7E). From a total of 2,389 spores in colonic tissue, only ~13.4% of *C. difficile* spores had detectable red fluorescence (Fig. 7E-F). By contrast, no *mScarlet-i3*-specific fluorescence was observed in a total of 1,346 spores detected in cecum (Fig. 7F). For the *mNeonGreen* strain, an average of ~887 and 147 spores per 3.40×10^4 µm^2 were detected with anti-spore chicken antibody in colon and cecum (Fig. 7G). From a total of 9,755 spores in colonic tissue, none had detectable green fluorescence (Fig. 7H). By contrast, ~13.9% of cecal spores had detectable green fluorescence (Fig. 7G). A noteworthy aspect was that fluorescence-based spore detection *in vivo* seems to be rare and site dependent. While *mScarlet-i3* labeled only colonic spores, *mNeonGreen* labeled only cecum spores. Overall, these results provide insight on how *C. difficile*

spores are tagged and imaged *in vivo*, highlighting substantial limitations and improvements of both reporters for visualizing *C. difficile* spores *in vivo*.
