# Supplementary material for: Development and Evaluation of Exosporium-Anchored Bioluminescent and Fluorescent Reporters for Tracking Clostridioides difficile Spores Formed In Vivo
Source: ACS Synth Biol. 2026 May 15;15(6):2338–55. doi: 10.1021/acssynbio.5c00961 (PMC13288923; doi:10.1021/acssynbio.5c00961)
Supplement: Supplementary file 4 [file sb5c00961_si_004.pdf]

# TABLE S1. Bacterial strains used

| Strain | Relevant characteristic | Source/Reference |
|---|---|---|
| *E. coli NEB Turbo* | *F' $proA^{+}B^{+}$ $lacI^{q}$ ΔlacZM15 / fhuA2 Δ(lac-proAB) glnV galK16 galE15 R(zgb-210::Tn10)$Tet^{S}$ endA1 thi-1 Δ(hsdS-mcrB)5* | New England Biolabs |
| *E. coli* CA434 | *hsd20*(rB-, mB-, *recA13*, *rpsL20*, *leu*, *proA2*, with IncPb conjugative plasmid R702 | [1] |
| *C. difficile* $R20291_{CM210}$ | Ribotype 027, epidemically relevant strain | [2] |
| *C. difficile* $R20291_{CM210}$ Δ*pyrE* | R20291 isogenic *pyrE* mutant | [3, 4] |
| *C. difficile* $R20291_{CM210}$ Δ*pyrE/pyrE* | R20291 isogenic *pyrE* mutant complemented with wild type *pyrE* into the *pyrE* loci | [5] |
| *C. difficile* $R20291_{CM196}$ | Hypersporulating strain derivative of R20291 strain (CM196; DMSO 435) | [6] |
| *C. difficile* $R20291_{CM196}$ Δ*pyrE* | R20291 hypersporulating sporulating strain isogenic *pyrE* mutant | This work |
| *C. difficile* $R20291_{CM196}$ Δ*pyrE/pyrE* | R20291 hypersporulating sporulating strain isogenic *pyrE* mutant complemented with wild type *pyrE* into *pyrE* loci | This work |
| *C. difficile* $R20291_{CM196}$ Δ*pyrE/pyrE+ tetR*-$P_{rbr}$-$P_{bclA1}$-*48aa*-$ntd_{bclA1}$-*nLuc* | $R20291_{CM196}$ derivative strain engineered by inserting *48aa*-$ntd_{bclA1}$-*nLuc* fusion under the control of the native *bclA1* promoter, and the *tetR* gene regulated by the *rubrerythrin* promoter ($P_{rbr}$) in reverse-complement orientation into the *pyrE* loci. | This work |
| *C. difficile* $R20291_{CM196}$ Δ*pyrE/pyrE+ tetR*-$P_{rbr}$-$P_{bclA1}$-*tetO-48aa*-$ntd_{bclA1}$-*nLuc* | $R20291_{CM196}$ derivative strain engineered by inserting *48aa*-$ntd_{bclA1}$-*nLuc* fusion under the control of the *bclA1* promoter with *tet* operator site (*tetO*), and the *tetR* gene regulated by the *rubrerythrin* promoter ($P_{rbr}$) in reverse-complement orientation into the *pyrE* loci. | This work |
| *C. difficile* $R20291_{CM196}$ Δ*pyrE/pyrE+ tetR*-$P_{rbr}$-*tetO*-$P_{bclA1}$-*48aa*-$ntd_{bclA1}$-*nLuc* | $R20291_{CM196}$ derivative strain engineered by inserting *48aa*-$ntd_{bclA1}$-*nLuc* fusion under the control of the native *bclA1* promoter, and the *tetR* gene regulated by the *rubrerythrin* promoter ($P_{rbr}$) with *tet* operator site (*tetO*) in reverse-complement orientation into the *pyrE* loci. | This work |
| *C. difficile* $R20291_{CM196}$ Δ*pyrE/pyrE+ tetR*-$P_{rbr}$-*tetO*-$P_{bclA1}$-*tetO-48aa*-$ntd_{bclA1}$-*nLuc* | $R20291_{CM196}$ derivative strain engineered by inserting *48aa*-$ntd_{bclA1}$-*nLuc* fusion under the control of the *bclA1* promoter with *tet* operator site (*tetO*), and the *tetR* gene regulated by the *rubrerythrin* promoter ($P_{rbr}$) with *tet* operator site (*tetO*) in reverse-complement orientation into the *pyrE* loci. | This work |
| *C. difficile* $R20291_{CM196}$ Δ*pyrE/pyrE+ tetR*-$P_{rbr}$-$P_{cdeC}$-*48aa*-$ntd_{bclA1}$-*nLuc* | $R20291_{CM196}$ derivative strain engineered by inserting *48aa*-$ntd_{bclA1}$-*nLuc* fusion under the control of the native *cdeC* promoter, and the *tetR* gene regulated by | This work |

| | | |
|---|---|---|
| | the *rubrerythrin* promoter ($P_{rbr}$) in reverse-complement orientation into the *pyrE* loci. | |
| *C. difficile* R20291$_{CM196}$ Δ*pyrE/pyrE+ tetR*-$P_{rbr}$-$P_{cdeC}$-*tetO-48aa-ntd*$_{bclA1}$-*nLuc* | R20291$_{CM196}$ derivative strain engineered by inserting *48aa-ntd*$_{bclA1}$-*nLuc* fusion under the control of the *cdeC* promoter with *tet* operator site (*tetO*), and the *tetR* gene regulated by the *rubrerythrin* promoter ($P_{rbr}$) in reverse-complement orientation into the *pyrE* loci. | This work |
| *C. difficile* R20291$_{CM196}$ Δ*pyrE/pyrE+ tetR*-$P_{rbr}$-*tetO*-$P_{cdeC}$-*48aa-ntd*$_{bclA1}$-*nLuc* | R20291$_{CM196}$ derivative strain engineered by inserting *48aa-ntd*$_{bclA1}$-*nLuc* fusion under the control of the native *cdeC* promoter, and the *tetR* gene regulated by the *rubrerythrin* promoter ($P_{rbr}$) with *tet* operator site (*tetO*) in reverse-complement orientation into the *pyrE* loci. | This work |
| *C. difficile* R20291$_{CM196}$ Δ*pyrE/pyrE+ tetR*-$P_{rbr}$-*tetO*-$P_{cdeC}$-*tetO-48aa-ntd*$_{bclA1}$-*nLuc* | R20291$_{CM196}$ derivative strain engineered by inserting *48aa-ntd*$_{bclA1}$-*nLuc* fusion under the control of the *cdeC* promoter with *tet* operator site (*tetO*), and the *tetR* gene regulated by the *rubrerythrin* promoter ($P_{rbr}$) with *tet* operator site (*tetO*) in reverse-complement orientation into the *pyrE* loci. | This work |
| *C. difficile* R20291$_{CM210}$ Δ*pyrE/pyrE+* $P_{cdeC}$-*48aa-ntd*$_{bclA1}$-*nLuc* | R20291$_{CM210}$ derivative strain engineered by inserting *48aa-ntd*$_{bclA1}$-*nLuc* fusion under the control of the native *cdeC* promoter into the *pyrE* loci. | This work |
| *C. difficile* R20291$_{CM210}$ Δ*pyrE/pyrE+* $P_{cdeC}$-*193aa-ntd*$_{bclA1}$-*nLuc* | R20291$_{CM210}$ derivative strain engineered by inserting *193aa-ntd*$_{bclA1}$-*nLuc* fusion under the control of the native *cdeC* promoter into the *pyrE* loci. | This work |
| *C. difficile* R20291$_{CM196}$ Δ*pyrE/pyrE+* $P_{cdeC}$-*48aa-ntd*$_{bclA1}$-*mNeonGreen* | R20291$_{CM196}$ derivative strain engineered by inserting *48aa-ntd*$_{bclA1}$-*mNeonGreen* fusion under the control of the native *cdeC* promoter into the *pyrE* loci. | This work |
| *C. difficile* R20291$_{CM196}$ Δ*pyrE/pyrE+* $P_{cdeC}$ -193*aa-ntd*$_{bclA1}$-*mNeonGreen* | R20291$_{CM196}$ derivative strain engineered by inserting *193aa-ntd*$_{bclA1}$-*mNeonGreen* fusion under the control of the native *cdeC* promoter into the *pyrE* loci. | This work |
| *C. difficile* R20291$_{CM210}$ Δ*pyrE/pyrE+* $P_{cdeC}$-*48aa-ntd*$_{bclA1}$-*mScarlet-i3* | R20291$_{CM210}$ derivative strain engineered by inserting *48aa-ntd*$_{bclA1}$-*mScarlet-i3* fusion under the control of the native *cdeC* promoter into the *pyrE* loci. | This work |
| *C. difficile* R20291$_{CM210}$ Δ*pyrE/pyrE+* $P_{cdeC}$-*193aa-ntd*$_{bclA1}$-*mScarlet-i3* | R20291$_{CM210}$ derivative strain engineered by inserting *193aa-ntd*$_{bclA1}$-*mScarlet-i3* fusion under the control of the native *cdeC* promoter into the *pyrE* loci. | This work |

(1) Emerson, J. E.; Reynolds, C. B.; Fagan, R. P.; Shaw, H. A.; Goulding, D.; Fairweather, N. F. A novel genetic switch controls phase variable expression of CwpV, a *Clostridium difficile* cell wall protein. *Mol Microbiol* **2009**, *74* (3), 541-556.
(2) McEllistrem, M. C.; Carman, R. J.; Gerding, D. N.; Genheimer, C. W.; Zheng, L. A hospital outbreak of *Clostridium difficile* disease associated with isolates carrying binary toxin genes. *Clin Infect Dis* **2005**, *40* (2), 265-272.

(3) Ehsaan, M.; Kuehne, S. A.; Minton, N. P. *Clostridium difficile* Genome Editing Using *pyrE* Alleles. *Methods Mol Biol* **2016**, *1476*, 35-52.
(4) Ng, Y. K.; Ehsaan, M.; Philip, S.; Collery, M. M.; Janoir, C.; Collignon, A.; Cartman, S. T.; Minton, N. P. Expanding the Repertoire of Gene Tools for Precise Manipulation of the *Clostridium difficile* Genome: Allelic Exchange Using *pyrE* Alleles. *PLOS ONE* **2013**, *8* (2), e56051.
(5) Castro-Córdova, P.; Mora-Uribe, P.; Reyes-Ramírez, R.; Cofré-Araneda, G.; Orozco-Aguilar, J.; Brito-Silva, C.; Mendoza-León, M. J.; Kuehne, S. A.; Minton, N. P.; Pizarro-Guajardo, M.; et al. Entry of spores into intestinal epithelial cells contributes to recurrence of *Clostridioides difficile* infection. *Nat Commun* **2021**, *12* (1), 1140.
(6) Cid-Rojas, F.; Paredes-Sabja, D. Characterization of a hypersporulating strain derivative of *Clostridioides difficile* R20291. *bioRxiv* **2025**, 2025.2011.2025.690273.
