# Supplementary material for: Development and Evaluation of Exosporium-Anchored Bioluminescent and Fluorescent Reporters for Tracking Clostridioides difficile Spores Formed In Vivo
Source: ACS Synth Biol. 2026 May 15;15(6):2338–55. doi: 10.1021/acssynbio.5c00961 (PMC13288923; doi:10.1021/acssynbio.5c00961)
Supplement: Supplementary file 5 [file sb5c00961_si_005.pdf]

TABLE S2. Plasmids used

Plasmid	Relevant characteristic	Source/Reference
pMTL-YN4	Pseudo suicide vector containing: *Clostridium perfringens catP* resistant cassette, *Clostridium sporogenes pyrE*; Carries unaltered *colE1 E. coli* replicon; *traJ* encoding transfer function of the RP4 *oriT* region; *repA* and *orf2*, the replication region of the *Clostridium botulinum* plasmid pBP1; and AscI/Sbf1 sites for the cloning of the right-hand homology arm/left-hand homology arm cassette.	[1, 2]
pMTL-YN2	Pseudo-suicide vector containing: *Clostridium perfringens catP* resistance cassette; *Clostridium sporogenes pyrE*; carries unaltered *colE1 E. coli* replicon; *traJ* encoding transfer function of the RP4 *oriT* region; *repA* and *orf2*, the replication region of the *Clostridium botulinum* plasmid pBP1; and FseI/SbfI sites for cloning of the right-hand homology arm/left-hand homology arm cassette.	[1, 2]
pMTL-YN4-Δ*pyrE*	pMTL-YN4 vector containing Δ*pyrE* loci from R20291 Δ*pyrE* to delete last 235bp of *pyrE*. Constructed by cloning a 2109bp fragment containing the Δ*pyrE* loci between AscI and SbfI sites of pMTL-YN4.	This work
pMTL-YN2C	Pseudo suicide vector containing: *Clostridium perfringens catP* cassette; *Clostridium sporogenes pyrE;* Carries unaltered *colE1 E. coli* replicon; *traJ* encoding transfer function of the RP4 *oriT* region; *repA* and *orf2*, the replication region of the *Clostridium botulinum* plasmid pBP1; a left-hand homology arm encompassing a 300 bp internal fragment of the R20291 *pyrE* gene lacking 50 nucleotides from the 5´-end, and 235 bp from the 3´-end; a right-hand homology arm compromising the 1200 bp region of DNA immediately downstream of *pyrE*; this plasmid also has additional DNA segments inserted between the left-hand and right-hand homology arms which carries a copy of the *lacZ´* containing a multiple cloning site region and a transcriptional terminator of the *ferredoxin* gene.	[1, 2]
2485<br>pMTL-YN2C+TT	pMTL YN2C plasmid was digested with NotI and KpNI as well as gBlock containing the native transcriptional terminator of the *pyrE* gene in addition introduced SalI site to be used for constructing transcriptional fusions., 164bp fragment was Gibson cloned into plasmid to yield pMTL-YN2C-TT.	Unpublished data
1492<br>pBH-BclA1-repaired	Repaired *bclA1* gene of R20291 in pBlue-Heron vector. SNPs that generated early stop codons in R20291 *bclA1* gene were fixed (T145A + T739C).	This work
1538<br>pBH-*tetR*-P_{rbr}-P_{bclA1}-*tetO-48aa-ntd*$_{bclA1}$*-nLuc*	pBlueHeron vector containing *tetR* under the *rubrerythrin* promoter and *48aa-ntd*$_{bclA1}$*-nLuc* under the *bclA1* promoter with *tetO* operator (2195 bp fragment).	This work
1543<br>pBH-P_{bclA1}-*tetO-193aa-ntd*$_{bclA1}$*-nLuc*	pBlueHeron vector containing *193aa-ntd*$_{bclA1}$*-nLuc* under the *bclA1* promoter with *tetO* operator (968 bp fragment).	This work
1618<br>pBH-*tetR*-P_{rbr}-P_{cdeC}-*tetO-48aa-ntd*$_{bclA1}$*-nLuc*	pBlueHeron vector with *48aa-ntd*$_{bclA1}$*-nLuc*, under the *cdeC* promoter with *tetO* operator.	This work

2282 pBH-*tetR*-P_{rbr}-*tetO*-P_{bclA1}-*tetO-48aa-ntd*$_{bclA1}$*-nLuc*	pBlueHeron vector containing *tetR* under the *rubrerythrin* promoter with *tetO* operator and *48aa-ntd*$_{bclA1}$*-nLuc* under the *bclA1* promoter with *tetO* operator. Constructed by inserting *tetO* into the *rubrerythrin* promoter region amplified from pBH-*tetR*-P_{rbr}-P_{bclA1}-*tetO-48aa-ntd*$_{bclA1}$*-nLuc*.	This work
2332 pBH-*tetR*-P_{rbr}-P_{bclA1}-*48aa-ntd*$_{bclA1}$*-nLuc*	pBlueHeron vector containing *tetR* under the *rubrerythrin* promoter and *48aa-ntd*$_{bclA1}$*-nLuc* under the native *bclA1* promoter. Constructed by cloning a 345bp fragment containing the native *bclA1* promoter between XbaI and BamHI sites of pBH-*tetR*-P_{rbr}-P_{bclA1}-*tetO-48aa-ntd*$_{bclA1}$*-nLuc*.	This work
2333 pBH-*tetR*-P_{rbr}-*tetO*-P_{bclA1}-*48aa-ntd*$_{bclA1}$*-nLuc*	pBlueHeron vector containing *tetR* under the *rubrerythrin* promoter with *tetO* operator and *48aa-ntd*$_{bclA1}$*-nLuc* under the native *bclA1* promoter. Constructed by cloning a 345bp fragment containing the native *bclA1* promoter between XbaI and BamHI sites of pBH-*tetR*-P_{rbr}-*tetO*-P_{bclA1}-*tetO-48aa-ntd*$_{bclA1}$*-nLuc*.	This work
2334 pBH-*tetR*-P_{rbr}-P_{cdeC}-*48aa-ntd*$_{bclA1}$*-nLuc*	pBlueHeron vector containing *tetR* under the *rubrerythrin* promoter and *48aa-ntd*$_{bclA1}$*-nLuc* under the native *cdeC* promoter. Constructed by cloning a 362bp fragment containing the native *cdeC* promoter between XbaI and BamHI sites of pBH-*tetR*-P_{rbr}-P_{bclA1}-*tetO-48aa-ntd*$_{bclA1}$*-nLuc*.	This work
2335 pBH-*tetR*-P_{rbr}-*tetO*-P_{cdeC}-*48aa-ntd*$_{bclA1}$*-nLuc*	pBlueHeron vector containing *tetR* under the *rubrerythrin* promoter with *tetO* operator and *48aa-ntd*$_{bclA1}$*-nLuc* under the native *cdeC* promoter. Constructed by cloning a 362bp fragment containing the native *cdeC* promoter between XbaI and BamHI sites of pBH-*tetR*-P_{rbr}-*tetO*-P_{bclA1}-*tetO-48aa-ntd*$_{bclA1}$*-nLuc*.	This work
2330 pBH-*tetR*-P_{rbr}-*tetO*-P_{cdeC}-*tetO-48aa-ntd*$_{bclA1}$*-nLuc*	pBlueHeron vector containing *tetR* under the *rubrerythrin* promoter with *tetO* operator and *48aa-ntd*$_{bclA1}$*-nLuc* under the *cdeC* promoter with *tetO* operator. Constructed by cloning a 548bp fragment containing the *cdeC* promoter with *tetO* from pBH-*tetR*-P_{rbr}-P_{cdeC}-*tetO-48aa-ntd*$_{bclA1}$*-nLuc* between XbaI and NdeI sites of pBH-*tetR*-P_{rbr}-*tetO*-P_{bclA1}-*tetO-48aa-ntd*$_{bclA1}$*-nLuc*.	This work
2397 pMTL-YN2C-*tetR*-P_{rbr}-P_{bclA1}-*48aa-ntd*$_{bclA1}$*-nLuc*	pMTL-YN2C vector containing *tetR* under the *rubrerythrin* promoter and *48aa-ntd*$_{bclA1}$*-nLuc* under the native *bclA1* promoter. Constructed by cloning a 2164bp EcoRI-NcoI fragment from pBH-*tetR*-P_{rbr}-P_{bclA1}-*48aa-ntd*$_{bclA1}$*-nLuc* between EcoRI and NcoI sites of pMTL-YN2C.	This work
2391 pMTL-YN2C-*tetR*-P_{rbr}-P_{bclA1}-*tetO-48aa-ntd*$_{bclA1}$*-nLuc*	pMTL-YN2C vector containing *tetR* under the *rubrerythrin* promoter and *48aa-ntd*$_{bclA1}$*-nLuc* under the *bclA1* promoter with *tetO* operator. Constructed by cloning a 2181bp EcoRI-NcoI fragment from pBH-*tetR*-P_{rbr}-P_{bclA1}-*tetO-48aa-ntd*$_{bclA1}$*-nLuc* between EcoRI and NcoI sites of pMTL-YN2C.	This work
2399 pMTL-YN2C-*tetR*-P_{rbr}-*tetO*-P_{bclA1}-*48aa-ntd*$_{bclA1}$*-nLuc*	pMTL-YN2C vector containing *tetR* under the *rubrerythrin* promoter with *tetO* operator and *48aa-ntd*$_{bclA1}$*-nLuc* under the native *bclA1* promoter. Constructed by cloning a 2183bp EcoRI-NcoI fragment from pBH-*tetR*-P_{rbr}-*tetO*-P_{bclA1}-*48aa-ntd*$_{bclA1}$*-nLuc* between EcoRI and NcoI sites of pMTL-YN2C.	This work
2500 pMTL-YN2C-*tetR*-P_{rbr}-*tetO*-P_{bclA1}-*tetO-48aa-ntd*$_{bclA1}$*-nLuc*	pMTL-YN2C vector containing *tetR* under the *rubrerythrin* promoter with *tetO* operator and *48aa-ntd*$_{bclA1}$*-nLuc* under the *bclA1* promoter with *tetO* operator. Constructed by cloning a 2200bp EcoRI-NcoI fragment from pBH-*tetR*-	This work

	P$_{rbr}$-*tetO*-P$_{bclA1}$-*tetO-48aa-ntd*$_{bclA1}$*-nLuc* between EcoRI and NcoI sites of pMTL-YN2C.	
2398 pMTL-YN2C-*tetR*-P$_{rbr}$-P$_{cdeC}$-*48aa-ntd*$_{bclA1}$*-nLuc*	pMTL-YN2C vector containing *tetR* under the *rubrerythrin* promoter and *48aa-ntd*$_{bclA1}$*-nLuc* under the native *cdeC* promoter. Constructed by cloning a 2181bp EcoRI-NcoI fragment from pBH-*tetR*-P$_{rbr}$-P$_{cdeC}$-*48aa-ntd*$_{bclA1}$*-nLuc* between EcoRI and NcoI sites of pMTL-YN2C.	This work
2392 pMTL-YN2C-*tetR*-P$_{rbr}$-P$_{cdeC}$-*tetO-48aa-ntd*$_{bclA1}$*-nLuc*	pMTL-YN2C vector containing *tetR* under the *rubrerythrin* promoter and *48aa-ntd*$_{bclA1}$*-nLuc* under the *cdeC* promoter with *tetO* operator. Constructed by cloning a 2198bp EcoRI-NcoI fragment from pBH-*tetR*-P$_{rbr}$-P$_{cdeC}$-*tetO-48aa-ntd*$_{bclA1}$*-nLuc* between EcoRI and NcoI sites of pMTL-YN2C.	This work
2400 pMTL-YN2C-*tetR*-P$_{rbr}$-*tetO*-P$_{cdeC}$-*48aa-ntd*$_{bclA1}$*-nLuc*	pMTL-YN2C vector containing *tetR* under the *rubrerythrin* promoter with *tetO* operator and *48aa-ntd*$_{bclA1}$*-nLuc* under the native *cdeC* promoter. Constructed by cloning a 2209bp EcoRI-NcoI fragment from pBH-*tetR*-P$_{rbr}$-*tetO*-P$_{cdeC}$-native-*48aa-ntd*$_{bclA1}$*-nLuc* between EcoRI and NcoI sites of pMTL-YN2C.	This work
2395 pMTL-YN2C-*tetR*-P$_{rbr}$-*tetO*-P$_{cdeC}$-*tetO-48aa-ntd*$_{bclA1}$*-nLuc*	pMTL-YN2C vector containing *tetR* under the *rubrerythrin* promoter with *tetO* operator and *48aa-ntd*$_{bclA1}$*-nLuc* under the *cdeC* promoter with *tetO* operator. Constructed by cloning a 2217bp EcoRI-NcoI fragment from pBH-*tetR*-P$_{rbr}$-*tetO*-P$_{cdeC}$-*tetO-48aa-ntd*$_{bclA1}$*-nLuc* between EcoRI and NcoI sites of pMTL-YN2C.	This work
2449 pMTL-YN1C-P$_{cwp2}$-*mNeonGreen*	pMTL-YN1C vector containing *mNeonGreen* under the control of the *cwp2* promoter.	[3]
2546 pMTL-YN2C-*tetR*-P$_{rbr}$-P$_{cdeC}$-*193aa-ntd*$_{bclA1}$*-nLuc*	pMTL-YN2C vector containing *tetR* under the *rubrerythrin* promoter and *193aa-ntd*$_{bclA1}$*-nLuc* under the native *cdeC* promoter. Constructed by cloning a 641bp fragment containing *193aa-ntd*$_{bclA1}$ and a 655bp fragment containing *nLuc* between BamHI and HindIII sites of pMTL-YN2C-*tetR*-P$_{rbr}$-P$_{bclA1}$-*48aa-ntd*$_{bclA1}$*.nLuc*.	This work
2682 pMTL-YN2C-TT-P$_{bclA1}$-*48aa-ntd*$_{bclA1}$*-nLuc*	pMTL-YN2C-TT vector containing *48aa-ntd*$_{bclA1}$*-nLuc* under the native *bclA1* promoter. Constructed by cloning a 1136bp XbaI-HindIII fragment from pYN2C-*tetR*-P$_{rbr}$-P$_{bclA1}$-*48aa-ntd*$_{bclA1}$*-nLuc* between XbaI and HindIII sites of pMTL-YN2C-TT.	This work
2669 pMTL-YN2C-TT-P$_{cdeC}$-*48aa-ntd*$_{bclA1}$*-nLuc*	pMTL-YN2C-TT vector containing *48aa-ntd*$_{bclA1}$*-nLuc* under the native *cdeC* promoter. Constructed by cloning a 1136bp XbaI-NcoI fragment from pBH-*tetR*-P$_{rbr}$-P$_{cdeC}$-*48aa-ntd*$_{bclA1}$*-nLuc* between XbaI and NcoI sites of pMTL-YN2C-TT.	This work
2709 pMTL-YN2C-TT-P$_{cdeC}$-*193aa-ntd*$_{bclA1}$*-nLuc*	pMTL-YN2C-TT vector containing *193aa-ntd*$_{bclA1}$*-nLuc* under the native *cdeC* promoter. Constructed by cloning a 636bp NcoI-HindIII fragment from pMTL-YN2C-*tetR*-P$_{rbr}$-P$_{cdeC}$-*193aa-ntd*$_{bclA1}$*-nLuc* between NcoI and HindIII sites of pMTL-YN2C-TT-P$_{cdeC}$-*193aa-ntd*$_{bclA1}$*-mNeonGreen*.	This work
2666 pMTL-YN2C-TT-P$_{cdeC}$-*48aa-ntd*$_{bclA1}$*-mNeonGreen*	pMTL-YN2C-TT vector containing *48aa-ntd*$_{bclA1}$*-mNeonGreen* under the native *cdeC* promoter. Constructed by cloning a 582bp fragment containing *48aa-ntd*$_{bclA1}$ and a 759bp fragment containing *mNeonGreen* between BamHI and XhoI sites of pMTL-YN2C-TT.	This work
2688 pMTL-YN2C-TT-P$_{cdeC}$-*193aa-ntd*$_{bclA1}$*-mNeonGreen*	pMTL-YN2C-TT vector containing *193aa-ntd*$_{bclA1}$*-mNeonGreen* under the native *cdeC* promoter. Constructed by cloning a 1024bp fragment containing *193aa-ntd*$_{bclA1}$ and a 759bp fragment containing *mNeonGreen* between BamHI and XhoI sites of pMTL-YN2C-TT.	This work

2786 pMTL-YN1C-P$_{cwp2}$-*mScarlet-i3*	pMTL-YN1C mutator plasmid containing *mScarlet-i3* under the control of the *cwp2* promoter for complementation at the *pyrE* locus.	3
2854 pMTL-YN2C-TT-P$_{cdeC}$-*48aa-ntd*$_{bclA1}$-*mScarlet-i3*	pMTL-YN2C-TT vector containing *48aa-ntd*$_{bclA1}$-*mScarlet-i3* under the native *cdeC* promoter. Constructed by cloning a 587bp fragment containing *48aa-ntd*$_{bclA1}$ and a 739bp fragment containing *mScarlet-i3* between BamHI and XhoI sites of pMTL-YN2C-TT.	This work
2855 pMTL-YN2C-TT-P$_{cdeC}$-*193aa-ntd*$_{bclA1}$-*mScarlet-i3*	pMTL-YN2C-TT vector containing *193aa-ntd*$_{bclA1}$-*mScarlet-i3* under the native *cdeC* promoter. Constructed by cloning a 1024bp fragment containing *193aa-ntd*$_{bclA1}$ and a 739bp fragment containing *mScarlet-i3* between BamHI and XhoI sites of pMTL-YN2C-TT.	This work

(1) Ehsaan, M.; Kuehne, S. A.; Minton, N. P. *Clostridium difficile* Genome Editing Using *pyrE* Alleles. *Methods Mol Biol* **2016**, *1476*, 35-52.

(2) Ng, Y. K.; Ehsaan, M.; Philip, S.; Collery, M. M.; Janoir, C.; Collignon, A.; Cartman, S. T.; Minton, N. P. Expanding the Repertoire of Gene Tools for Precise Manipulation of the *Clostridium difficile* Genome: Allelic Exchange Using *pyrE* Alleles. *PLOS ONE* **2013**, *8* (2), e56051.

(3) Donnelly, M. L.; Shrestha, S.; Ribis, J. W.; Kuhn, P.; Krasilnikov, M.; Alves Feliciano, C.; Shen, A. Development of a Dual-Fluorescent-Reporter System in *Clostridioides difficile* Reveals a Division of Labor between Virulence and Transmission Gene Expression. *mSphere* **2022**, *7* (3), e0013222.
