# Supplementary material for: Development and Evaluation of Exosporium-Anchored Bioluminescent and Fluorescent Reporters for Tracking Clostridioides difficile Spores Formed In Vivo
Source: ACS Synth Biol. 2026 May 15;15(6):2338–55. doi: 10.1021/acssynbio.5c00961 (PMC13288923; doi:10.1021/acssynbio.5c00961)
Supplement: Supplementary file 6 [file sb5c00961_si_006.pdf]

TABLE S3. Primers used for plasmid construction

Code	Primer Name	Primer Sequence
p386	16S rRNA	GTG CGG CTG GAT CAC CTC CT
p387	23S rRNA	CCC TGC ACC CTT AAT AAC TTG ACC
p528	FP-pyrE detection	AGAGAAGGAATAAAAAGTTTAGACGAAATAAGAGG
p529	RP-pyrER detection	TTACATCCCTAATTCCTTGAACTCTC
p631	FP-P.BclA1Ra.Sbf	CCCCCTGCAGGGGTACAGGAATGGGTGGCC
p632	RP-P-BclA1Ra	GTCTTTTAATATATCTGGTATTCTCACTTTAAAAAAAGTAATCAAC
p633	FP-BclA1Rb	GTGAGAATACCAGATATATTAAAAGACAAAAGTATATTAAAAGCAG
p634	RP-BclA1Rb	GTTATACTTGAACTATTTCCTATTATTGTTTGCCAACCTG
p635	FP-BclA1Rc	ACAATAATAGGAAATAGTTCAAGTATAACAGTAGATGCAAATGG
p636	RP-BclA1Rc.AscI	GAACGGCGCGCCATCTATTAAACCCATCTAAAAAGAATGTTG
p883	FP-BclA10337-Prom	ACT TCT AGA TTG TTA TGG TAA TAT ATT CTA TTT ACA ATT TTA TAA CTT TGG
p884	RP-BclA10337-Prom	ACT GGA TCC TCC CTC CAT TTT GGG TCT ATA TCA TAC
p887	FP-cdeC-Prom	ACT TCT AGA ATA CGA TAA GGC TTT TTT AAA TTC AAC CTA CAA ATG
p888	RP-cdeC-Prom	ACT GGA TCC TTG CCC TCC CTG TAA GCG ATT C
p921	FP pyrD	ATACCTGTAATGGGAATGGGCGG
p924	RP 0190	GGGAGGATGGTTCTGGAACCAG
p946	RP pyrE lacZ det	CGGGTACCGAGCTCGAATTCG
p947	FP pyrE det	CTT CAG GAA AAC ACA GTA ATA GAT ACG TAC
p974	FP DpyrE-LHA GC	GACCGATCGGGCCCCCTGCAGGTGGGAATGGGCGGAATAACTAAAG CC
p975	RP DpyrE-RHA GC	CTAAGGATTCAGAACGGCGCGCCGTCTGTCATAGAGCTTGGAAGCA GC
p976	RP Prbr+tetO	TGA GGG ATA GTC ACT ATC TCT ATA TAT AAA ATT TAG GAG GAA ACT CGA G
p977	FP Prbr+tetO	TAG AGA TAG TGA CTA TCC CTC AAA TTA AAT TTT TTA TTT ATT AAG TTA GTC TAT ATA TAC
p978	FP TetR	GGC CGC GAA TTC CAA AAA GG
p979	RP PbclA1	CTA TGT TTA AAA TAC ATG CTT AGT TAC AAT AC
p1076	RP Nluc-HindIII	TCA AAG CTT CTA TGC TAG AAT ACG TTC ACA AAG TCT C
p1223	2913 3' XhoI mNeonGreen Gibson	GCC AAG CTT GCA TGT CTG CAG GCC TCG AGT TAT TTA TAT AAT TCA TCC ATA CCC ATT AC
p1277	FP-NTD_BclA1_193aa_BamHI-GC	GAA TCG CTT ACA GGG AGG GCA AGG GAT CCA TGA GAA AAA TTA TAC TTT ATT TAA ATG

p1278	RP-NTD_BclA1_193aa_NcoI-GC	TCC ACC TCC TGC ACC TCC TGC AGC ACC CCA TGG TGT AAT TCT AAT TGG AAG ACA CTC AC
p1279	FP-Nluc_SalI-GC	GTG CTG CAG GAG GTG CAG GAG GTG GAG TCG ACA TGA GAC CAA GTA AAA AAT TAT TAA TAG
p1280	RP-Nluc_HindIII-GC	GTA AAA CGA CGG CCA GTG CCA AGC TTC TAT GCT AGA ATA CGT TCA CAA AG
p1359	FP PcdeC-NTD-BclA1	TCG AGC TCG GTA CCC GGG TCT AGA ATA CGA TAA GGC TTT TTT AAA TTC AAC CTA C
p1360	RP PcdeC-NTD-BclA1 for NeonGreen	CCA TAT TAT CTT CTT CTC CTT TAC TTA CCA TAT GTC CAC CTC CTG CAC CTC
p1361	FP NeonGreen for YN2C-TT-PcdeC-NTD-BclA1-NeonGreen	AGG TGC AGG AGG TGG ACA TAT GGT AAG TAA AGG AGA AGA AGA TAA TAT GG
p1523	RP PcdeC-NTD-BclA1 for mScarlet i3	CAT AAA TTC TTT TAT TAC TGC TTC TGT ACT ATC CAT ATG TCC ACC TCC TGC ACC TC
p1524	FP mScarlet-i3	AGG TGC AGG AGG TGG ACA TAT GGA TAG TAC AGA AGC AGT AAT AAA AGA ATT TAT G
p1525	RP 2912 3' XhoI mScarlet-i3 Gibson	TGC CAA GCT TGC ATG TCT GCA GGC CTC GAG TTA ACT TCT TCC ACT TCC TCC TGT AC
